# Global turnover of histone post-translational modifications and variants in human cells

**DOI:** 10.1186/1756-8935-3-22

**Published:** 2010-12-06

**Authors:** Barry M Zee, Rebecca S Levin, Peter A DiMaggio, Benjamin A Garcia

**Affiliations:** 1 Department of Molecular Biology, Princeton University, Princeton NJ, 08544, USA; 2 Department of Chemistry, Princeton University, Princeton NJ, 08544, USA

## Abstract

**Background:**

Post-translational modifications (PTMs) on the N-terminal tails of histones and histone variants regulate distinct transcriptional states and nuclear events. Whereas the functional effects of specific PTMs are the current subject of intense investigation, most studies characterize histone PTMs/variants in a non-temporal fashion and very few studies have reported kinetic information about these histone forms. Previous studies have used radiolabeling, fluorescence microscopy and chromatin immunoprecipitation to determine rates of histone turnover, and have found interesting correlations between increased turnover and increased gene expression. Therefore, histone turnover is an understudied yet potentially important parameter that may contribute to epigenetic regulation. Understanding turnover in the context of histone modifications and sequence variants could provide valuable additional insight into the function of histone replacement.

**Results:**

In this study, we measured the metabolic rate of labeled isotope incorporation into the histone proteins of HeLa cells by combining stable isotope labeling of amino acids in cell culture (SILAC) pulse experiments with quantitative mass spectrometry-based proteomics. In general, we found that most core histones have similar turnover rates, with the exception of the H2A variants, which exhibit a wider range of rates, potentially consistent with their epigenetic function. In addition, acetylated histones have a significantly faster turnover compared with general histone protein and methylated histones, although these rates vary considerably, depending on the site and overall degree of methylation. Histones containing transcriptionally active marks have been consistently found to have faster turnover rates than histones containing silent marks. Interestingly, the presence of both active and silent marks on the same peptide resulted in a slower turnover rate than either mark alone on that same peptide. Lastly, we observed little difference in the turnover between nearly all modified forms of the H3.1, H3.2 and H3.3 variants, with the notable exception that H3.2K36me2 has a faster turnover than this mark on the other H3 variants.

**Conclusions:**

Quantitative proteomics provides complementary insight to previous work aimed at quantitatively measuring histone turnover, and our results suggest that turnover rates are dependent upon site-specific post-translational modifications and sequence variants.

## Background

In eukaryotes, stable genetic storage is accomplished through the local organization of DNA around histone proteins to form the chromatin fiber. Histones have been long recognized as the structural scaffolds of chromatin, but more recent research has suggested that they possess a broader role. The epigenetic influence of histones is mediated primarily by post-translational modifications (PTMs) and also by selective deposition of histone variants, which in combination influence gene transcription and other processes such as DNA damage and replication [1]. In particular, histone PTMs such as trimethylation of lysine 4 on histone H3 (H3K4me3) recruit or displace other proteins that regulate transcription, such as the chromatin remodeler nucleosome remodeling factor (NURF) [2]. Although the underlying mechanism through which histone variants influence gene expression is unclear, certain histone variants have been shown to be linked with specialized genomic roles. For instance, replication-independent H3.3 variant deposition occurs at the transcriptional start sites in various organisms [3]. This specificity probably involves recognition by variant-specific remodeling complexes and chaperones, as is the case for Mis16 and Mis18 interaction with the centromere-specific H3 variant centromere protein (CENP)-A [4].

Implicit to the current theories of histone epigenetic regulation is that nucleosome occupancy over specific genomic regions is intimately linked to transcription [5]. The biological consequences of histone turnover were first explored with ^14^C-and ^3^H-radiolabeling, and among the findings was that specific histone pools were observed to turnover both dependently and independently of DNA replication [6,7]. It is now known that the majority of histone synthesis is synchronized with S-phase, and that H3.1 and H3.3 are deposited in a replication-dependent and-independent manner, respectively [8,9]. Expression of the histone genes, which are often clustered within chromosomes, is further regulated at the level of messenger (m)RNA expression, pre-mRNA processing and mRNA stability [10]. Offsetting histone synthesis and deposition is histone degradation and eviction; for instance *Saccharomyces cerevisiae *SWR1 and human SRCAP (sucrose non-fermentation (SNF)2 C-AMP response element binding protein binding protein (CBP) activator protein replaces H2A with H2A.Z in an ATP-dependent manner [11]. Excess production of histones is known to result in defects in mitotic chromosome segregation [12,13]. Thus, both histone synthesis/deposition and histone degradation/eviction must occur at approximately equal rates to maintain steady state DNA-bound histone levels and nucleosomal and genomic stability. Yet the absolute value of the rates for each process (synthesis/deposition and degradation/eviction) can differ significantly depending upon the enzyme and substrate. We describe the absolute values of these rates as turnover. In contrast to the relatively slow turnover of histones, which are known to have half-lives in the order of days as determined by radiolabeling studies, the rapid modification of histones after synthesis and incorporation into chromatin is known to be a rapid process [14].

More recent studies exploring histone turnover have mostly relied on tagging histones with green fluorescent protein, Myc or other epitopes to allow fluorescence recovery after photobleaching, chromatin immunoprecipitation or other techniques to measure turnover. One notable finding includes the existence of at least two pools of H1 with distinct DNA exchange rates in 3T3 cells [15]. Other studies involving the mapping of histone turnover to the genome have shown increased histone turnover on promoters relative to coding regions in *S. cerevisiae *[16], and on binding sites for trithorax group proteins relative to binding sites for polycomb group proteins in *Drosophila melanogaster *S2 cells [17,18]. These studies suggest that increased turnover within a particular genomic region disrupts the local chromatin environment and renders genes accessible to transcription factors, subsequently leading to gene activation. The results from *D. melanogaster *also point to an intriguing correlation between increased histone turnover and binding of the origin recognition complex, raising questions about the connection between DNA replication and chromatin [18].

A valuable complement to these ongoing investigations is the study of histone turnover when distinct site-specific PTMs and the specific histone variants are simultaneously considered. To obtain a quantitative measure of global histone turnover as a function of modification status and type of sequence variant, we designed a time course experiment using stable isotope labeling with amino acids in cell culture (SILAC) in conjunction with high-resolution mass spectrometry (MS). MS enables precise quantification of both histone post-translational modification sites, and allows sequence variants to be identified, thus we believe these attributes qualify MS as a useful technique for studying histone biology in general [19]. Furthermore, because we studied endogenous histones, there are no tags to interfere with higher-order chromatin structure and our measurements accurately capture global *in vivo *global histone turnover. In this study, we report that turnover rates of histone proteins vary widely depending upon the modification status and sequence variant. Our approach also produced important quantitative information, thus providing a useful and complementary platform for understanding chromatin biology.

## Results and discussion

**Figure 1:**
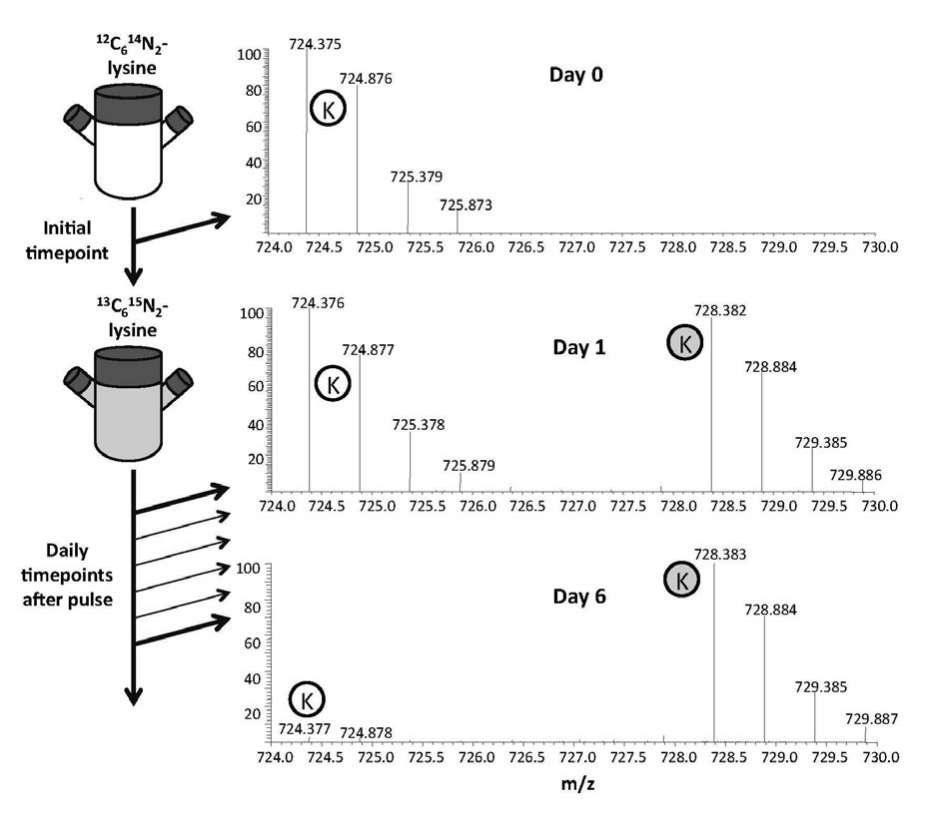
**Experimental design**. HeLa S3 cells were cultured in unlabeled ^12^C_6_^14^N_2_-lysine media, and transitioned into labeled ^13^C_6_^15^N_2_-lysine media. Daily samples were collected and analyzed by mass spectrometry (MS). MS spectra detected the ^13^C_6_^15^N_2_-lysine labeled peptide (gray circle, H3 73-83) co-eluting with the respective unlabeled peptide (white circle) after the pulse at days 0, 1 and 6.

**Figure 2:**
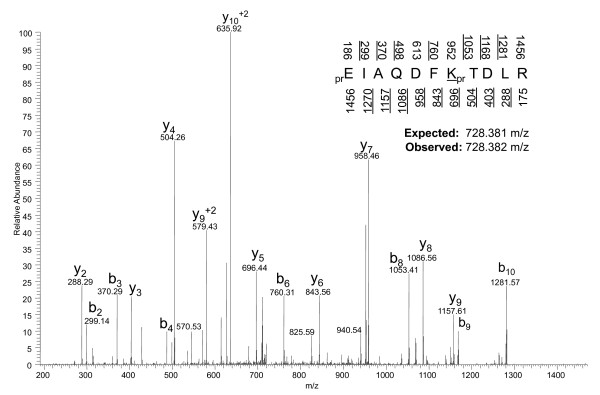
**Tandem mass spectrometry (MS/MS) spectrum of labeled lysine peptide**. MS/MS of the H3 73-83 peptide unmodified on K79 and labeled with ^13^C_6_^15^N_2_-lysine isotope. Underlined nominal masses above and below the sequence denote the *b *and *y *ions respectively that were annotated from the spectrum. The expected and observed mass to charge ratio (m/z) for the [M+2H^+^]^2+ ^precursor ion is provided. pr = Propionyl (heavy, D5-labeled).

To track the turnover of histone variants and PTMs in unsynchronized growing cells, we cultured HeLa cells in standard Joklik media and then transferred the cells to media containing exclusively ^13^C_6_^15^N_2_-lysine, which is essentially similar to performing pulse SILAC experiments (Figure 1). Histones found to contain the isotopically 'heavy' ^13^C_6_^15^N_2_-lysine residues were termed 'new', as they are synthesized after the introduction of the heavy media. This metabolic incorporation can be assessed by direct examination of the population of peptides on days 0, 1 and 6 (post incubation in heavy media) (Figure 1) At day 0 we observed a doubly charged peptide at 724.375 mass to charge ratio (m/z), which after tandem MS (MS/MS) experiments (data not shown) was found to correspond to the histone H3 73-83 residue peptide, _pr_EIAQDFK_pr_TDLR (pr = propionyl amide group from chemical derivatization). At day 1 we observed a decrease in this peptide, and the appearance of another peptide at 728.382 m/z. These peptides chromatographically co-elute and possess the same charge state, which is a characteristic of ^13^C^15^N labeled isotopic peptides, but the heavier peptide contains an overall mass shift that is an integer divisible of 8.011 Da (Δm/z = ~4 Da on a 2+ charge peptide). By day 6, the heavier peptide at 728.382 m/z was the most abundant species, and the lighter peptide at 724.375 m/z is almost completely gone (Figure 1). MS/MS interrogation of the peptide at 728.382 m/z (Figure 2) identified it as having the same sequence as the respective unlabeled peptide, but with all of the lysines substituted by the ^13^C_6_^15^N_2_-lysine. For all histone peptides that we studied in our MS experiments, we observed at least one corresponding isotopically labeled peptide during the time course (see Additional file 1, Additional file 2).

**Figure 3:**
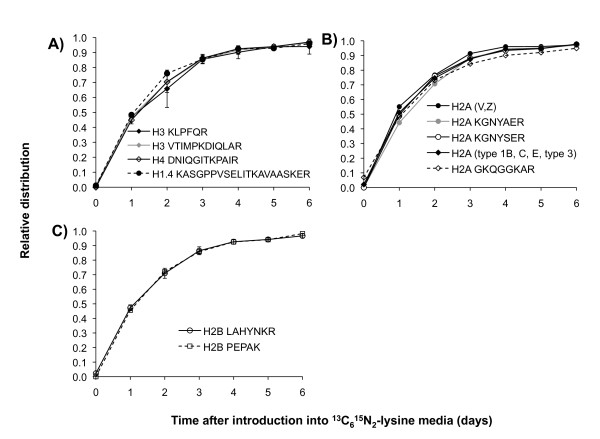
**Isotopic labeling of histone variants**. Average relative distribution (markers) of the fully isotopically labeled **(A) **H3, H4 and H1.4 peptides, **(B)** H2A peptides and **(C) **H2B variant peptides across the labeling time course. Note the gradual accumulation of the fully labeled peptide as time increases. The peptide labeled as H2A (V,Z) corresponds to the sequence ATIAGGGVIPHIHK, and the peptide labeled as H2A (types 1B, C, E and 3) corresponds to the sequence NDEELNKLLGR. All other peptides correspond to > 3 different histone proteins. Vertical lines represent standard deviation.

Additionally, on histone peptides containing two or more lysine residues, we detected peptide forms that were fully unlabeled (all ^12^C_6_^14^N_2_-lysines), fully labeled (all ^13^C_6_^15^N_2_-lysines) and partially labeled (a combination of ^12^C_6_^14^N_2_- and ^13^C_6_^15^N_2_-lysines) (see Additional file 3). In general, for all the peptides observed in all of the histone proteins, the partially labeled and fully labeled peptides accumulated in relative abundance over the time course and reached a final steady state level approximately 4 days after introduction into ^13^C_6_^15^N_2_-lysine heavy media (Figure 3), indicating that the intracellular lysine pool became increasingly populated with ^13^C_6_^15^N_2_-lysine. A minute fraction of ^12^C_6_^14^N_2_-lysine remained within the cells, presumably due to macromolecular decomposition during the timescale of our experiments, and was observed in both the partially labeled and fully unlabeled peptides.

**Figure 4:**
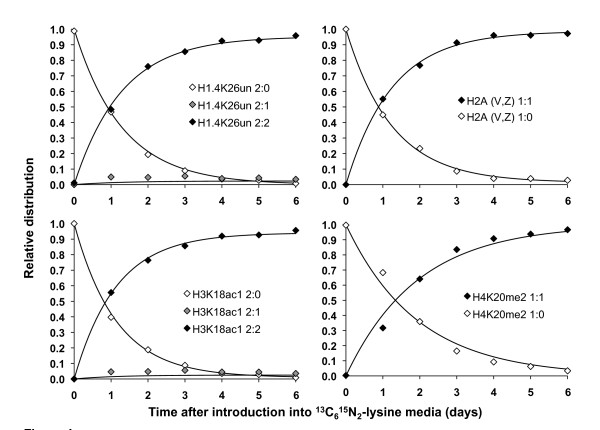
**Modeling of histone turnover**. Average relative distribution (markers) of various isotopically labeled and modified histone peptides on histones H1.4, H2A, H3 and H4 across the labeling time course (days). Lines represent optimized fits of the observed relative distributions for the particular peptide.

To model the rate of accumulation of the newly labeled histone peptides and the corresponding depletion rate of the old unlabeled peptides, we fitted the relative distributions of the different labeled states for each peptides to a set of differential equations similar to those previously published (Figure 4, see Additional file 4)[20]. Our model accounts for all states corresponding to the fully labeled, fully unlabeled and partially labeled peptides. An assumption implicit to our method of normalization and modeling is that the post-translationally modified forms, when summed across all isotopically labeled states, remain at steady state relative to each other. We confirmed that the standard deviations of the relative abundances for each modified peptide, summed over all the labeled states and across the time course, were < 0.051 (see Additional file 5). This is the threshold at which 95% of the observed variability cannot be accounted for by 10% of the instrument measurement variability, a common metric for accessing the reproducibility of MS experiments [21]. Based upon these measurements, we concluded that all of the peptides occurred at steady state throughout the experiment.

Next, we extrapolated the turnover rate for bulk histones (Figure 3, Table 1). For this purpose, we examined the 24-35 (DNIQGITKPAIR) H4 peptide, the 33-53 (KASGPPVSELITKAVAASKER) H1.4 peptide and the 65-70 (KLPFQR) and 117-128 (VTIMPKDIQLAR) H3 peptides (labeled amino acids underlined), which our laboratory has previously found are not modified for HeLa S3 (unpublished data). Thus, we postulated that the measured turnover rates for these peptides should reflect the general turnover rates of bulk H4, H3 and H1.4 histones, respectively. We found that H1.4 has a faster turnover than H3, which in turn has a slightly faster turnover than H4. These findings are consistent with our previous work, in which we labeled HeLa S3 cells with ^13^CD_3_-methionine and tracked incorporation of the heavy methionine isotope, with a 4.021 m/z shift for every new methionine added [22]. Histone H1.4 is known to repress transcription and condense chromatin more efficiently than other H1 subtypes, partly due to its increased chromatin binding affinity [23,24]. It is likely that the faster turnover of H1.4 compared with H3 and H4 is not due to a difference in epigenetic function, but rather to its different location relative to the core nucleosome. In the future, it would be interesting to examine how H1.4 turnover compares relative to other less efficient chromatin-compacting H1 subtypes, such as H1.1 and H1.2.

**Table 1:** Histone post-translational modification and variant-specific turnover.1

Peptide^2-4,6^	Turnover, per day^5^	Peptide^2-4,6^	Turnover, per day^5^
H3K65un	0.6230 ± 0.0001	H3K27unK36un	1.9213 ± 0.0001

H3K122un	0.6400 ± 0.0001	H3K27me1	1.1391 ± 0.0000

H3K4un	0.6638 ± 0.0001	H3K36me1	1.6913 ± 0.0000

H3K4me1	0.4863 ± 0.0000	H3K27me2	0.8207 ± 0.0000

H3K56un	0.6378 ± 0.0000	H3K36me2	1.0892 ± 0.0000

H3K56ac1	2.4335 ± 0.0014	H3K27me3	0.5148 ± 0.0000

H3K18K23un	0.6806 ± 0.0001	H3K27me1K36me2	0.7540 ± 0.0000

H3K18/K23ac1^4^	0.8793 ± 0.0000	H3K27me2K36me1	0.6210 ± 0.0001

H3K18ac1K23ac1	1.1446 ± 0.0001	H3K27me2K36me2	0.4537 ± 0.0000

H3K79un	0.6785 ± 0.0001	H3K27me3K36me1	0.3681 ± 0.0000

H3K79me1	0.4526 ± 0.0000	H3K27me1K36me3	0.4547 ± 0.0000

H3K79me2	0.3841 ± 0.0000	H4K5K8K12K16un	0.6495 ± 0.0000

H3K9unK14un	1.1335 ± 0.0001	H4K5/K8/K12/K16ac1^4^	0.7773 ± 0.0000

H3K9me1	0.7967 ± 0.0001	H4K5/K8/K12/K16ac2^4^	0.9819 ± 0.0000

H3K9me2	0.6620 ± 0.0000	H4K5/K8/K12/K16ac3^4^	1.0423 ± 0.0000

H3K9me3	0.4652 ± 0.0000	H4K5K8K12K16ac4	1.0056 ± 0.0000

H3K9/K14ac1^4^	1.3393 ± 0.0001	H4K20un	2.2672 ± 0.0005

H3K9me1K14ac1	0.9205 ± 0.0000	H4K20me1	1.3340 ± 0.0002

H3K9me2K14ac1	0.6454 ± 0.0000	H4K20me2	0.5177 ± 0.0000

H1.4K26un	0.7721 ± 0.0000	H4K20me3	0.3307 ± 0.0000

H4K31un	0.6182 ± 0.0001	H2A: ATIAGGGVIPHIHK	0.8016 ± 0.0001

H2A: GKQGGKAR	0.7222 ± 0.0000	H2A: NDEELNKLLGR	0.7135 ± 0.0000

H2A: KGNYAER	0.6311 ± 0.0000	H2B: LAHYNKR	0.6448 ± 0.0000

H2A: KGNYSER	0.7215 ± 0.0001	H2B: PEPAK	0.6293 ± 0.0000

^1^Absolute turnover values (mean ± standard deviation per day) extrapolated from the relative distribution of the isotopically labeled bulk H3, H4 and H1.4 peptides with a particular post-translational modification.

^2^me = methyl

^3^ac = acetyl

^4^The H2A and H2B turnover values are averages of all histone variants containing the specific peptide sequence.

^5^The model was iterated 200 times for each individual peptide, where the average and standard deviation were taken for parameters within 105% of the determined optimum parameter.

^6^For H3K18/K23ac1, H3K9/K14ac1, and H4K5/K8/K12/K16ac1, ac2 and ac3, the localization of the acetyl(s) on the multiple lysines was not determined.

We also examined peptides from several H2A and H2B variants. Similar to the aforementioned unmodified peptides for H1.4, H3 and H4, we never observed forms of the H2A or H2B peptides to be modified and thus reasoned that they also represented bulk turnover of their respective protein (Figure 3, Figure 4). However, some of the tryptic peptides are not unique to a particular variant, as many variants are largely homologous (Table 1). For example, we could only link the peptide NDEELNKLLGR, which is found in H2B types 1-C, 3 and 1-B/E, to the average turnover associated with all the homologous histones. We observed that the H2B variants containing the peptide sequence LAHYNKR or PEPAK had turnover rates similar to H3 and H4 (see Additional file 6). By contrast, a broader range of turnover values was found for the H2A variants, with some being notably faster than H1.4. This wide range of turnover values observed for the H2A variants suggests that different variants may serve different purposes in chromatin assembly. For instance, the human H2A variants that contain the sequence ATIAGGGVIPHIHK, which include H2AZ, have the fastest turnover (see Additional file 6). Intriguingly, H2AZ is known to localize specifically to transcriptional start sites, and the increased turnover is consistent with previous work showing that histones over promoters have a faster turnover than histones over the gene coding region [16,25]. It is important to note that H2AZ localization over promoters does not indicate that H2AZ is associated with gene activation, but is currently believed to bind to and prime silent promoters for subsequent activation [26]. Despite the differences in turnover values between the core histones, all the core peptides have a turnover rate in the order of ln(2) = 0.6931/day, which is approximately the expected rate if half of peptide population become labeled after each day. Thus, with few exceptions, bulk H1.4, H2A, H2B, H3 and H4 peptides generally turnover at a rate indistinguishable from the rate predicted from HeLa division approximately every 24 hours. This is consistent with previous work showing that most newly synthesized histones are deposited onto newly replicated DNA during S phase, and that the different histone families are synthesized in equal stoichiometry with each other [12,27].

To place our bulk histone turnover values in the context of previous work, our finding that specific H2A variants have faster turnover than the other core histones is consistent with previous radiolabeling work with tritiated amino acids in Friend murine erythroleukemia cells [28], Another radiolabeling study that administered tritiated water to mice and examined liver histones found that histone turnover generally occurs on the same time scale as cellular proliferation, which is in excess of 100 days for these cell types [29,30]. Despite the heterogeneity of cell types with vastly different proliferation rates in adult tissue, the whole-animal work is similar to our findings in HeLa cells; namely, that most bulk histones turn over with the cell cycle.

**Figure 5:**
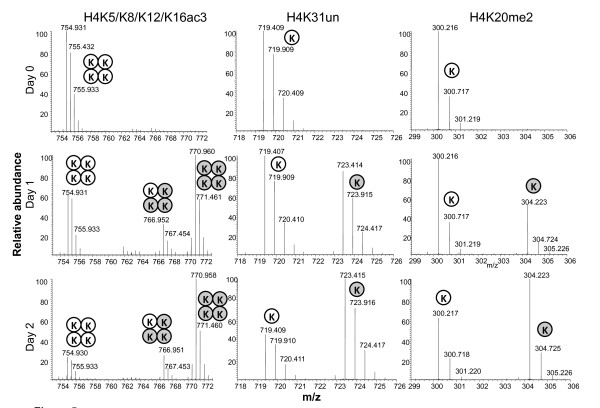
**Turnover of modified H4 peptides**. Mass spectrometry spectra of the H4 triacetylated 4-16 peptide (H4K5K8K12K16ac3), unmodified 24-35 peptide (H4K31un) and dimethylated 20-23 peptide (H4K20me2) during the labeling time course. Isotopic distributions of peptides that were ^12^C_6_^14^N_2_-lysine unlabeled (white circle) or ^13^C_6_^15^N_2_-lysine labeled (gray circle) are denoted on the spectra. Note the intermediate isotopic distribution (one white circle + three gray circles) of the 4-16 peptide has three of its four lysines labeled with ^13^C_6_^15^N_2_-lysine.

**Figure 6:**
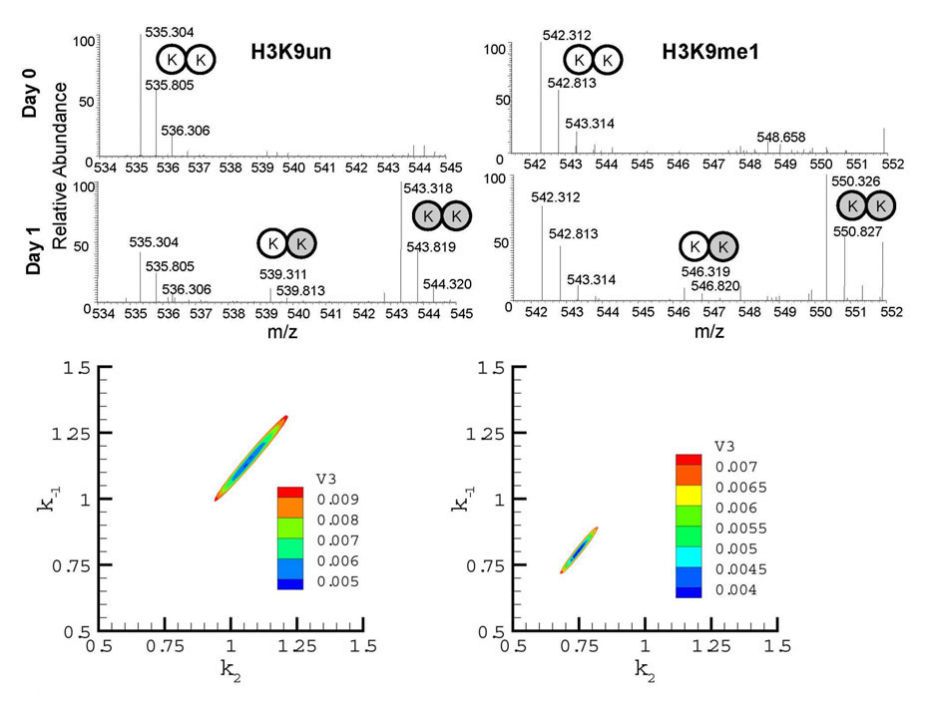
**Effect of post-translational modifications status on H3 turnover**. Mass spectrometry (MS) spectrum of the H3 9-17 peptide (left) unmodified and (right) monomethylated on K9 collected from cells before and 1 day after the labeling time course. Contour plots below the H3K9un and H3K9me1 MS spectra are shown for the optimization of the turnover parameter (k_-1_) versus the addition of two labeled lysines (k_2_), where the contours represent values (color bar) of the objective/error function of the model in explaining the variability of the data. The boundaries of the contours equal *z*(k) × (1+3/11 × *F*_0.05_(3,11)), where *z*(k) is the global minimum of the calculated objective value for the H3K9un and H3K9me1 optimization. Note the lack of overlap of the two contours.

We next examined histone turnover as a function of their PTM status (Figure 5, Figure 6, Table 2). We defined the relative turnover as the turnover with respect to the unmodified peptide, in contrast to the absolute turnover we have discussed previously. Among the notable findings, we observed that the relative turnover of acetylated histones is significantly faster than that of methylated histones (Wilcoxon rank sum, *p *= 6.1 × 10^-5^). For instance, a peptide containing H4K20me2 has a slower turnover rate than H4K31un (general H4 turnover), whereas the triacetylated H4K5/K8/K12/K16ac3 has a faster turnover than H3K31un (Figure 5,d Table 2). Furthermore, progressively acetylated and methylated peptides have a faster and slower turnover, respectively, than their unmodified peptide counterparts. For instance, the doubly acetylated H3K18acK23ac peptide has a faster turnover than the monoacetyl H3K18/K23ac1 peptide, which in turn has a faster turnover than the H3K18unK23un peptide (Table 2; see Additional file 7). Additionally, the monomethylated H3K9me1 peptide has a slower turnover than the respective unmodified peptide (Figure 6, Table 2). Interestingly, we also observed a trend for peptides with transcriptionally active PTMs, such as H3K36me2, to have faster relative turnovers than peptides with silent PTMs, such as H3K27me2 (Kruskal-Wallis, *p *= 6.3 × 10^-4^).

**Table 2:** Relative turnover of H3 and H4 modified peptides

Peptide^1,2^	Relative turnover^3^	Epigenetic Function^4,5^
H3K4me1	0.7326	A [52]
H3K9me1	0.7029	A [52]
H3K9me2	0.5840	S [52]
H3K9me3	0.4104	S [52]
H3K9/K14ac1	1.1816	A [38]
H3K18/K23ac1	1.2919	A [38]
H3K18ac1K23ac1	1.6818	A [38]
H3K27me1	0.5929	A [52]
H3K27me2	0.4272	S [52]
H3K27me3	0.2679	S [52]
H3K36me1	0.8803	A [52]
H3K36me2	0.5669	A [53]
H3K56ac1	3.8155	A [38]
H3K79me1	0.6671	A [54]
H3K79me2	0.5661	A [54]
H4K5/K8/K12/K16ac1	1.1968	A [38]
H4K5/K8/K12/K16ac2	1.5118	A [38]
H4K5/K8/K12/K16ac3	1.6048	A [38]
H4K5K8K12K16ac4	1.5483	A [38]
H4K20me1	0.5884	A [52]
H4K20me2	0.2283	S [55]
H4K20me3	0.1459	S [56]

^1^me = Methyl

^2^ac = Acetyl

^3^Relative turnover, with respect to the relevant unmodified peptide, is determined for various modified forms of H3 and H4.

^4^A = active marks

^5^S = silent marks

How acetylation and methylation structurally affect the nucleosome itself is not entirely clear. Some notable examples of a direct structural effect of histone PTMs and nucleosomal structure are the electrostatic interaction between the H4 tail with respect to H2A/H2B on the adjacent nucleosome [31], the electrostatic interaction between H3K56ac and the DNA backbone [32], and the general destabilization of the nucleosome bound to positively supercoiled DNA by the hyperacetylated histones H3 and H4 [33]. The acetyl moiety removes the positive charge from lysines due to resonance effects of the carbonyl group, whereas the methyl moiety stabilizes charge by raising the acid dissociation constant (pKa) of the remaining acidic protons. We believe that our PTM-specific data supports a general model in which changes to higher-order chromatin structure via charge stabilization or removal respectively impedes or facilitates, subsequent chromatin remodeling. ATP-dependent chromatin remodeling generally proceeds via three pathways: nucleosome sliding along the DNA, nucleosome conformational change, and nucleosome eviction from the DNA [34,35]. Because we measured turnover by quantifying the appearance of isotopically labeled histones after a pulse, our turnover measurements may reflect the activity of remodelers and chaperones responsible for histone eviction and replacement. To a lesser extent, our turnover measurements may also reflect how quickly the histones become modified into a different peptide; for instance, an unmodified peptide that becomes quickly acetylated would probably have a faster turnover than another peptide that is less rapidly modified.

When mechanistically considering histone turnover in the context of transcription, another non-mutually exclusive pathway for histone turnover emerges. Several models have proposed the displacement of the nucleosome encountered by RNA polymerase onto the upstream DNA strand [36] or even onto the nascent RNA strand, followed by rebinding onto DNA [37].We believe that our measurements reflect this nucleosomal event only to a small extent, as we tracked the incorporation of newly synthesized histones. In particular, given the reported high affinity of H2A and H2B for RNA, it is unlikely that our observed turnover of H2A and H2B occurs during the RNA transition state [37]. However, the lack of preferential affinity for either DNA or RNA by the H3/H4 tetramer as reported in the same study may facilitate H3/H4 turnover during this transition state when the tetramer is no longer bound to the H2A/H2B dimer.

A major strength of mass spectrometric analysis is the ability to simultaneously sequence and quantify multiple modifications on the same histone peptide. In the 9-17 peptide on histone H3 (KSTGGKAPR), we observed the presence of both methylation and acetylation on K9 and K14 in HeLa cells. Interestingly, for H3K9me1K14ac1, the turnover is faster than for the exclusively monomethylated H3K9me1 peptide, but slower than for the exclusively monoacetylated H3K9/K14ac1 peptide. For H3K9me2K14ac1, the turnover is slower than for both H3K9me2 and H3K9/K14ac1. These two observations suggest that histone acetylation, generally considered an active mark, is epistatic to active methyl marks (such as H3K9me1) yet antagonistic towards silent methyl marks [38]. The presence of both active and silent marks in the H3K27K36 peptide (KSAPATGGVKKPHR), such as H3K27me2K36me1, is also consistent with this trend. In particular, the H3K27me2K36me1 peptide has a much slower turnover than a peptide containing either the silent mark H3K27me2 or the active mark H3K36me1 (Table 1). Likewise, the H3K27me1K36me2 peptide has a slower turnover than a peptide containing either H3K27me1 or H3K36me2. However, it should be noted that the slower turnover of these doubly modified peptides is not believed to result simply from the total number of methyl groups, because the H3K27me1K36me2 peptide has a faster turnover than the H3K27me2K36me1 peptide.

The presence of antagonistic PTMs may result in a chromatin domain similar to bivalent domains, which contain histones bearing H3K4me3 and H3K27me3 marks, and are believed to poise genes for either activation or silencing [39]. In principle, the conversion of a bivalent domain to either a fully activating or silencing domain can be achieved by histone-modifying enzymes or replacement of the histone molecule with a new unmodified histone that becomes modified. Given that known bivalent domains are bound by polycomb proteins that methylate H3K27, the former mechanism explaining how bivalent domains function *in vivo *seems more likely [40]. Thus, a slower histone turnover would be expected if the bivalent domains switch epigenetic function through modification changes rather than protein eviction and exchange, consistent with our observations for the H3K9me2K14ac and H3K27me2K36me1 peptides. We believe a similar logic can be applied to binary switch domains; for instance, an effector molecule (that is, heterochromatin protein 1) recognizing methylation on H3K9 would engage in competitive binding against another effector molecular recognizing a phosphorylation on H3S10 [41].

Another layer of complexity for histone H3 is that it exists as three major variants: H3.1, H3.2 and H3.3, and the less abundant, centromere-specific variant CENP-A. Generally, H3.2 and H3.3 are associated with silent and active gene expression, respectively, whereas the evolutionarily younger H3.1 variant is associated with both expression states [42]. Because high-performance liquid chromatography (HPLC) fractionation is able to resolve H3.1, H3.2 and H3.3, we can examine the H3 variant specific turnover of peptides with particular modifications [43]. In general, we found that turnover of the modified peptides from the three H3 variants did not differ significantly from one other (Table 3). In quiescent human lymphocytes that were stimulated to re-enter the cell cycle, different histone H3 variants were observed to exchange with each other [29,44] Because our steady-state measurements were performed on actively dividing and asynchronous HeLa cells, it is likely that this difference would not be observed generally for H3 variant turnover. A notable exception is K36 dimethylation; the H3.2 peptide containing this modification has a greater turnover than H3.1 or H3.3. Previous studies have shown that K36me2 is generally enriched in H3.3 over H3.2 [45]. Based on our turnover data, we speculate that the elevated turnover of K36me2 in H3.2 is essential for maintaining this PTM at a low level, and that it is the absence of K36me2 on H3.2 rather than its presence on H3.3 that is crucial for epigenetic function. Future experiments to test this hypothesis could resort to substitution of site-directed mutagenesis, with K36 on H3.2 mutated to a cysteine and then alkylated with the appropriate reagent to mimic dimethylation. Wild-type H3.2 contains a single cysteine at position 110, which can be mutated to an alanine to prevent derivatization [46]. Similarly, a 'K36me1 mimic' can be made on H3.3 to prevent dimethylation [46].

**Table 3:** H3 variant-specific turnover.

Peptide^1-3^	Turnover^4^		
	
	H3.2/H3.1	H3.3/H3.1	H3.2/H3.3
K4un	1.0707	1.0437	1.0258

K4me1	1.0372	1.1400	0.9098

K79un	1.0598	1.0720	0.9887

K79me1	1.0251	1.0496	0.9767

K56un	1.0461	1.0170	1.0286

K9un	1.1022	0.9726	1.1332

K9me1	1.0462	1.0101	1.0357

K9me2	1.0507	0.9799	1.0723

K9me3	0.9925	1.0105	0.9822

K9me1K14ac1	1.1077	1.0187	1.0874

K9me2K14ac1	1.0116	0.9786	1.0337

K18un	1.0419	1.0086	1.0331

K18/K23ac1	1.0510	1.0140	1.0365

K18ac1K23ac1	1.0391	1.0374	1.0017

K65un	1.0745	1.0473	1.0259

K122un	1.0146	0.9936	1.0212

K27un	1.5204	1.9049	0.7982

K27me2	1.0330	1.0400	0.9933

K27me3	1.0057	1.1336	0.8872

K36me2	1.3324	0.9307	1.4316

K27me1K36me2	0.9910	1.0099	0.9813

K27me1K36me2	0.9777	1.1848	0.8252

^1^me = Methyl

^2^ac = Acetyl

^3^un = Unmodified

^4^Relative turnover of various post-translationally modified peptides from H3.1, H3.2 and H3.3 variants with respect to each other.

The unmodified 27-40 peptide that contains K27 and K36 also has a higher turnover in both H3.2 and H3.3 than inH3.1. We suspect that this is due to the fact that K27 and K36 become immediately methylated in both H3.2 and H3.3 variants respectively, as both PTMs are known to be enriched on the two variants [45]. Consequently, the unmodified peptide in the H3.2 and H3.3 fractions should turn over faster than in H3.1, because it is immediately methylated into a different peptide.

## Conclusion

Using mass spectrometry and SILAC, we found that histones are generally stable proteins, with the H2A variants exhibiting the largest range of global turnover rates, and H1.4 turnover being faster than that of H3 and H4. Exploring the relationship between post-translational modifications and turnover, we found that turnover is significantly greater when a histone peptide becomes acetylated than when it is methylated. When classifying the H3 and H4 modified peptides according to epigenetic function, we found that active marks have a significantly faster turnover than silent marks. However, the dual presence of a silencing and activating mark on the same peptide led to vastly distinct turnover rates compared with either mark alone. The various modified forms of the H3 variants (H3.1, H3.2 and H3.3) generally had a similar global turnover, with the notable exception of K36me2. In conclusion, this study offers novel insights into histone turnover by using techniques complementary to those already in standard use by the general chromatin biology community to examine the role of histone turnover in epigenetic regulation.

## Methods

### Cell culture maintenance and SILAC time course

HeLa S3 were maintained between 5-10 × 10^5 ^cells/ml throughout the experiment and, before the time course, were grown in minimum essential Joklik modified media (Sigma Aldrich, St. Louis, MO, USA) as previously described [22]. At the start of the time course, cultures were pelleted at 300 g for 3 minutes in a refrigerated centrifuge, decanted, and resuspended in Joklik media depleted of unlabeled lysine (ThermoScientific HyClone, Logan, UT, USA) and supplemented with L-lysine-^13^C_6_^15^N_2 _(Cambridge Isotope Laboratories Inc., Cambridge, MA, USA), 5% fetal bovine serum (ThermoScientific Hyclone), penicillin, streptomycin and 1% Glutamax (Invitrogen, Carlsbad, USA). Before resuspension, flasks were rinsed with Joklik media depleted of lysine. Every 24 hours for 6 days, half of the culture was separated by centrifugation at 600 g, washed twice with Tris-buffered saline, flash-frozen in liquid N_2_, and stored at -80°C. The culture was replenished with an approximately equal volume of labeled media after sample collection.

### Nuclei isolation and histone extraction

Cell pellets were thawed on ice before nuclei isolation and histone extractions as previously described [47]. Briefly, cells were lysed using NP-40 in nuclei isolation buffer with 5 μmol/l microcystin, 0.3 mmol/l 4-(2-aminoethyl) benzenesulfonyl fluoride hydrochloride (AEBSF) and 10 mmol/l sodium butyrate. Histones were isolated from nuclei by extraction with 0.4 N H_2_SO_4_, precipitated with trichloroacetic acid, washed in acetone, dried overnight and resuspended in water.

### Reversed-phase HPLC separation of bulk histone

Based on the Bradford assay, 125 μg of protein was allocated for one-pot propionic anhydride derivatization. The remaining extract was separated on a C18 column (4.6 mm internal diameter × 250 mm (Vydac, Hesperia, CA, USA) using HPLC (System Gold HPLC; Beckman Coulter, Fullerton, CA, USA) with a gradient of 30-60% B over 100 min, followed by 20 minutes at 100% B (buffer A was 5% acetonitrile in 0.2% trifluoroacetic acid (TFA), buffer B was 90% acetonitrile in 0.188% TFA) and a flow rate of 0.8 ml/min. Fractions spanning a single variant were pooled, and then dried to completion in a vacuum centrifuge.

### Histone preparation for MS analysis

The 125 μg bulk extract and HPLC-separated histone H3.1, H3.2, H3.3 and H4 were derivatized and desalted for MS as previously described, with the exception that the reagent was composed of 3:1 isopropanol:propionic anhydride instead of 3:1 methanol:propionic anhydride [48]. For HPLC-purified H1, H2A and H2B, samples were resuspended in 100 mmol/l ammonium bicarbonate (pH 6-7), digested using trypsin for 20 minutes with a 20:1 substrate/enzyme ratio, and subsequently propionylated.

### MS and MS/MS analysis

Samples were loaded by an autosampler (AS-2; Eksigent Technologies Inc., CA, USA) onto a 75 μm fused silica capillary column with ESI tip hand packed with 130 mm of C18 reverse phase resin (5 μm particles, 200Å pore size) (Magic C18; Michrom BioResources Inc., Auburn, CA, USA). Samples were resolved on a 110 minute 1-100% buffer B gradient (buffer A = 0.1 mol/l acetic acid, Buffer B = 70% acetonitrile in 0.1 mol/l acetic acid) at a flow rate of 0.070 ml/min controlled by an HPLC pump (1200 series; Agilent, Santa Rosa, CA, USA). The HPLC was coupled to a mass spectrometer (LTQ-Orbitrap; ThermoFisher Scientific, Carlsbad, CA, USA) with a resolution of 30,000 for full MS followed by seven data-dependent MS/MS analyses. Ions selected for MS/MS interrogation were placed on an exclusion list for 30 seconds. Targeted runs were performed on a number of samples to increase the identification of low-abundance modifications.

### Data analysis and modeling

Peptide abundance was calculated by manual chromatographic peak integration of full MS scans using Qual Browser software (version 2.0.7; ThermoFisher Scientific Inc.). Peptide sequence and modifications were confirmed by inspection of the MS/MS data. To identify histone H1, H2A and H2B peptides, a database search was performed using the SEQUEST algorithm within the Bioworks Browser (version 3.3; ThermoFisher Scientific). The search was performed against human histone variants for fully enzymatic tryptic digests, allowing for five missed cleavage sites due to the propionyl derivatization, propionylation of unmodified and monomethylated lysines and N-termini (+56.026 Da) and oxidation of methionine (+15.995 Da).

As a labeling convention, we appended each peptide with two numbers, the first referring to the total number of lysines and the second to the number of labeled lysines. For instance, H3K9me1 2:0, H3K9me1 2:1 and H3K9me1 2:2 refer to the same 9-17 monomethylated peptide containing 0, one and two isotopically labeled lysines, respectively. For quantifying the dynamics of histone turnover, we normalized the relative abundances of each labeled state with respect to all labeled states of the same modified peptide to determine the relative distribution of that labeled state. Thus, we normalized H3K9me1 2:0 to the sum of the H3K9me1 2:0, 2:1 and 2:2 peptides. This method of normalization avoids complications arising from variations in ionization efficiencies between peptides with different modification states.

A fundamental requirement of our turnover modeling is that the relative abundance of a post-translationally modified peptide, summed across all its isotopically labeled states, should remain at a steady state. Assuming that 95% (standard score = 1.96) of our observed data can be accounted by a measurement variability of 10%, a commonly cited upper bound, we checked whether the standard deviation of the relative abundances for a particular modified peptide across the time course remained within 0.10/1.96 = 0.051. For instance, if H3K79un, H3K79me1 and H3K79me2 fitted this criterion, we declared that all the modified forms of the peptide were at steady state relative to each other. For peptides that we never observed to be modified, such as the H4 24-35 peptide, we could not make this calculation because we normalized the peptide to itself and we assumed that these unmodified peptides were at steady state.

For each modified peptide, we then fitted a set of differential equations (see Additional file 1 and 3) to the relative abundance distributions for all labeled states using MATLAB (version 7.9.0; Mathworks, Natick, MA, USA) and iterated the program using 100-200 different initial parameter values to determine the set of optimized parameter values that results in the lowest objective or error value. For statistical comparison, we used either the Wilcoxon rank sum test or Kruskal-Wallis test (MATLAB version 7.9.0) for data points that were normally or non-normally distributed, respectively.

We adopted two independent and complementary approaches to assess the quality of the parameter estimates and the fit of the model to the data. Specifically, we examined the squared norm of the residual and computed confidence regions in parameter subspaces to elucidate parameter significance and independence.

In parameter estimation problems, confidence intervals (based on the Student *t*-test distribution) and/or elliptical confidence regions (based upon a Taylor series expansion around the parameter estimate) are often used to provide a range of values over which the parameter estimates are valid (that is, how much the estimated parameters are allowed to vary while still allowing the model to fit the data well). However, these aforementioned approaches, which are based on linear approximations [49], are only valid when the parameters vary symmetrically around the optimal estimates, and are not accurate for models with even moderate degrees of non-linearity [50]. To avoid the limitations associated with the inherent assumptions of these methods, we computed confidence regions around the parameter estimates using the *F*-test method (see Additional file 8) [51]. This *F*-test approach was applied to every pair of parameters to manually validate that the parameter estimates were indeed significant from zero and to visually assess any degree of nonlinearity in the confidence regions. The parameter estimates were found to be significant, and it was also observed that the confidence region was only slightly nonlinear (see Additional file 8).

## Competing interests

The authors declare that they have no competing interests.

## Authors' contributions

RSL conducted the labeling time course, prepared the histone samples and helped analyze the MS data. BMZ helped prepare the histone samples, analyzed the MS data, developed the turnover model and wrote the manuscript. PAD helped develop the turnover model and provided the quantitative estimates for the parameters and confidence regions. BAG conceived and designed the study. All authors read and approved the final manuscript.

## Supplementary Material

Additional file 1 **Figure S1: Tandem mass spectrometry (MS/MS) spectra of labeled peptides analyzed**. MS/MS spectra of partially and fully ^13^C_6_^15^N_2_-lysine labeled peptides, where the red and blue peaks correspond to the b and y ions annotated by Bioworks Browser. Click here for file

Additional file 2 **Table S1: List of all histone peptides analyzed: **All histone peptides quantified in our experiment are provided below, with their charge state, expected and observed mass to charge ratio (m/z). ac = Acetyl, me = methyl, ox = oxidation pr = propionyl, un = unmodified. *Charge state of the peptide is included; †unable to differentiate based on MS/MS. Click here for file

Additional file 3 **Figure S2: Detection of labeled isotopes**. Mass spectrometry spectrum of the unmodified H3 18-26 peptide after 1 day of ^13^C_6_^15^N_2_-lysine labeling, where the isotopic distributions for the peptide containing two ^12^C_6_^14^N_2_-lysines, one ^12^C_6_^14^N_2_-lysine and one ^13^C_6_^15^N_2_-lysine, and two ^13^C_6_^15^N_2_-lysines are detected. Click here for file

Additional file 4 **Figure S3: Model for histone turnover**. Set of differential equations that extrapolate histone turnover for a peptide containing one, two, three and four lysines. We found that at steady state, the rate of histone removal for a particular labeled state should equal the sum of all the rates of histone addition. This provides a constraint in the optimization procedure. Furthermore, because the rate of addition = rate of removal, k_-1 _becomes a measure of overall turnover for that peptide. For a peptide containing two lysines, we needed to include an additional factor of 2 for k_1 _because we were unable to differentiate which of the two lysines are isotopically labeled. For similar reasons, we modifid the differential equations for peptides containing more than one lysine. P_# _= relative abundance of peptide, where # indicates the number of labeled lysines. k_-1 _= Rate of histone removal. k_# _= Rate of histone addition, where # indicates the number of labeled lysines. Click here for file

Additional file 5 **Figure S4: Steady state assumption**. Steady state levels for histone post-translational modifications. Standard deviations (vertical bars) of the relative abundances for the H3 and H4 peptides across the labeling time course are shown relative to their respective means (black circles). Horizontal dashed lines denote a standard deviation of 0.051, the threshold at which 95% of the observed variability cannot be accounted by 5-10% of the instrument measurement variability. ac = Acetyl, me = methyl, un = unmodified. Click here for file

Additional file 6 **Figure S5: Comparison of turnover between histones**. (A) Relative distribution of the fully labeled H2A variant (black diamond), H3 (green diamond), H2b variant (pink diamond), H4 (dark blue diamond) and H1.4 (light blue diamond) core peptides during the time course. Lines represent fits based on the optimized kinetic parameters for the respective peptides. **(B) **Contour plots of the kinetic parameters for the respective core peptides (same color scheme as in (A)), where k_max _= k_1 _for H2A, H2B, H3 and H4, and k_2 _for H1.4. The limits of the contour plots are defined by *z*(k) × (1+2/5 × *F*_0.05_(2,5)) for all the histone peptides except the H1.4 peptide, where the limit is defined by *z*(k) × (1+3/11 × *F*_0.05_(3,11)) because of the additional lysine on the H1.4 peptide. Click here for file

Additional file 7 **Figure S6: Progressive modifications and turnover**. Turnover modeling (colored lines) of the relative distribution of the fully labeled unmodified (H3K18un, blue circle), monoacetylated (H3K18/K23ac1, green circle) and diacetylated (H3K18ac1K23ac1, red circle) H3 18-26 peptides across the labeling time course. Note the increasingly faster accumulation of the fully labeled peptides (increasingly leftward shift) as acetylation increases. Click here for file

Additional file 8 **Figure S7: Confidence regions for parameter estimates**. For each pair of estimated rate constants, we compared confidence regions using the *F*-test, which is presented below, where k is the vector containing the rate constants (that is, k_0_, k_1_,..., k_N_), $\hat{k}$is the vector of estimates for these rate constants as determined by solving the regression problem, z(k) is the value of the objective function for the regression problem (that is, the sum of the squared differences between the predicted and actual relative abundances), and $F_{p,n-p}^{\alpha }$ is the upper α critical value for the *F *distribution for *p *parameters and *n *data points. Thus, the corresponding confidence region for a given estimate $\hat{k}$ is the union of all k values that satisfy equation 1. Click here for file
