# Supplementary material for: Global turnover of histone post-translational modifications and variants in human cells
Source: Epigenetics Chromatin. 2010 Dec 6;3:22. doi: 10.1186/1756-8935-3-22 (PMC3004898; doi:10.1186/1756-8935-3-22)

H1.4 25 - 32

prKpr,me1:1STGAAKR H1.4K25me1:1

#8326-8326 RT:59.05-59.05 NL: 4.02E5

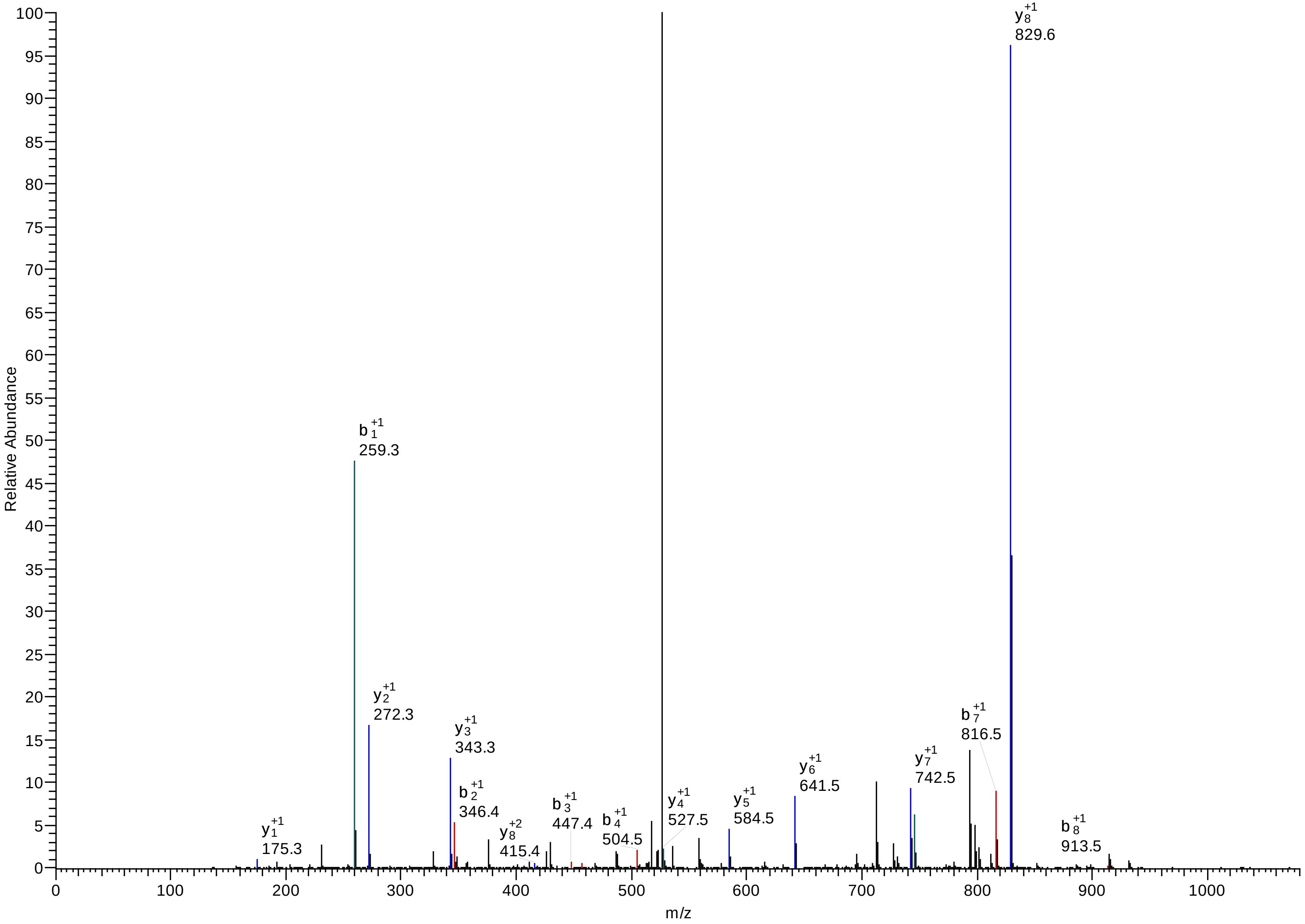

H3 3 - 8

TKpr,me1:1QTAR H3K4me1:1

#6739-6739 RT:47.95-47.95 NL: 2.09E4

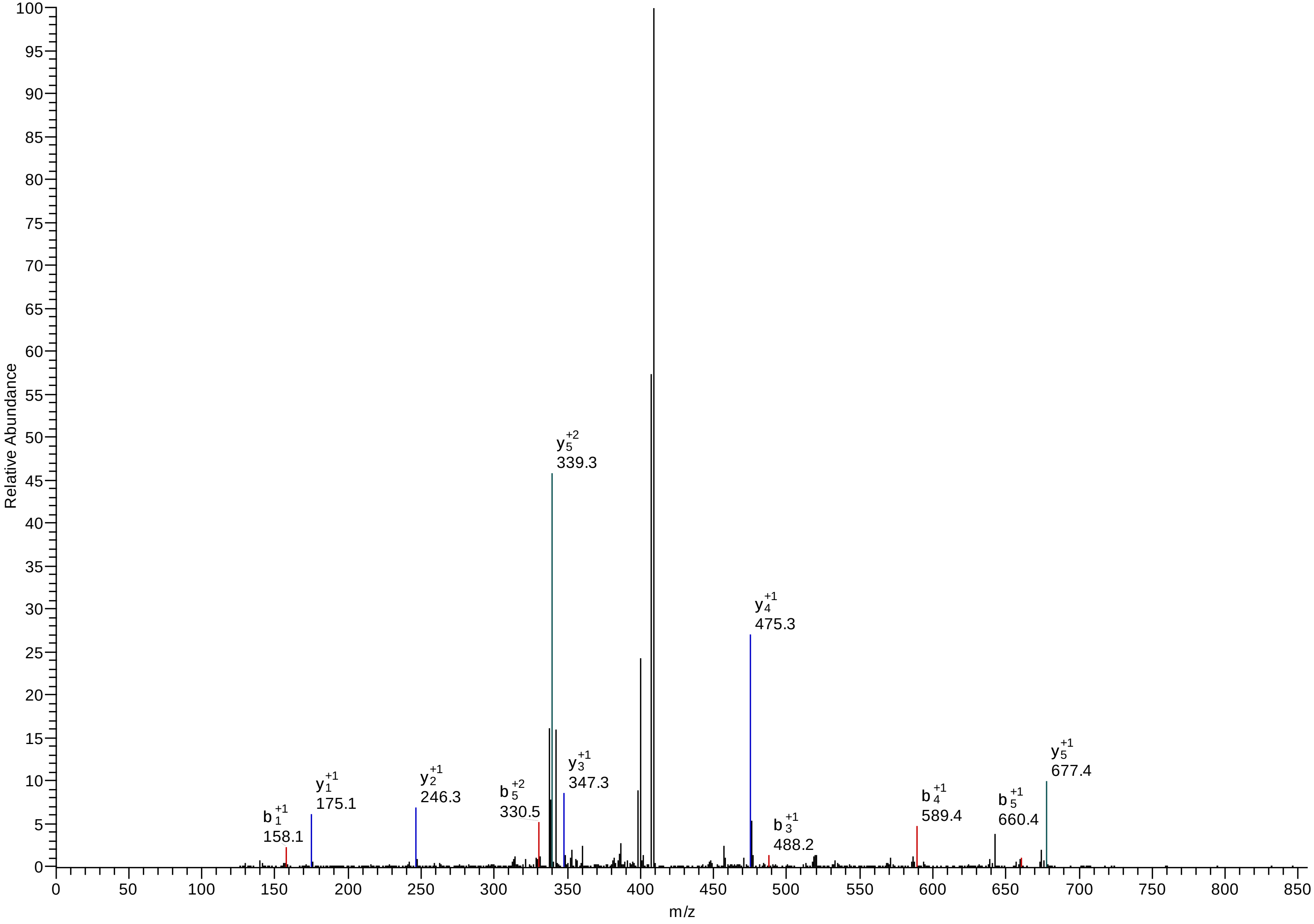

H3 9 - 17

prKpr,me1:1STGGKprAPR H3K9me1:1

#8710-8710 RT:59.52-59.52 NL: 4.34E5

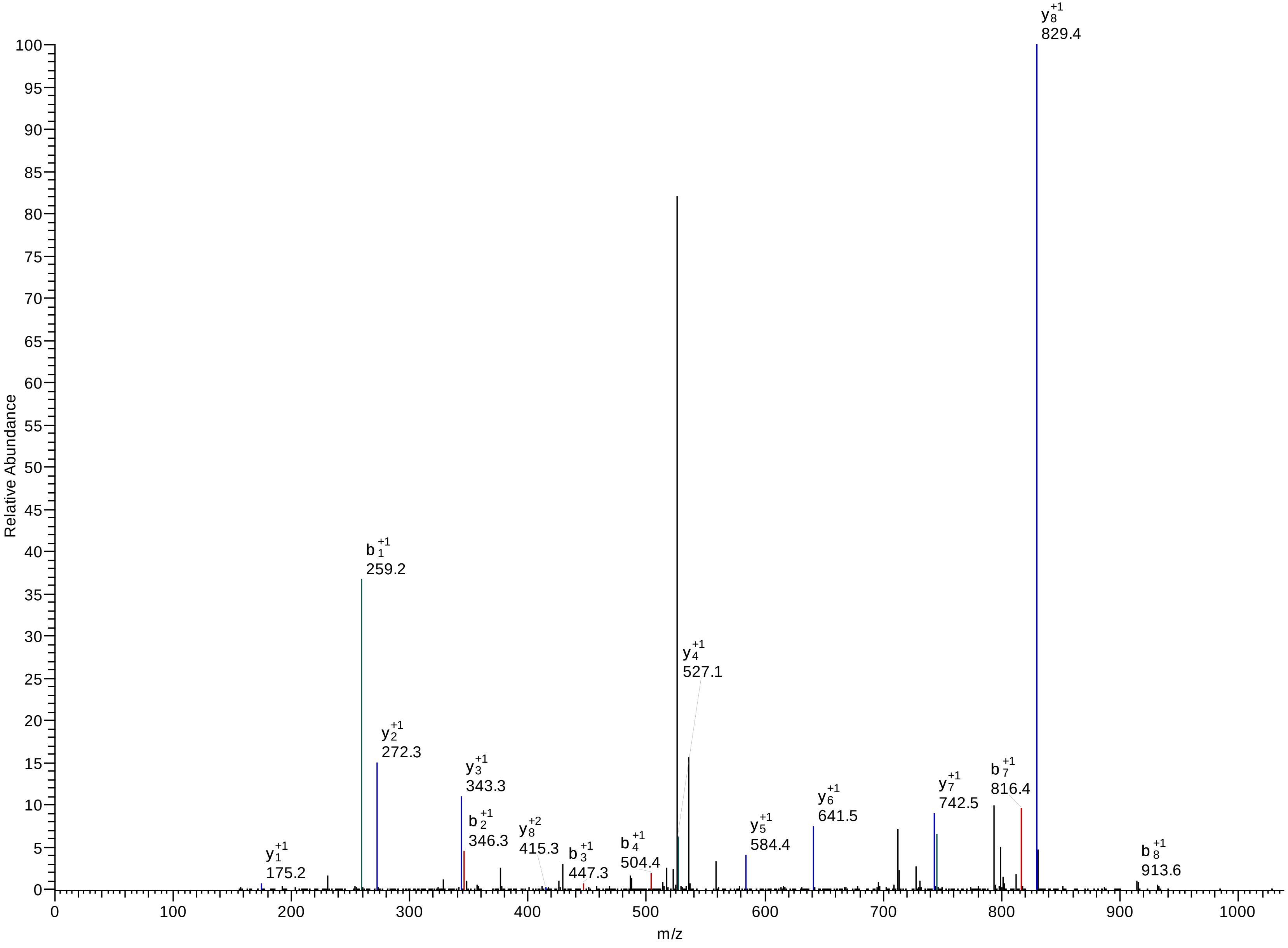

prKme2:1STGGKprAPR H3K9me2:1

#2906-2906 RT:25.20-25.20 NL: 5.79E2

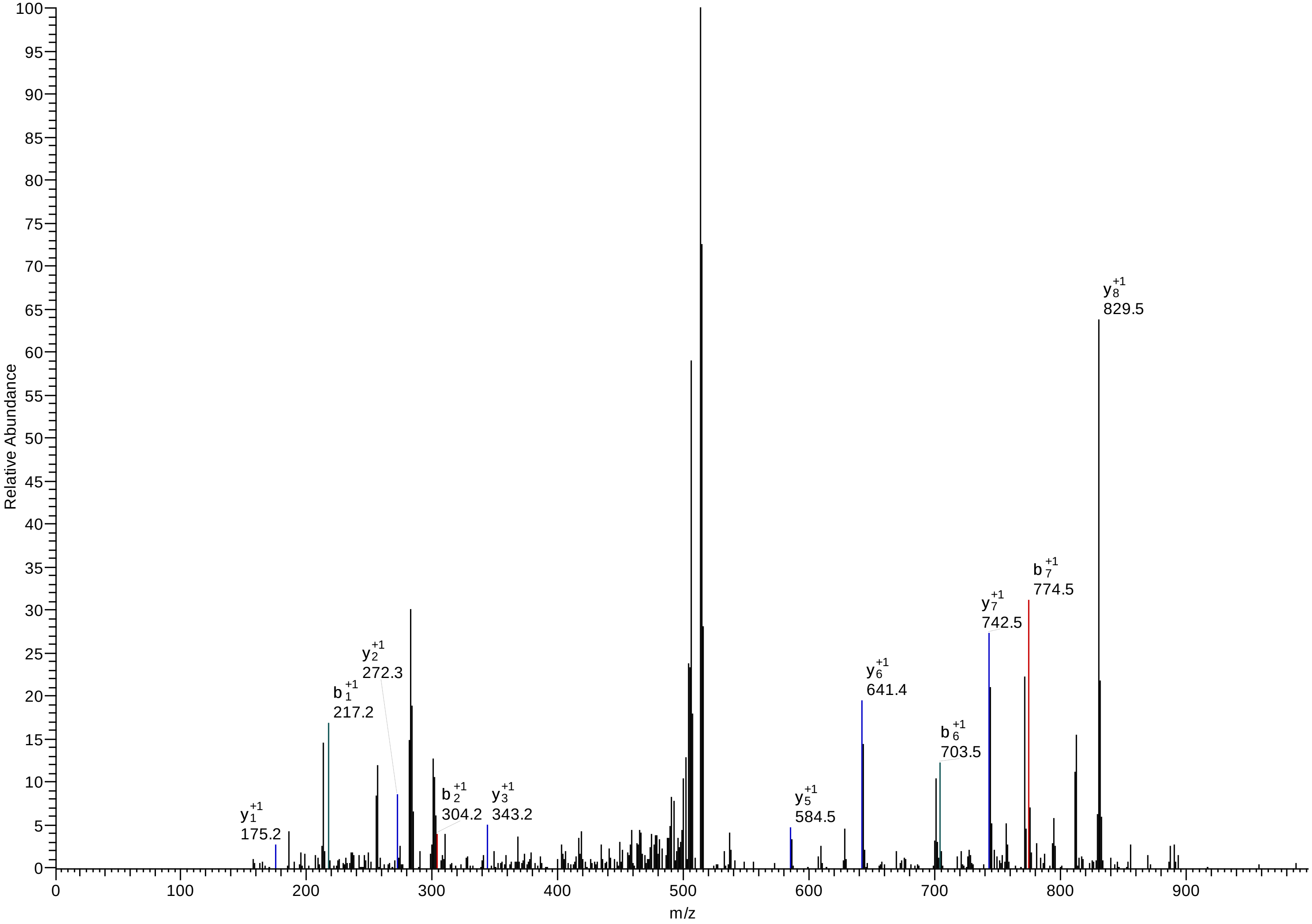

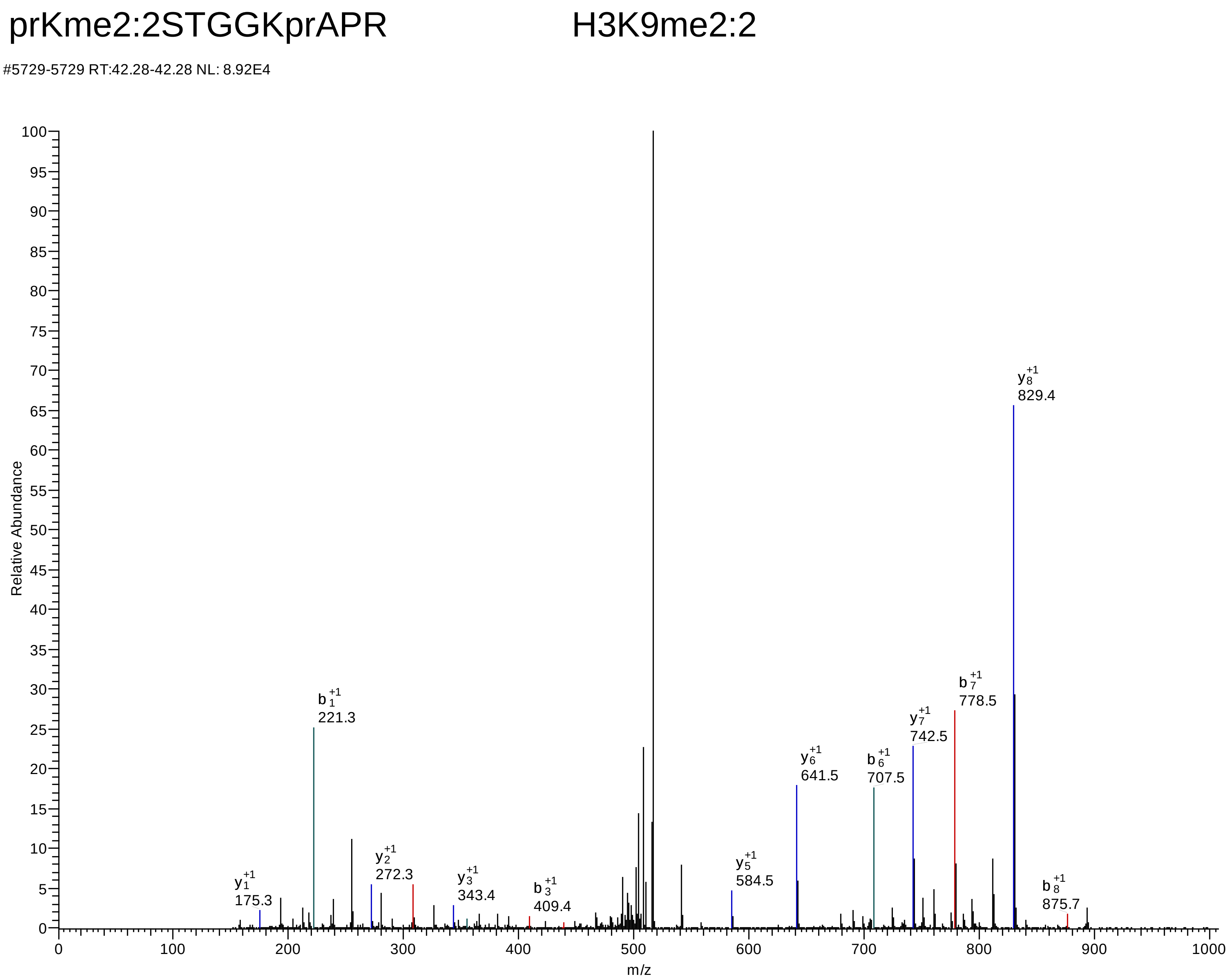
prKme2:2STGGKprAPR
H3K9me2:2
#5729-5729 RT:42.28-42.28 NL: 8.92E4
Relative Abundance
m/z
y1 +1 175.3
b1 +1 221.3
y2 +1 272.3
y3 +1 343.4
b3 +1 409.4
y5 +1 584.5
y6 +1 641.5
b6 +1 707.5
y7 +1 742.5
b7 +1 778.5
y8 +1 829.4
b8 +1 875.7

prrKme3:1STGGKprAPR H3K9me3:1

#3134-3134 RT:26.63-26.63 NL: 1.04E2

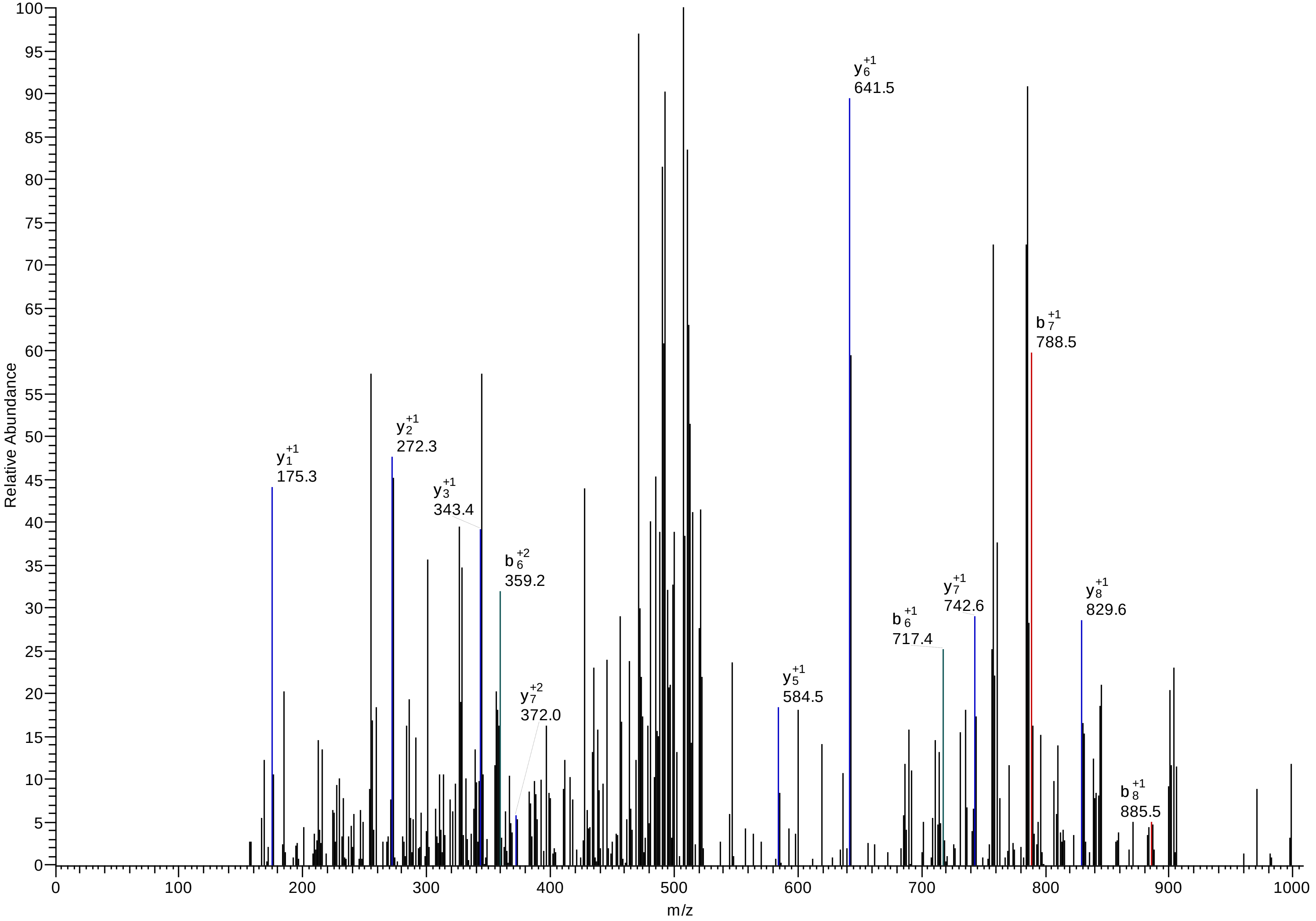

prKme3:2STGGKprAPR H3K9me3:2

#5365-5365 RT:40.01-40.01 NL: 1.46E3

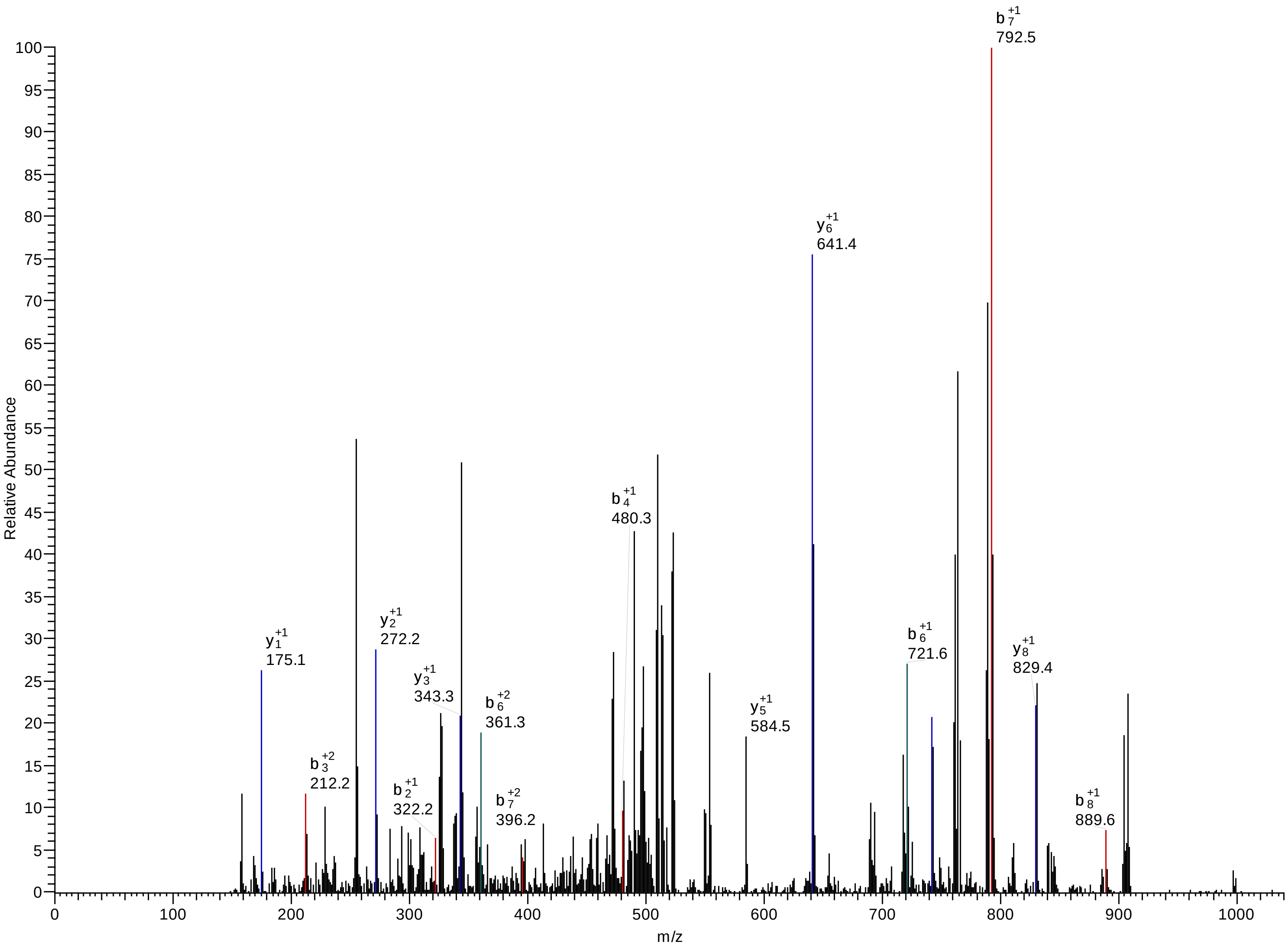

prKme3:3STGGKprAPR   H3K9me3:3

#5660-5660 RT:41.89-41.89 NL: 2.46E3

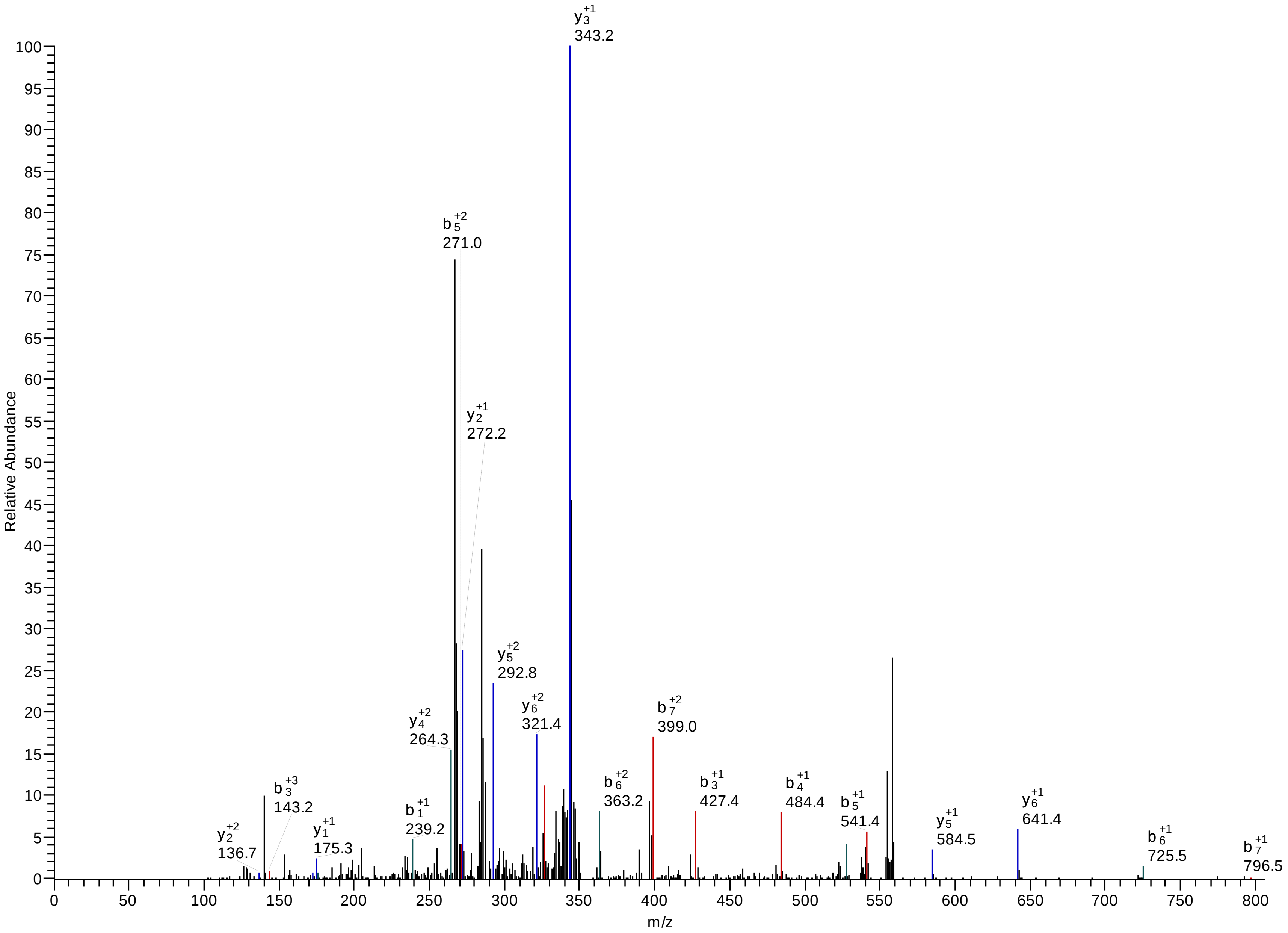

prKpr,me1:1STGGKacAPR H3K9me1:1K14ac1

#8209-8209 RT:56.59-56.59 NL: 4.27E3

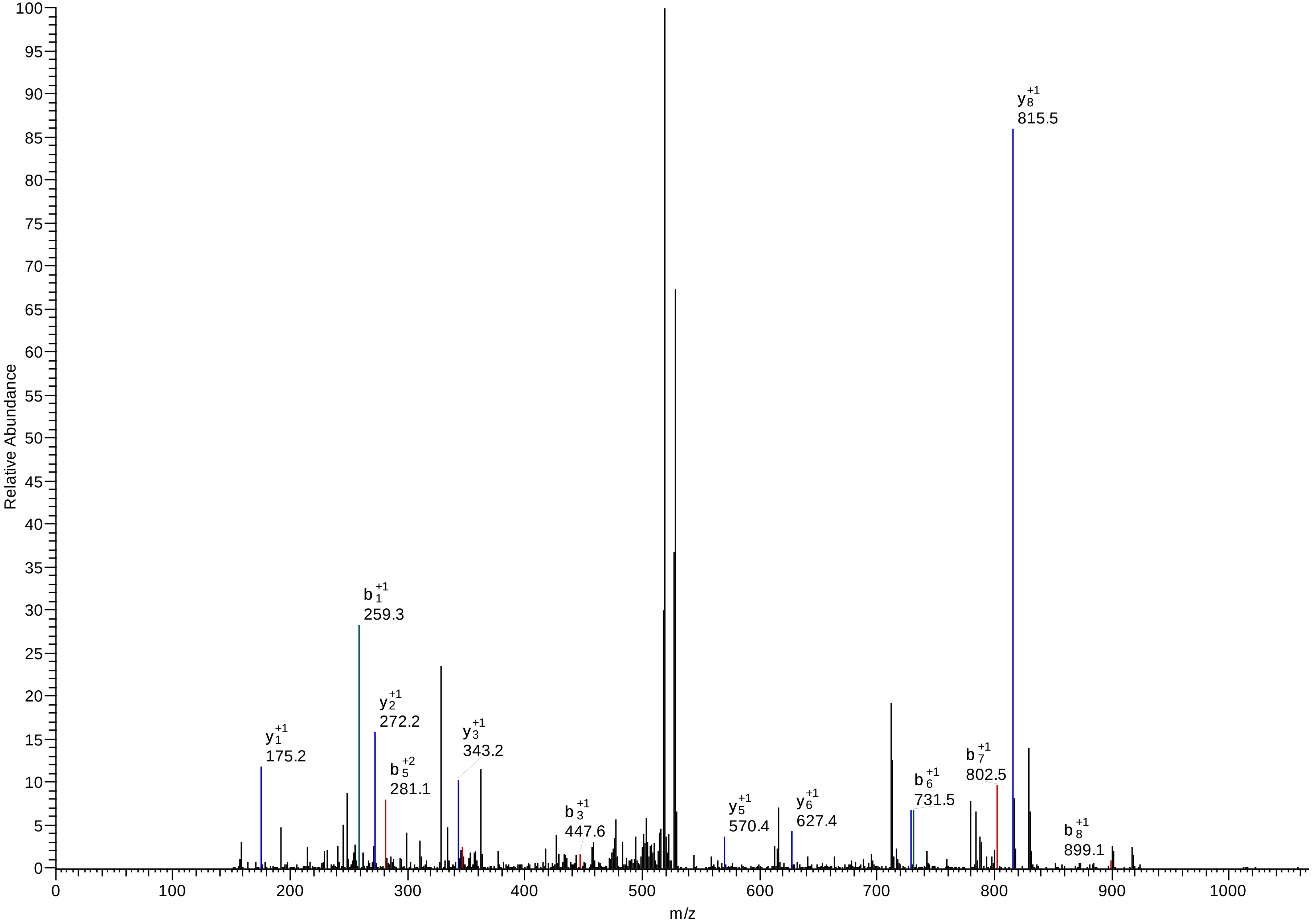

prKme2:1STGGKacAPR H3K9me2:1K14ac1

#2124-2124 RT:20.78-20.78 NL: 3.31E2

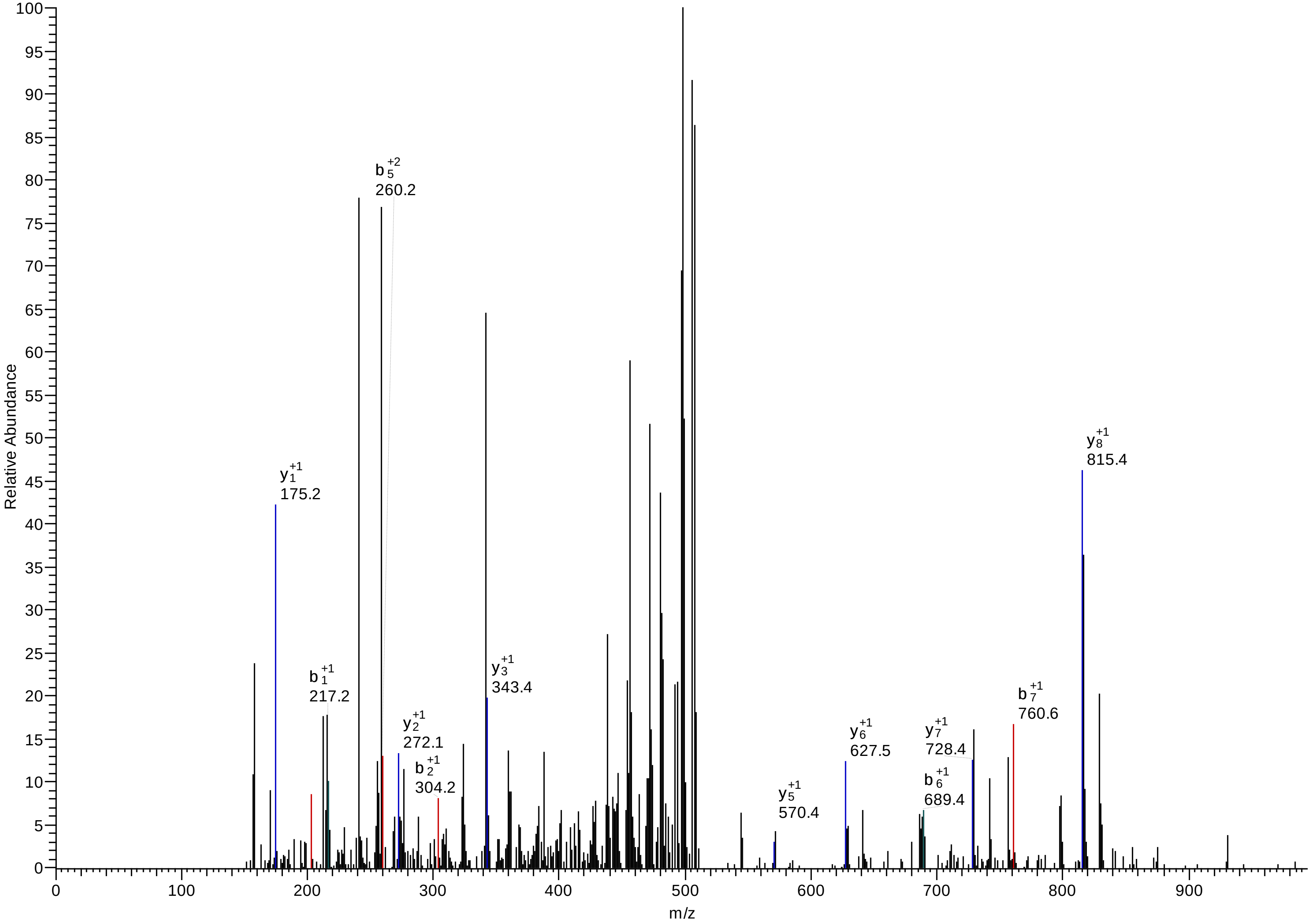

prKme2:2STGGKacAPR H3K9me2:2K14ac1

#4802-4802 RT:36.99-36.99 NL: 8.66E3

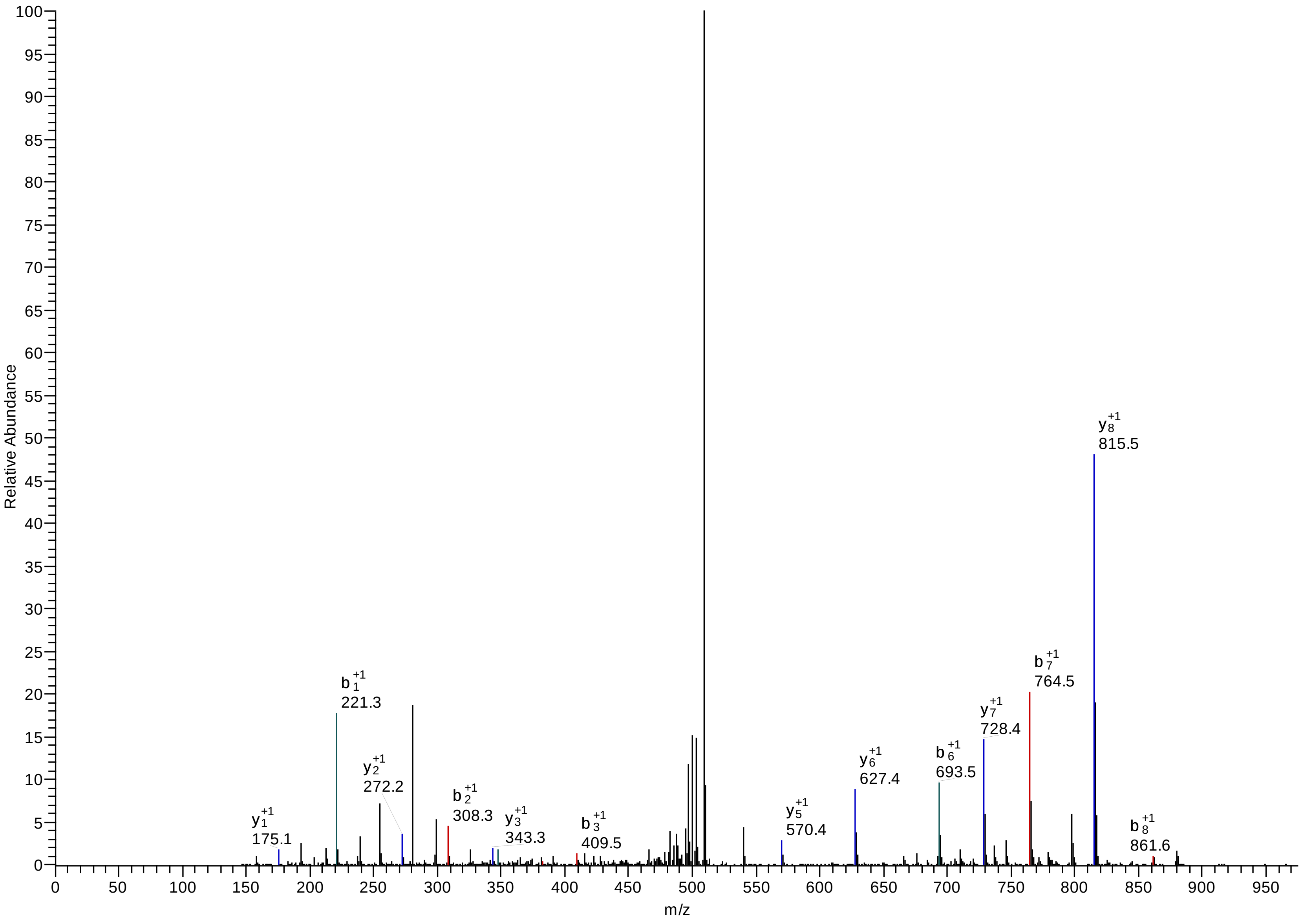

H3 18- 26

prKpr,me1:1QLATKprAAR H3K18me1:1

#12492-12492 RT:81.61-81.61 NL: 1.06E4

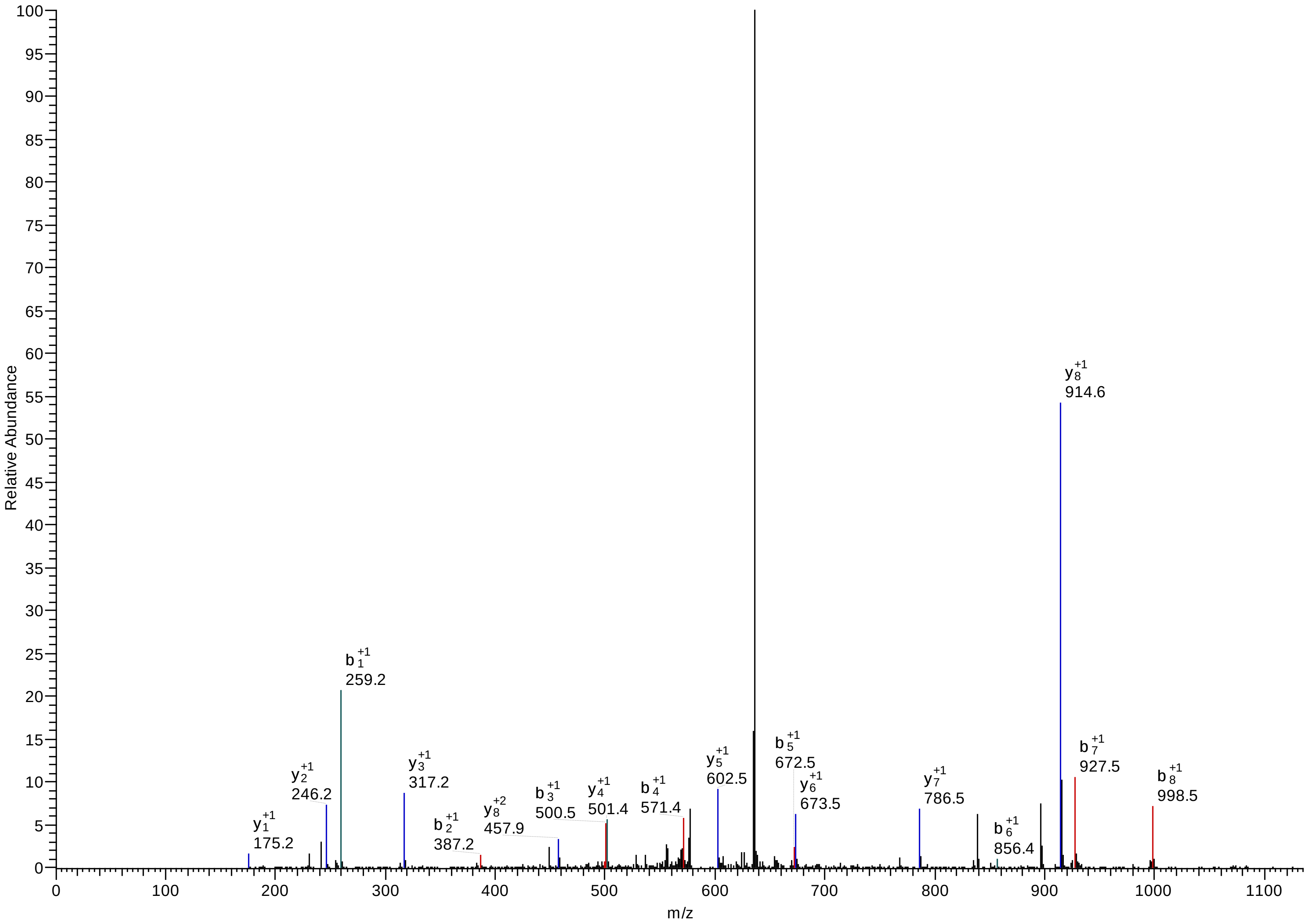

H3 27-40

prKprSAPATGGVKpr,me1:1KprPHR H3K36me1:1

#10093-10093 RT:67.69-67.69 NL: 7.09E3

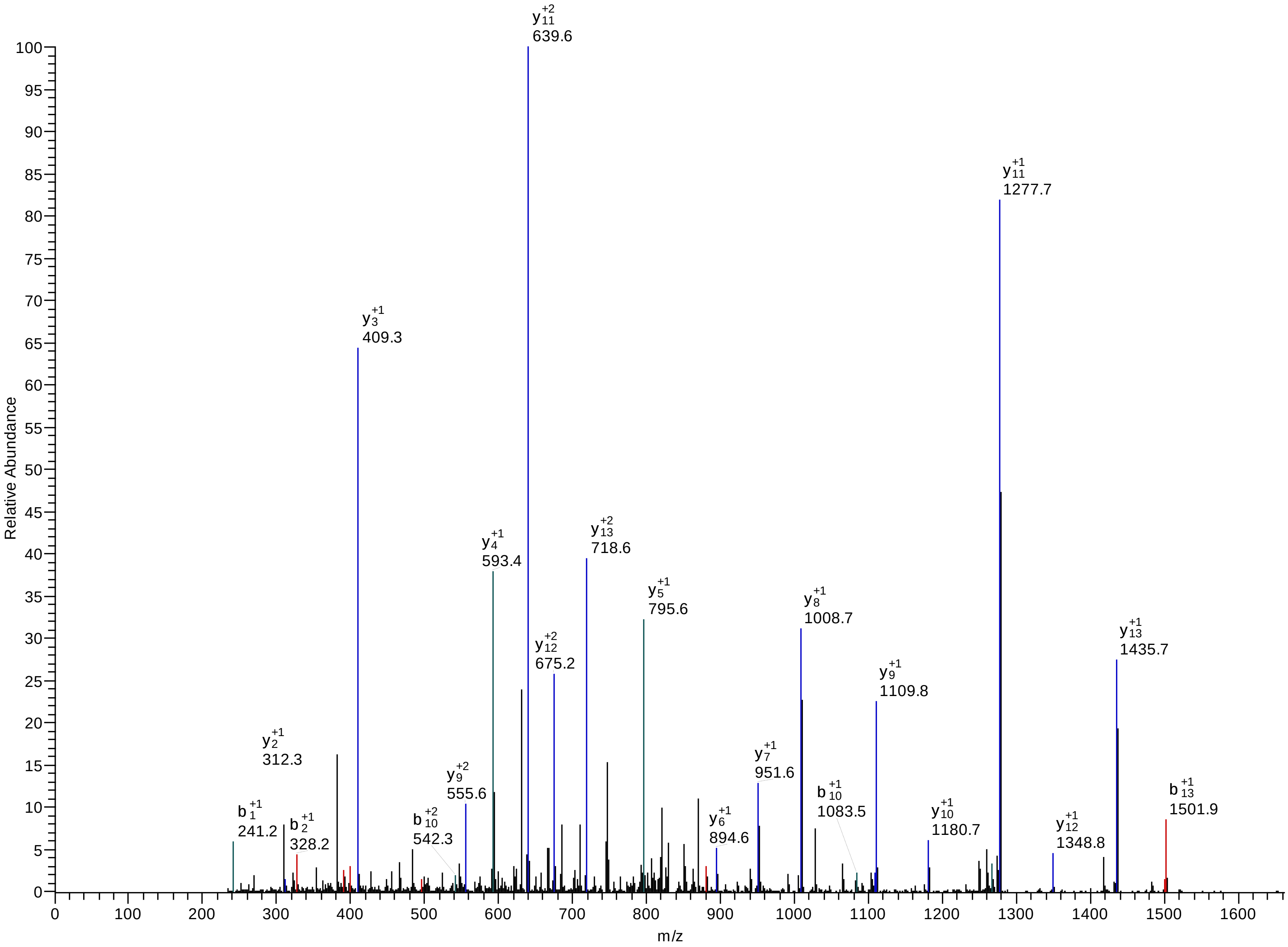

prKpr,me1:1SAPATGGVKprKprPHR H3K27me1:1

#10688-10688 RT:71.22-71.22 NL: 1.28E4

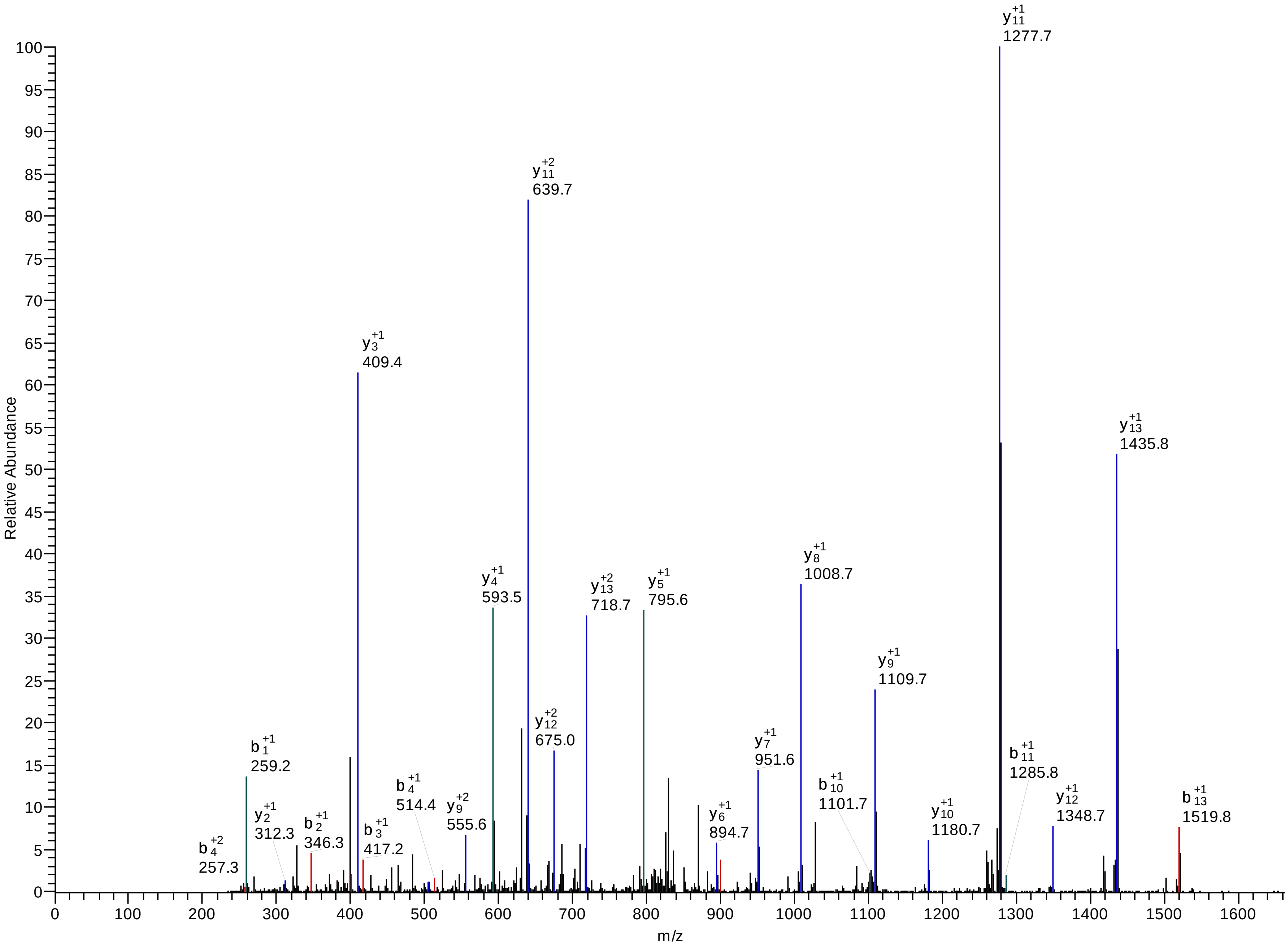

prKme2:1SAPATGGVKprKprPHR   H3K27me2:1

#7752-7752 RT:53.83-53.83 NL: 1.43E4

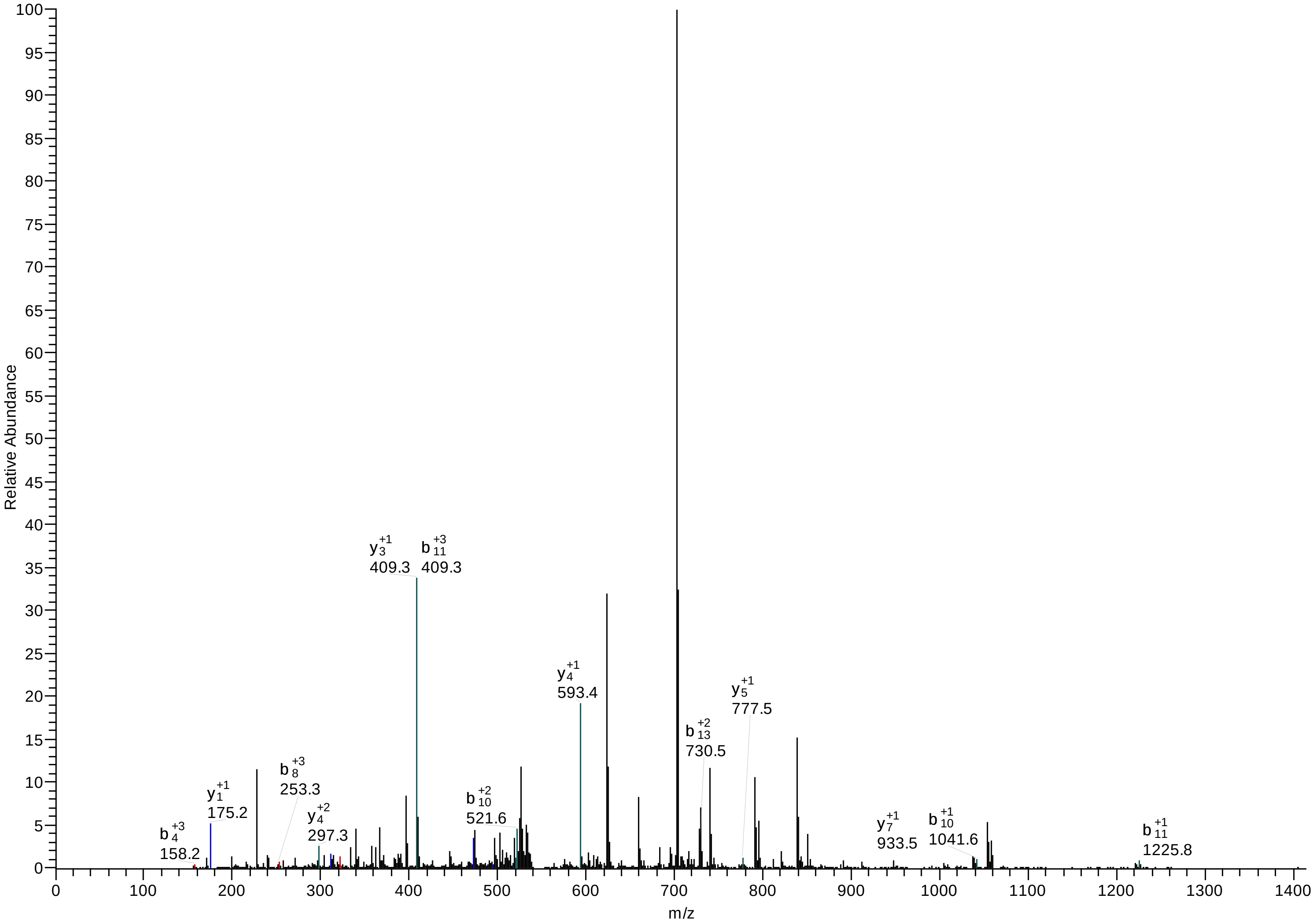

prKme2:2SAPATGGVKprKprPHR H3K27me2:2

#8320-8320 RT:57.17-57.17 NL: 4.52E3

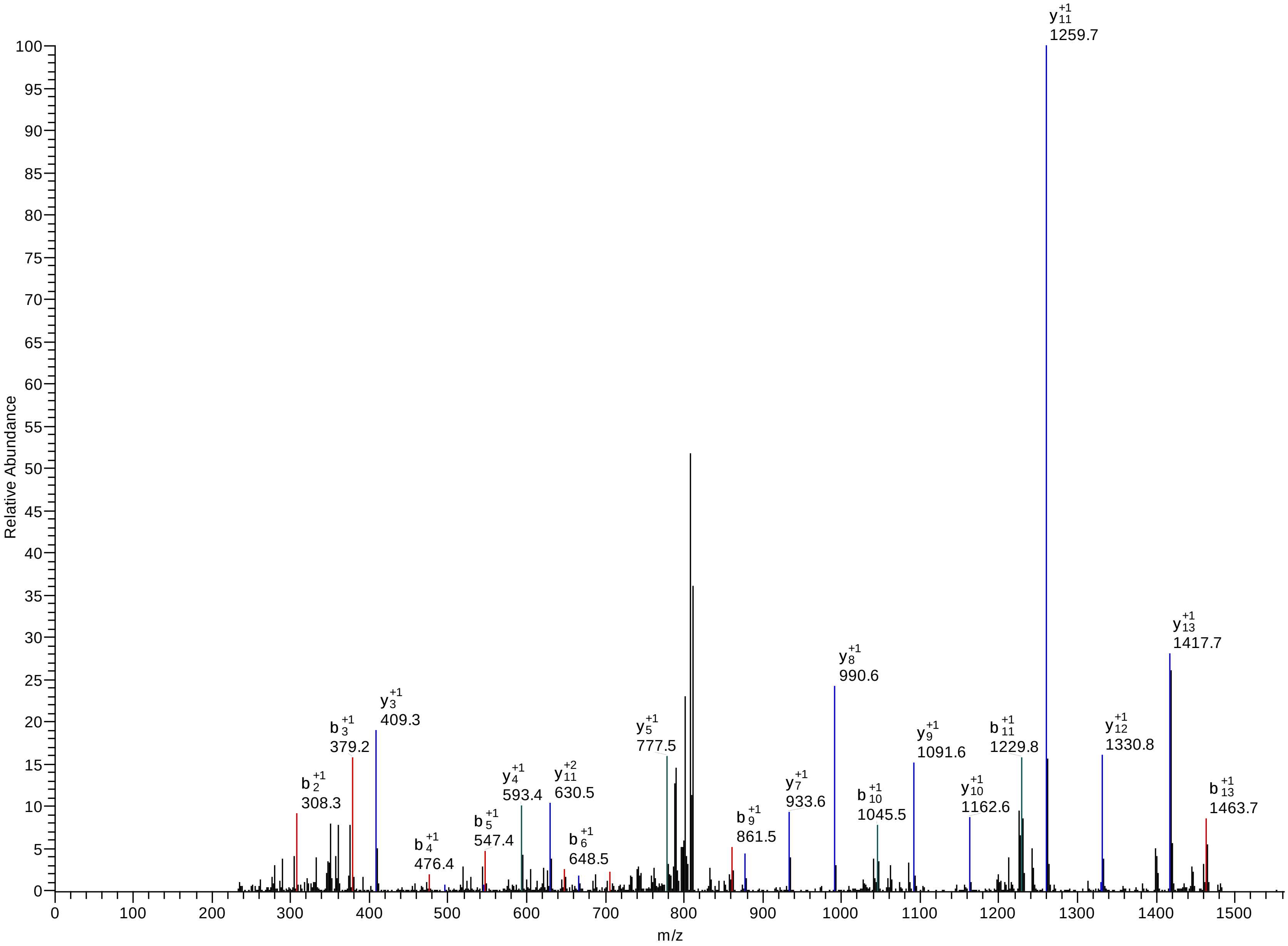

prKprSAPATGGVKme2:1KprPHR   H3K36me2:1

#8415-8415 RT:57.75-57.75 NL: 2.12E4

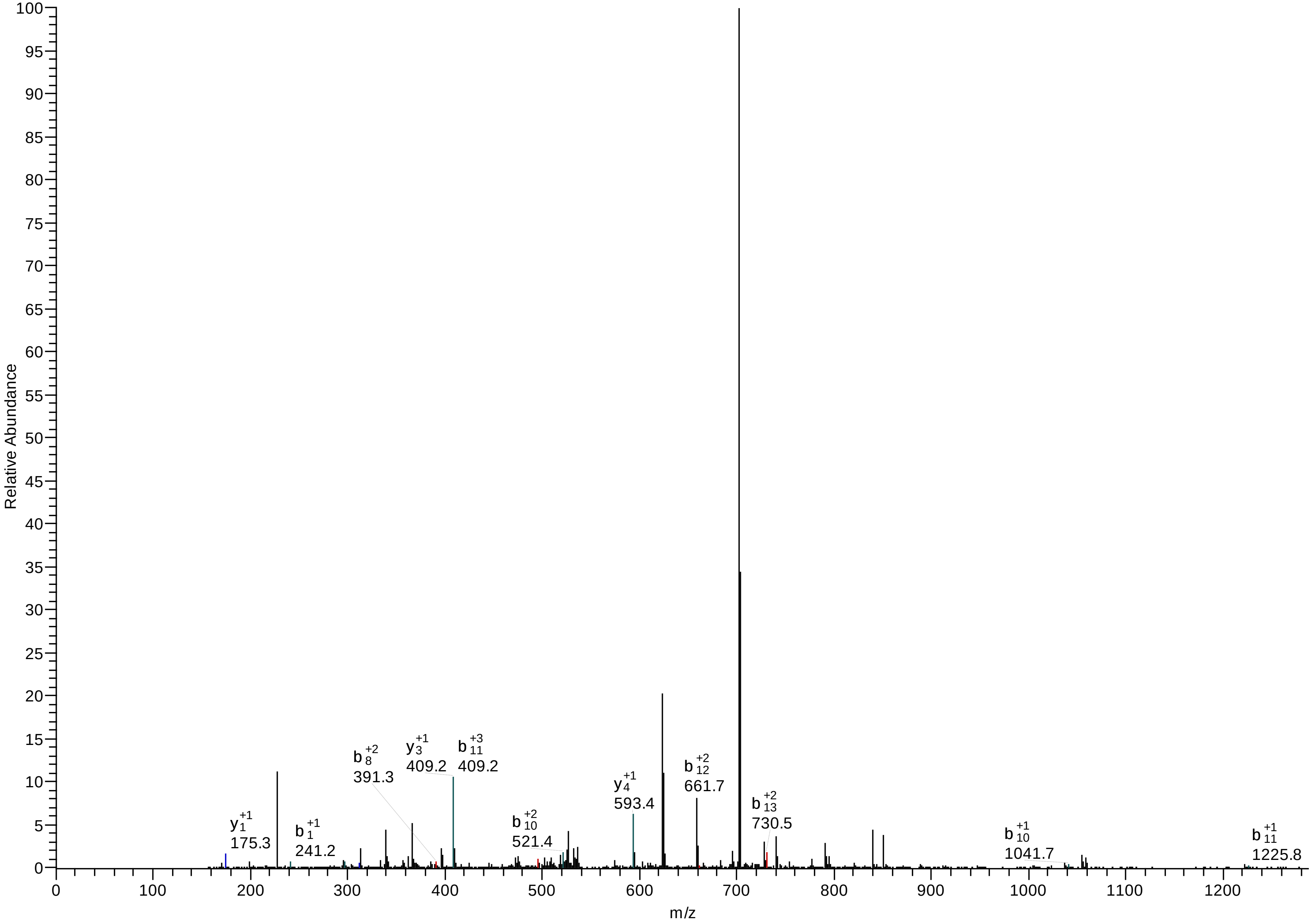

prKprSAPATGGVKme2:2KprPHR H3K36me2:2

#8054-8054 RT:55.62-55.62 NL: 7.57E3

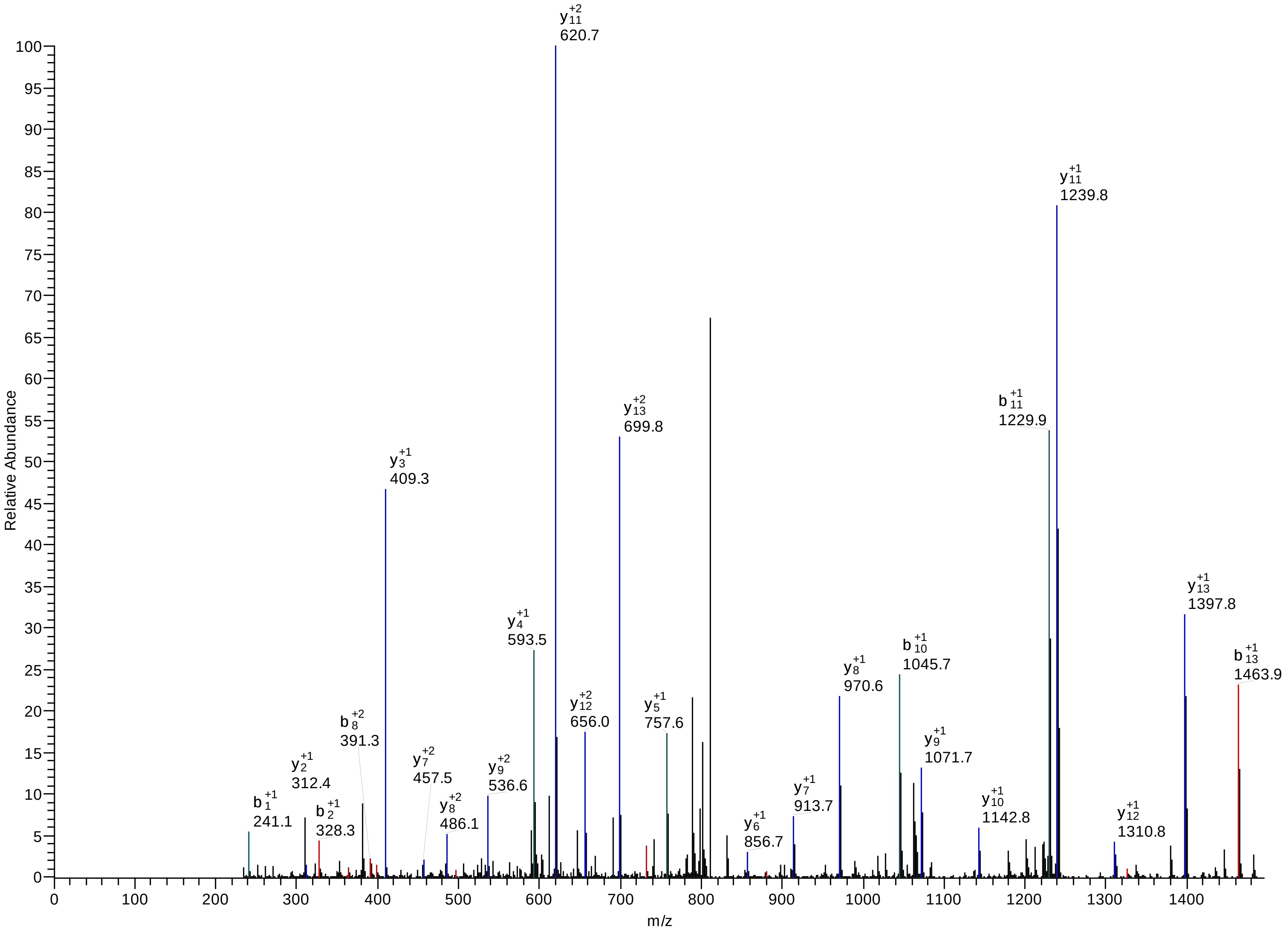

prKpr,me1:1SAPATGGVKpr,me1:0KprPHR   H3K27me1:1K36me1:0

#9492-9492 RT:64.14-64.14 NL: 2.61E3

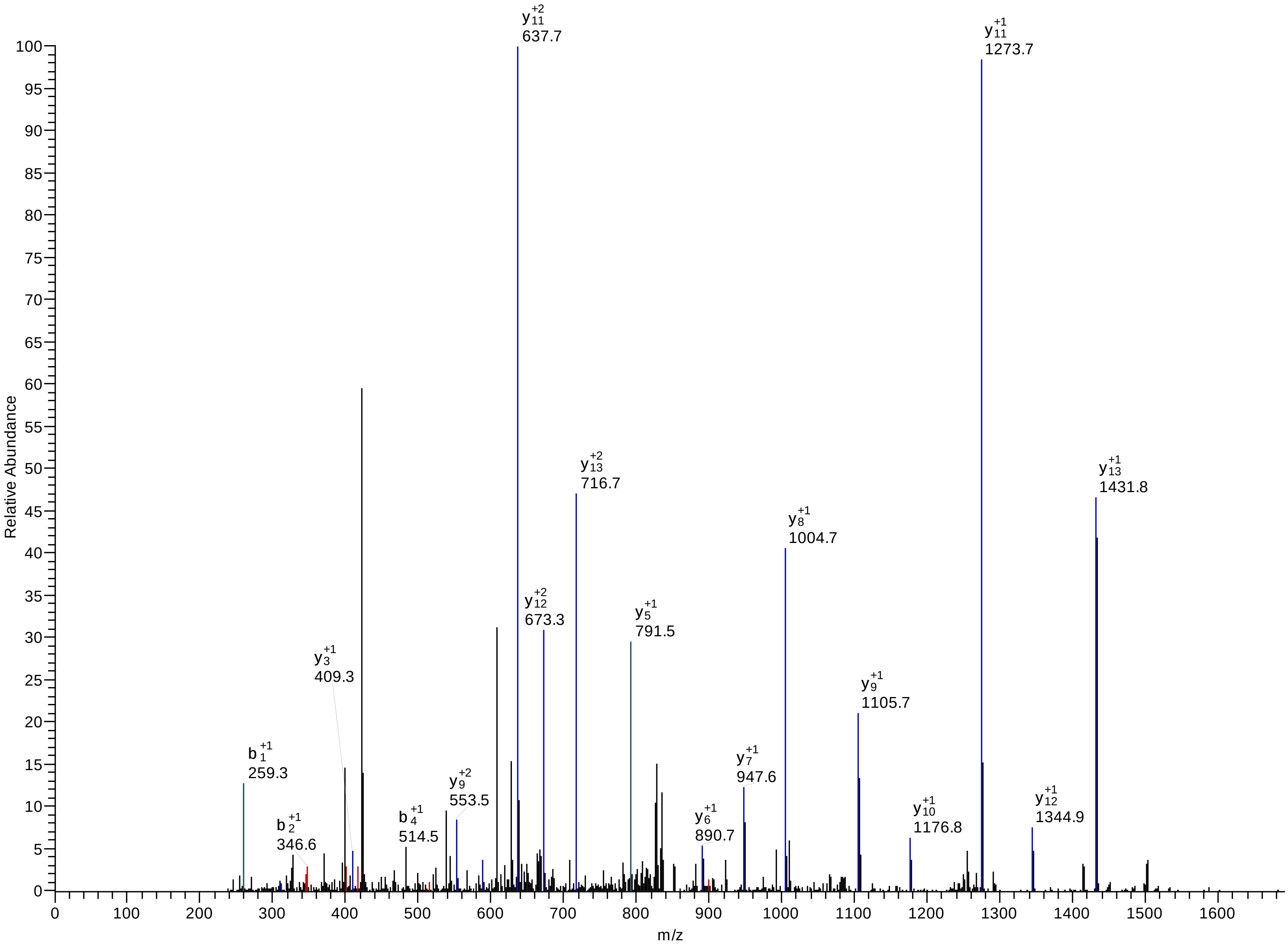

prKpr,me1:1SAPATGGVKpr,me1:1KprPHR

#10074-10074 RT:67.58-67.58 NL: 7.92E4

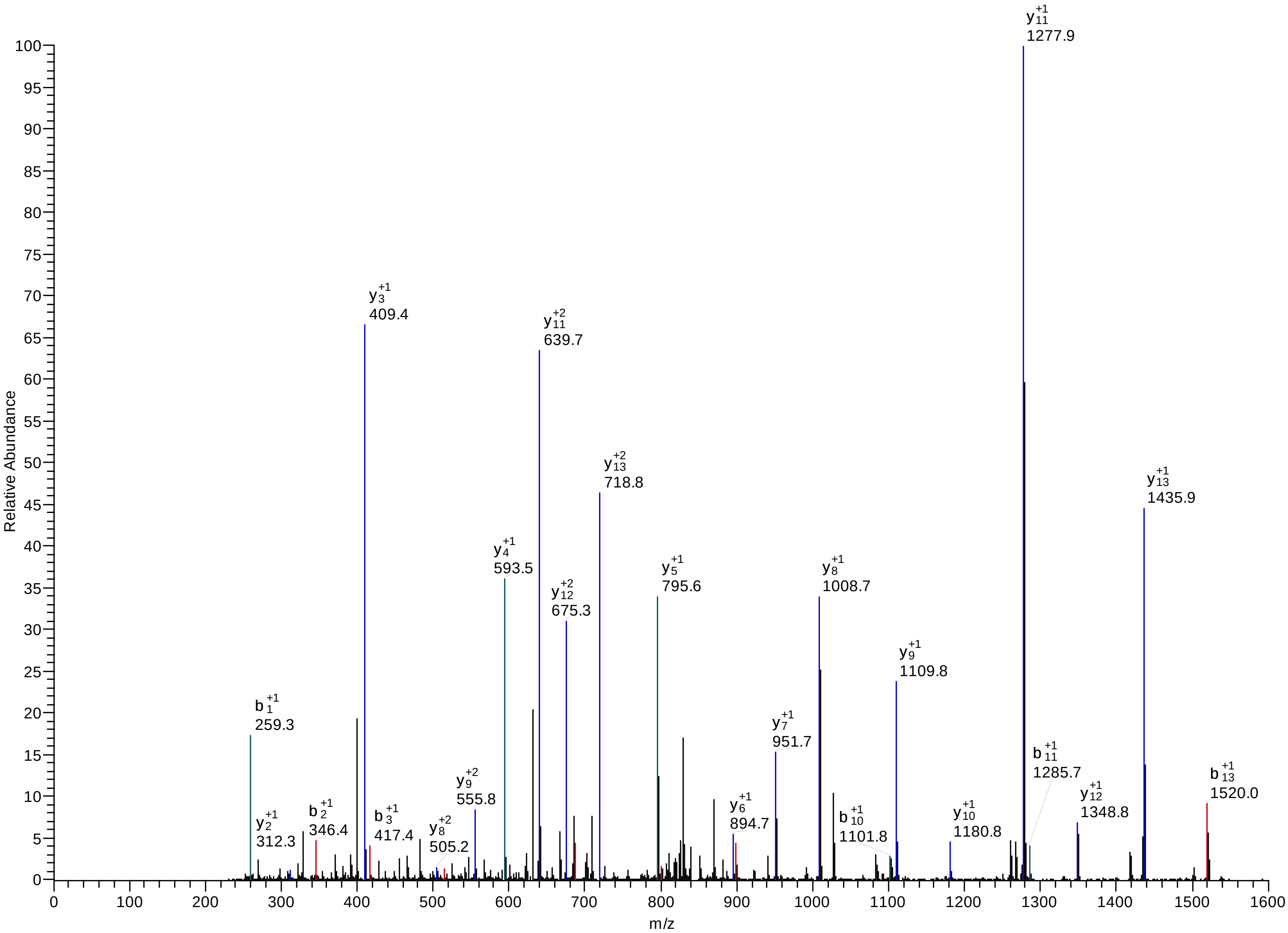

prKme3:1SAPATGGVKprKprPHR H3K27me3:1

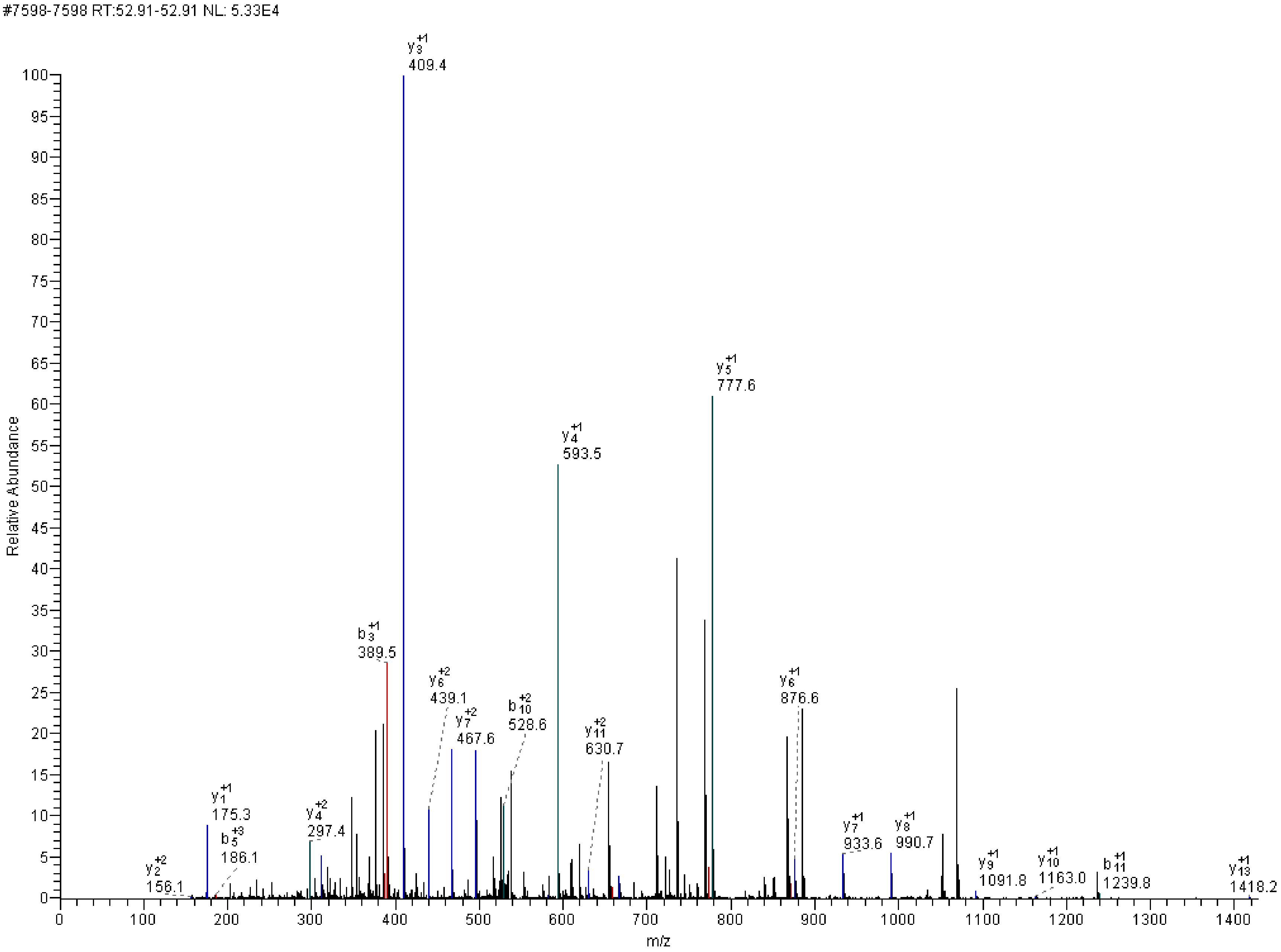

prKme2:0SAPATGGVKpr,me1:1KprPHR H3K27me2:0K36me1:1

#8620-8620 RT:58.97-58.97 NL: 6.47E3

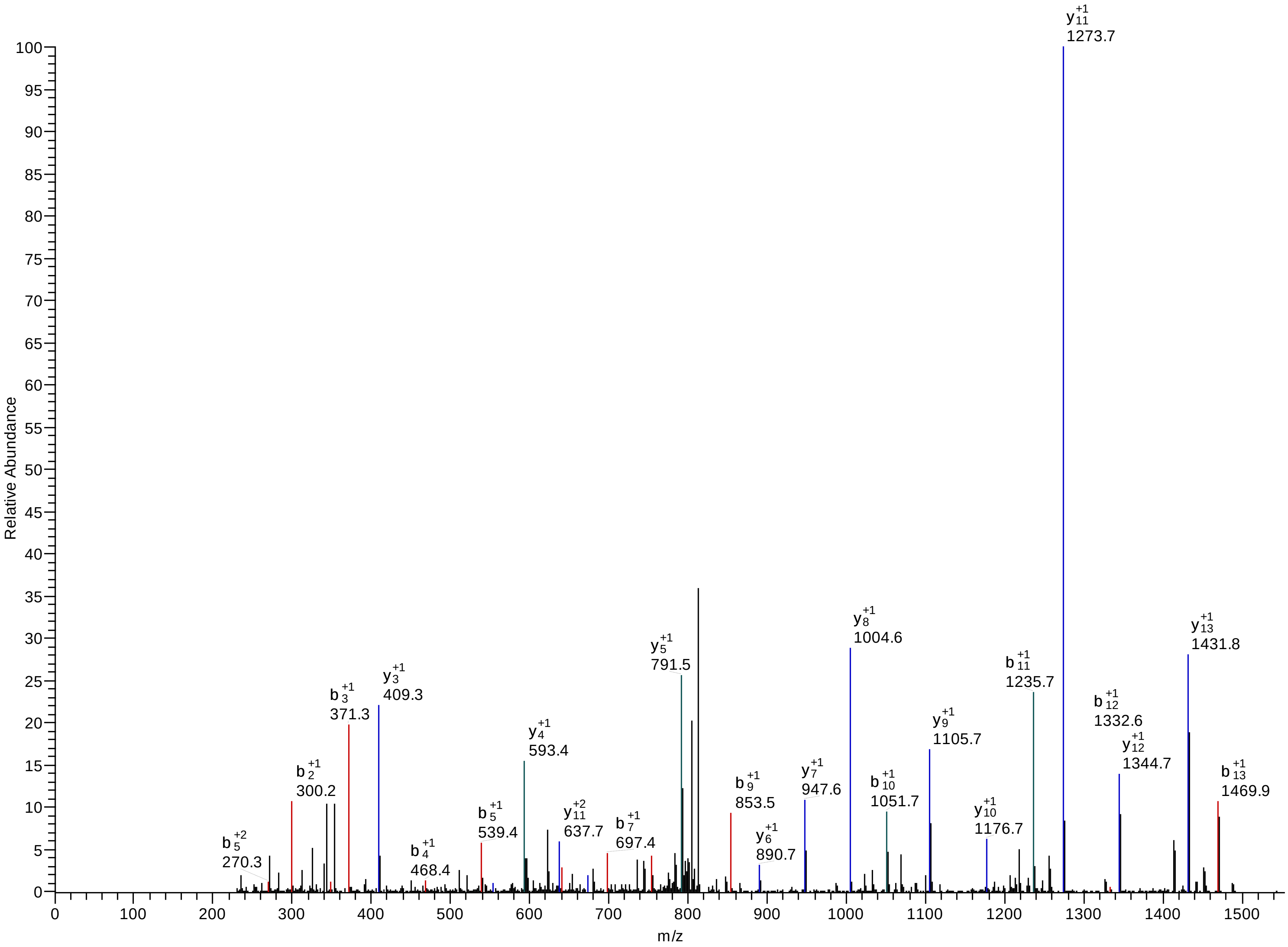

prKpr,me1:1SAPATGGVKme2:0KprPHR H3K27me1:1K36me2:0

#9190-9190 RT:62.34-62.34 NL: 3.71E3

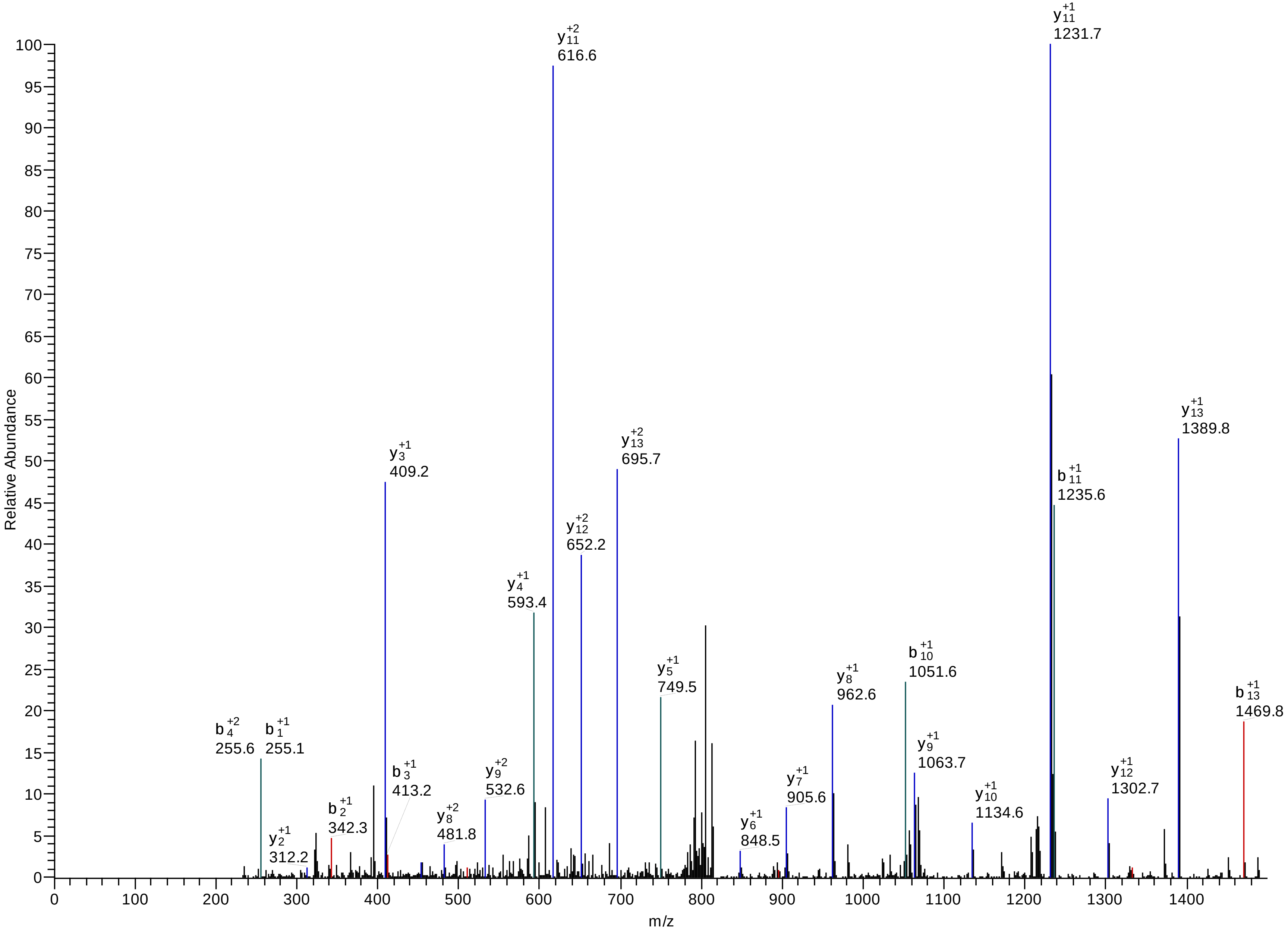

prKme3:2SAPATGGVKprKprPHR H3K27me3:2

#7502-7502 RT:52.36-52.36 NL: 4.52E4

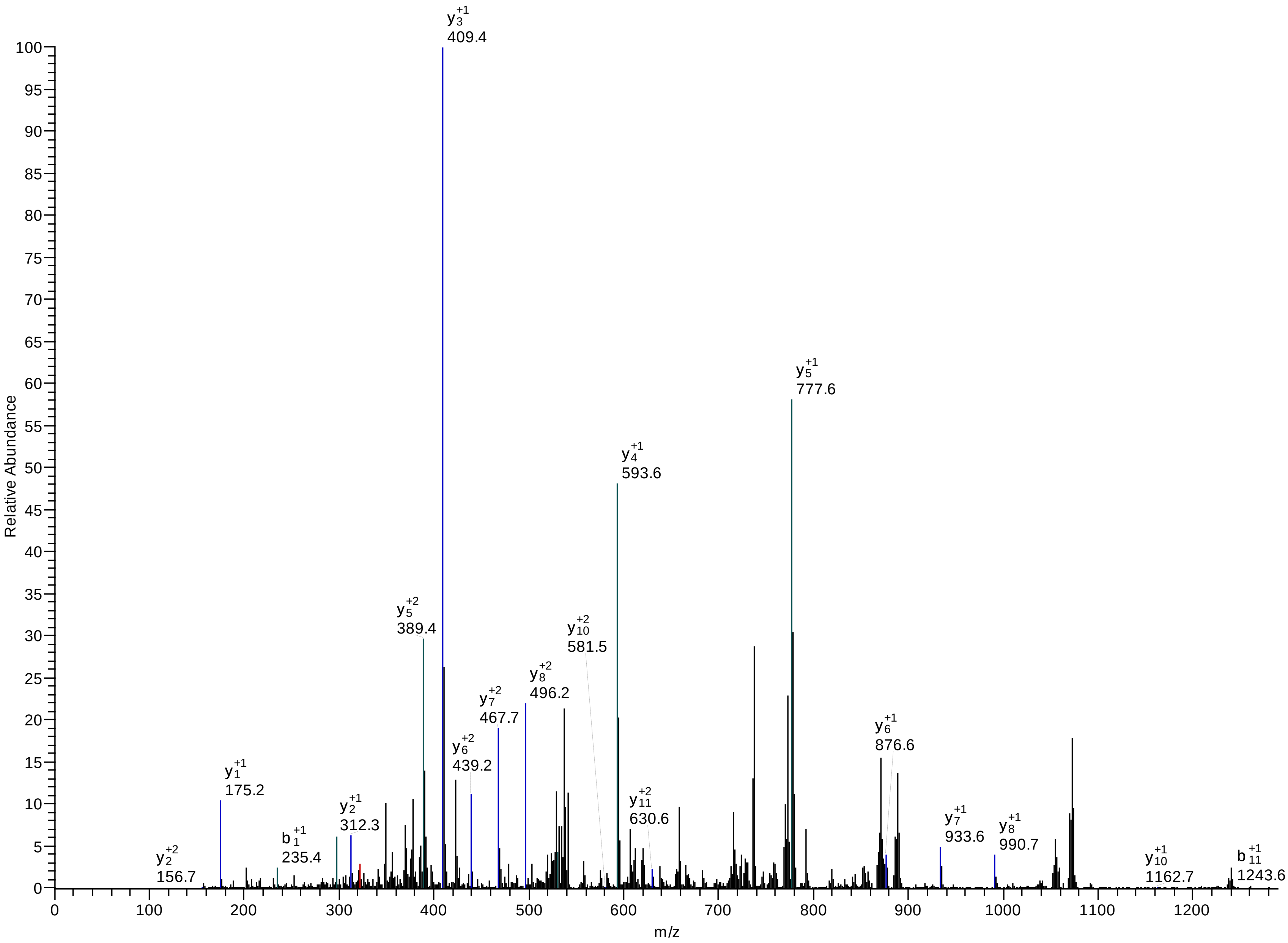

prKme2:1SAPATGGVKpr,me1:1KprPHR   H3K27me2:1K36me1:1

#7975-7975 RT:55.15-55.15 NL: 6.11E3

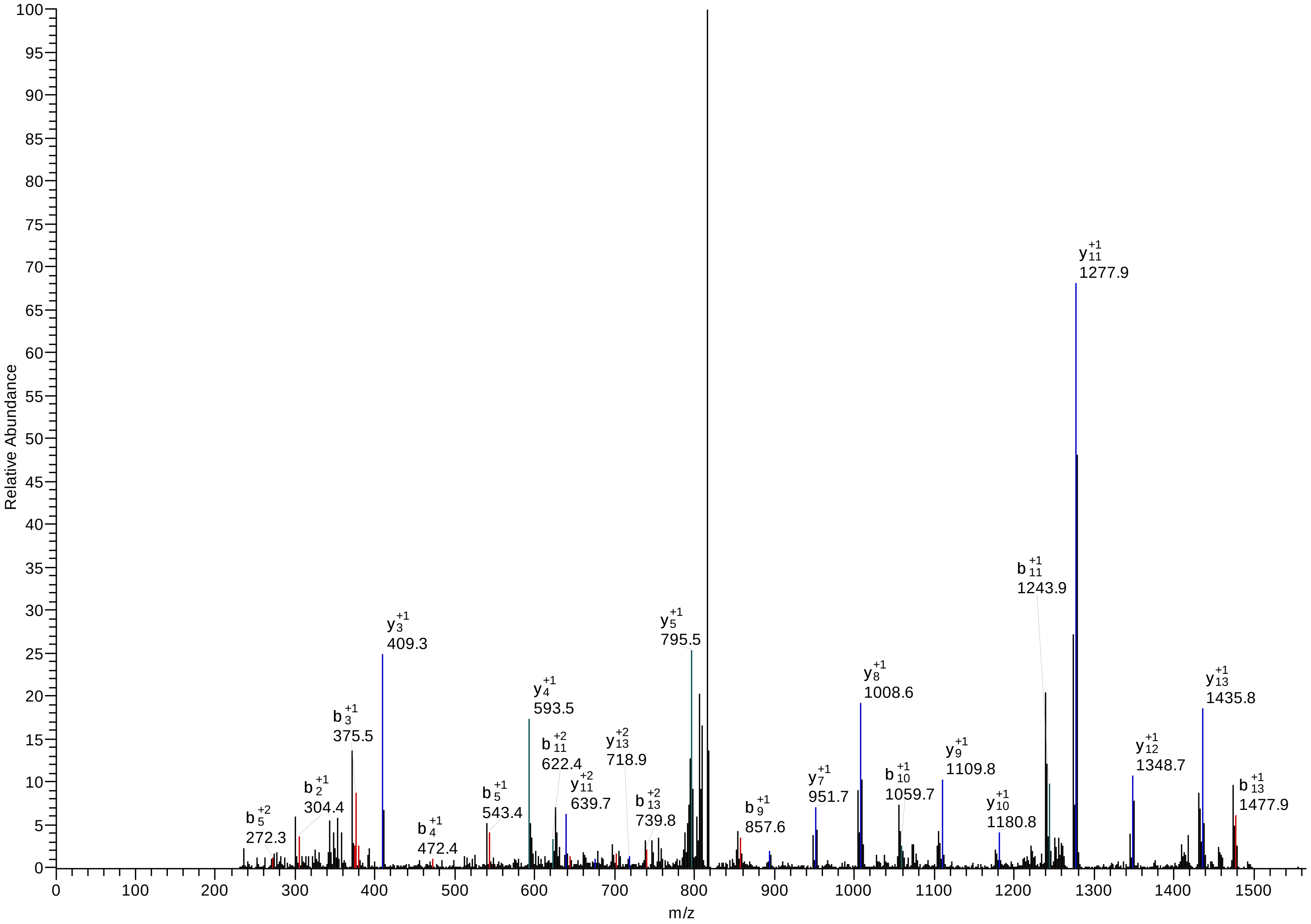

prKpr,me1:1SAPATGGVKme2:1KprPHR H3K27me1:1K36me2:1

#8560-8560 RT:58.62-58.62 NL: 3.37E3

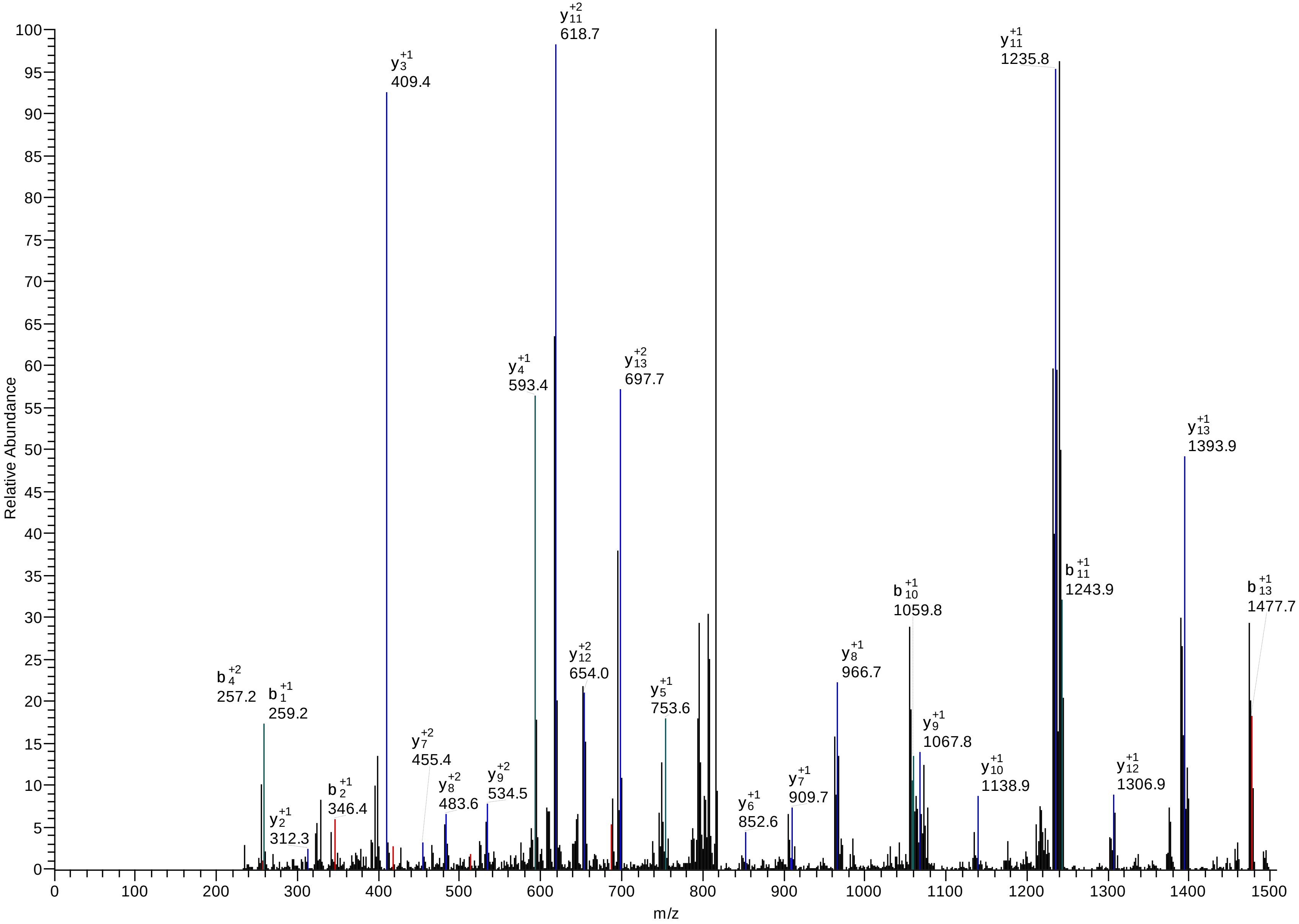

prKme3:3SAPATGGVKprKprPHR H3K27me3:3

#7547-7547 RT:52.62-52.62 NL: 1.03E3

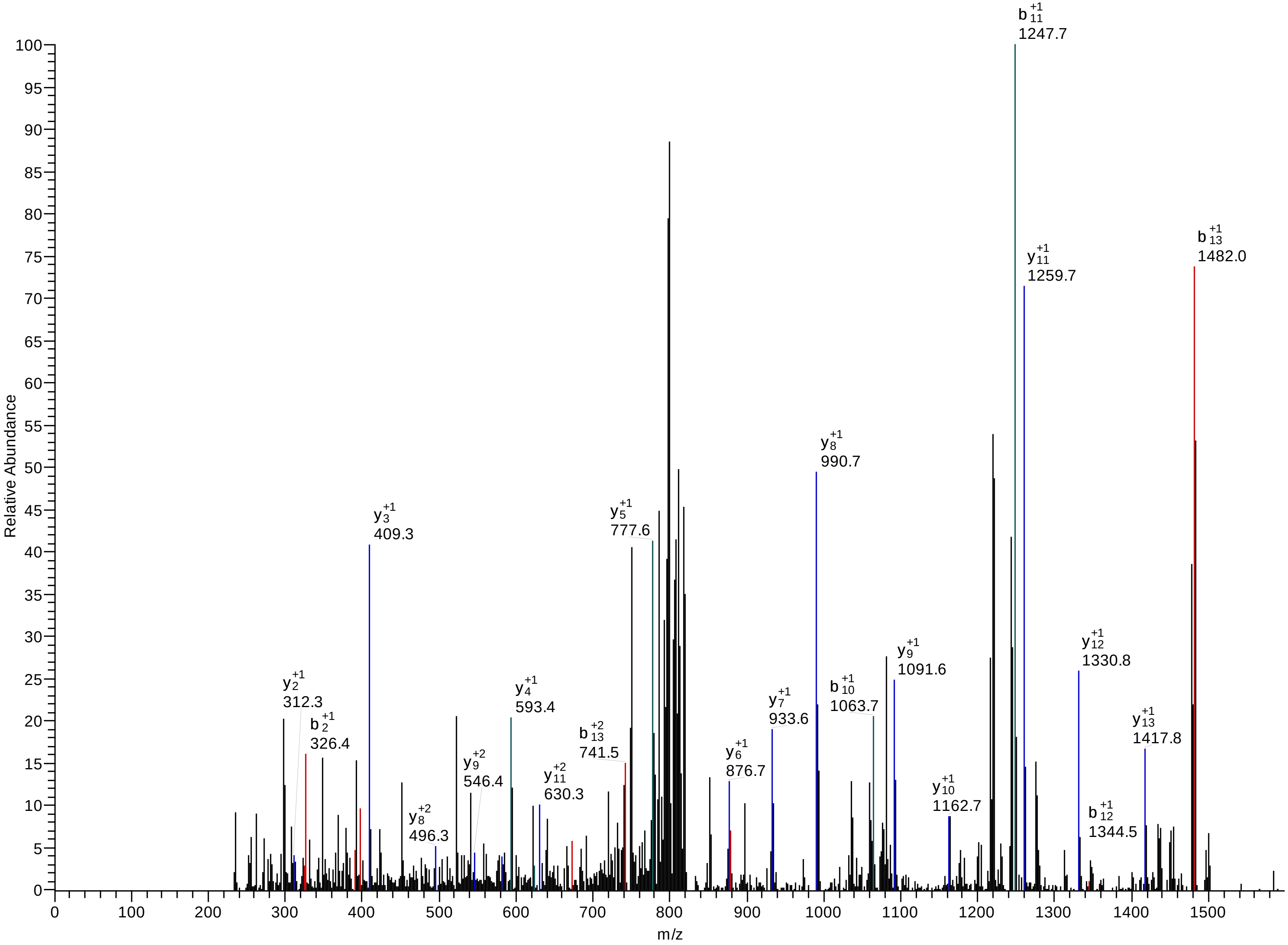

prKme2:2SAPATGGVKpr,me1:1KprPHR H3K27me2:2K36me1:1

#7964-7964 RT:55.09-55.09 NL: 7.39E3

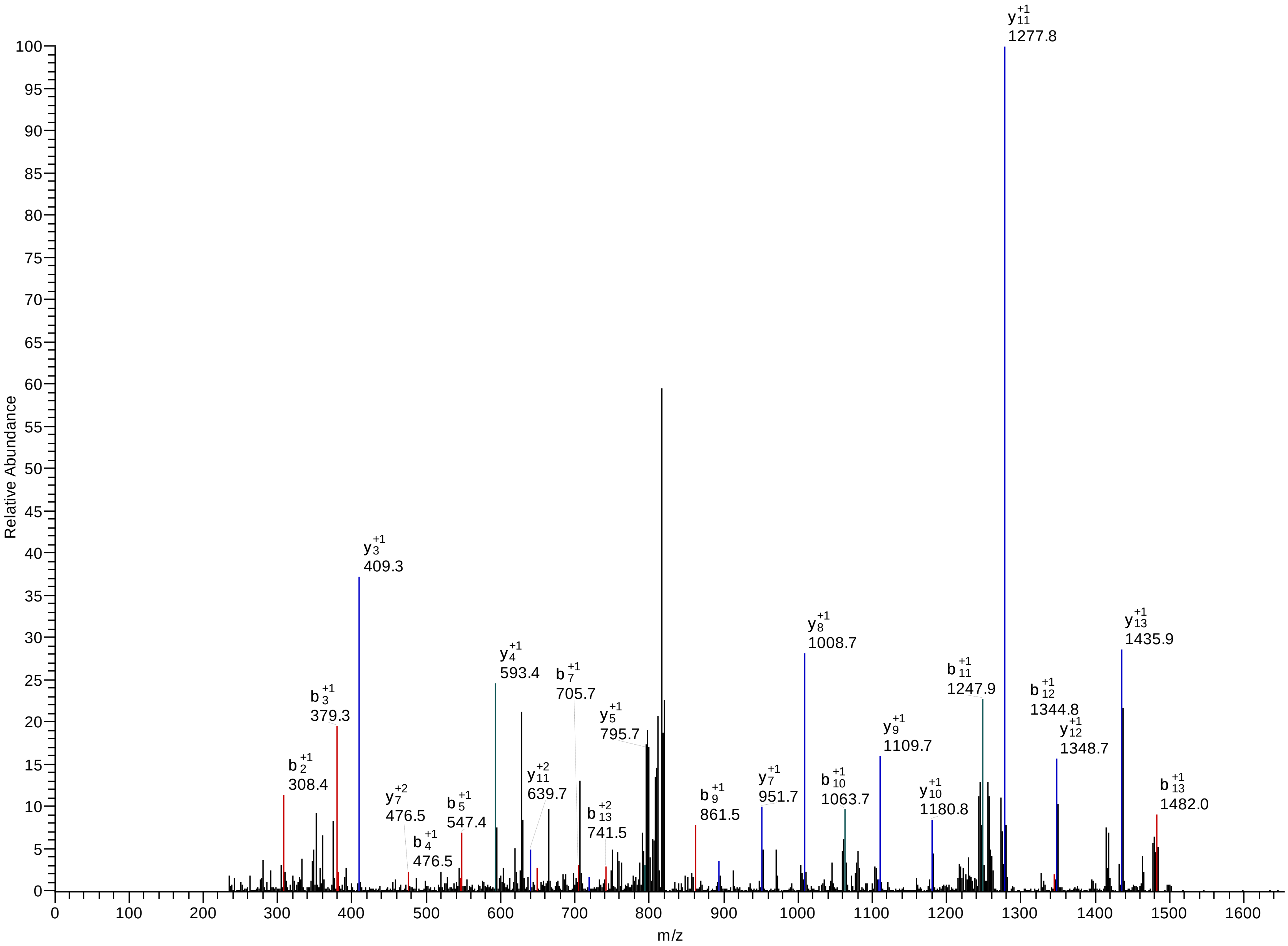

prKpr,me1:1SAPATGGVKme2:2KprPHR H3K27me1:1K36me2:2

#8614-8614 RT:58.94-58.94 NL: 9.96E2

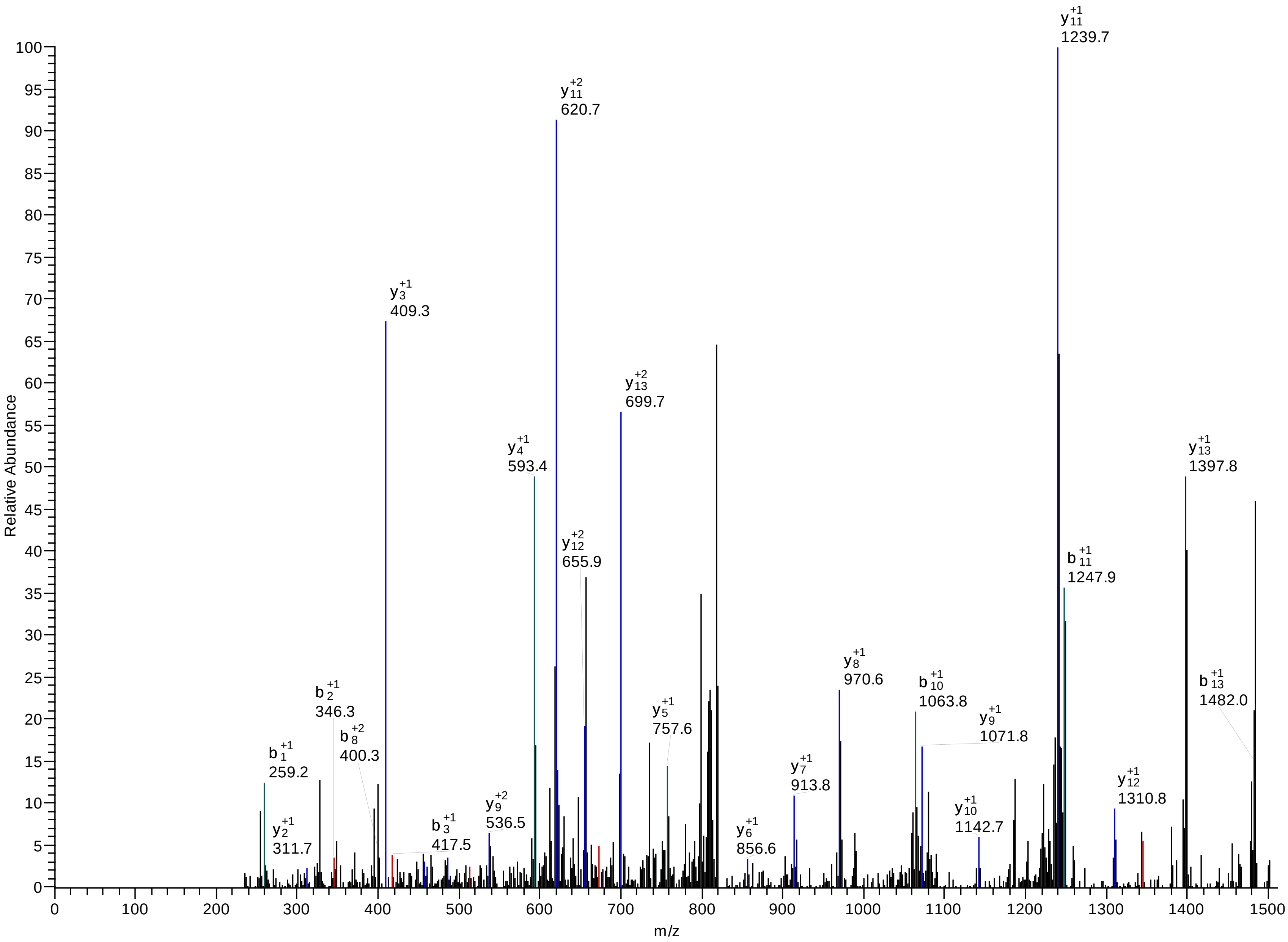

prKme3:0SAPATGGVKpr,me1:1KprPHR

#8140-8140 RT:56.19-56.19 NL: 4.14E5

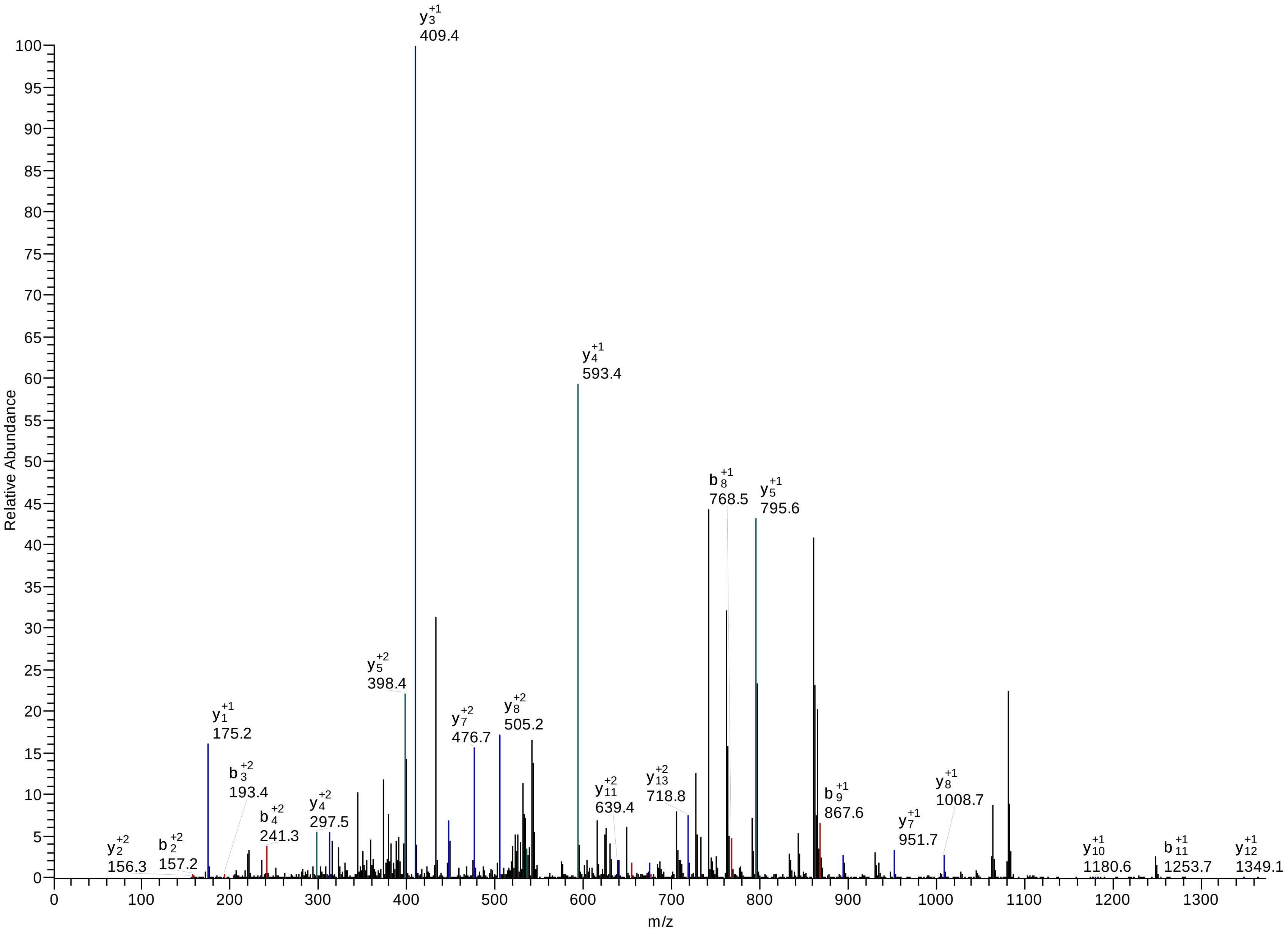

prKpr,me1:0SAPATGGVKme3:1KprPHR H3K27me1:0K36me3:1

#8495-8495 RT:58.12-58.12 NL: 4.54E5

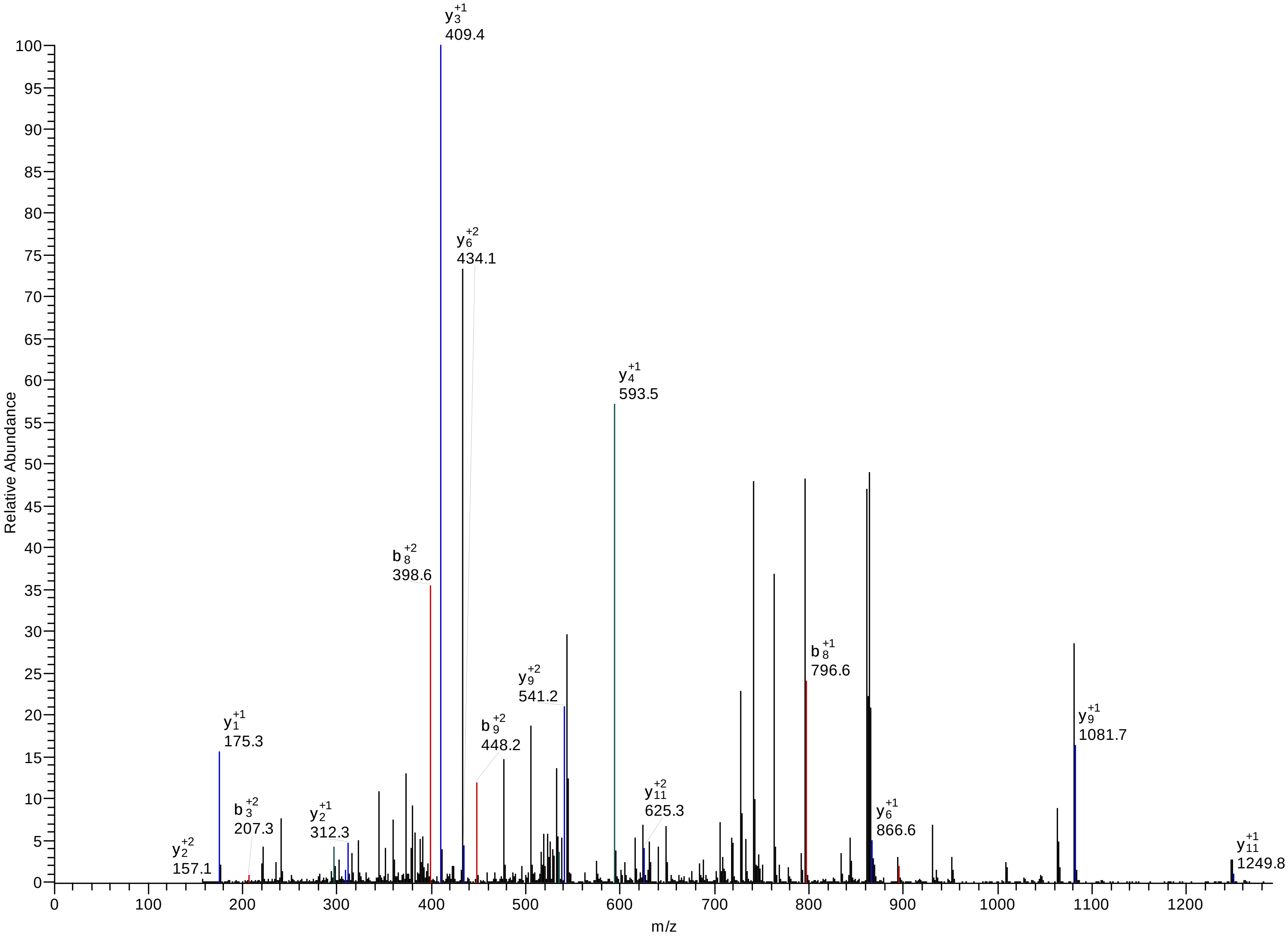

prKme3:1SAPATGGVKpr,me1:1KprPHR H3K27me3:1K36me1:1

#8141-8141 RT:56.20-56.20 NL: 6.42E4

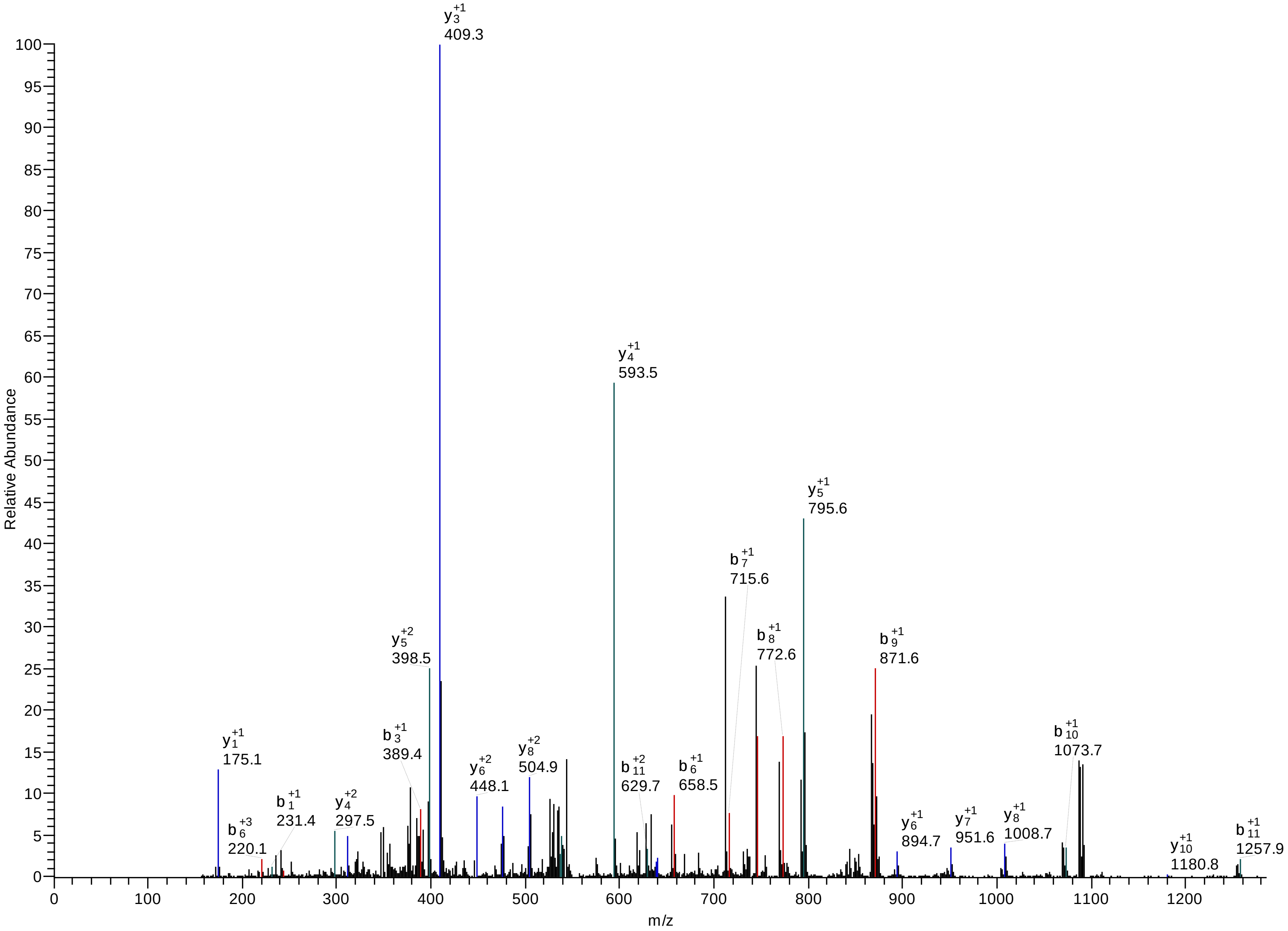

prKpr,me1:1SAPATGGVKme3:1KprPHR H3K27me1:1K36me3:1

#8475-8475 RT:58.01-58.01 NL: 3.70E4

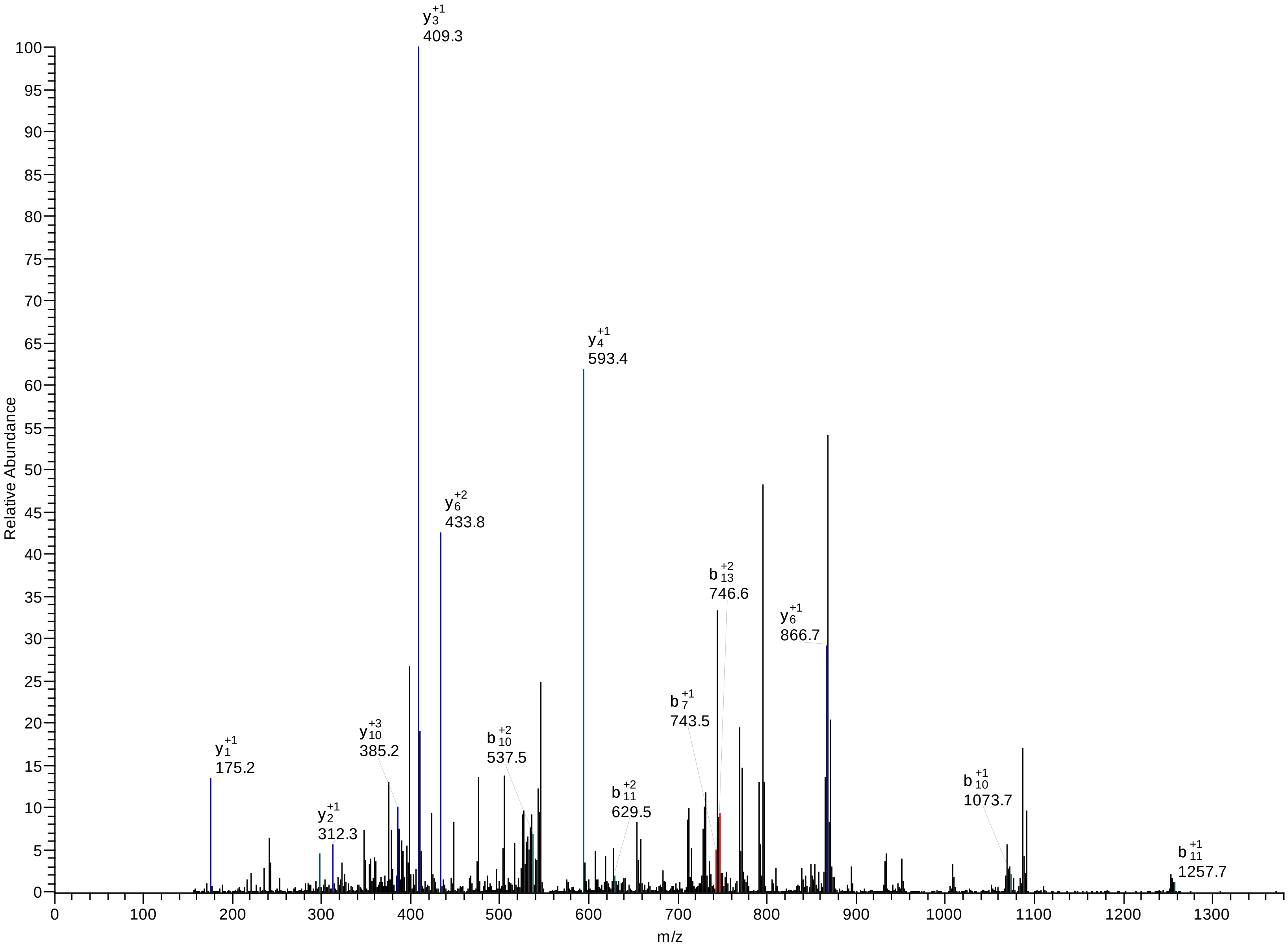

prKme3:3SAPATGGVKpr,me1:0KprPHR H3K27me3:3K36me1:0

#7849-7849 RT:56.01-56.01 NL: 4.26E3

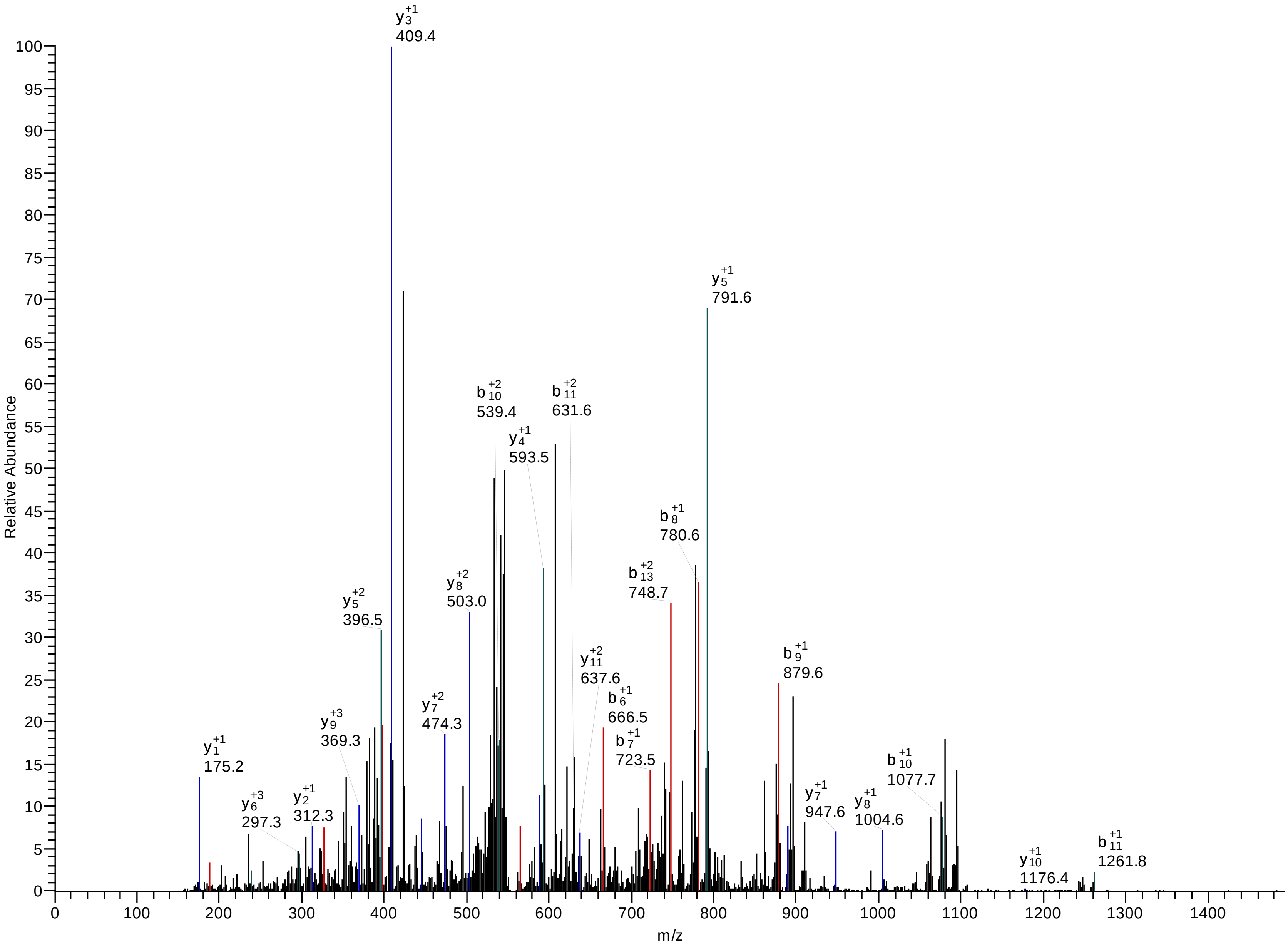

prKpr,me1:0SAPATGGVKme3:3KprPHR H3K27me1:0K36me3:3

#8849-8849 RT:60.34-60.34 NL: 7.26E2

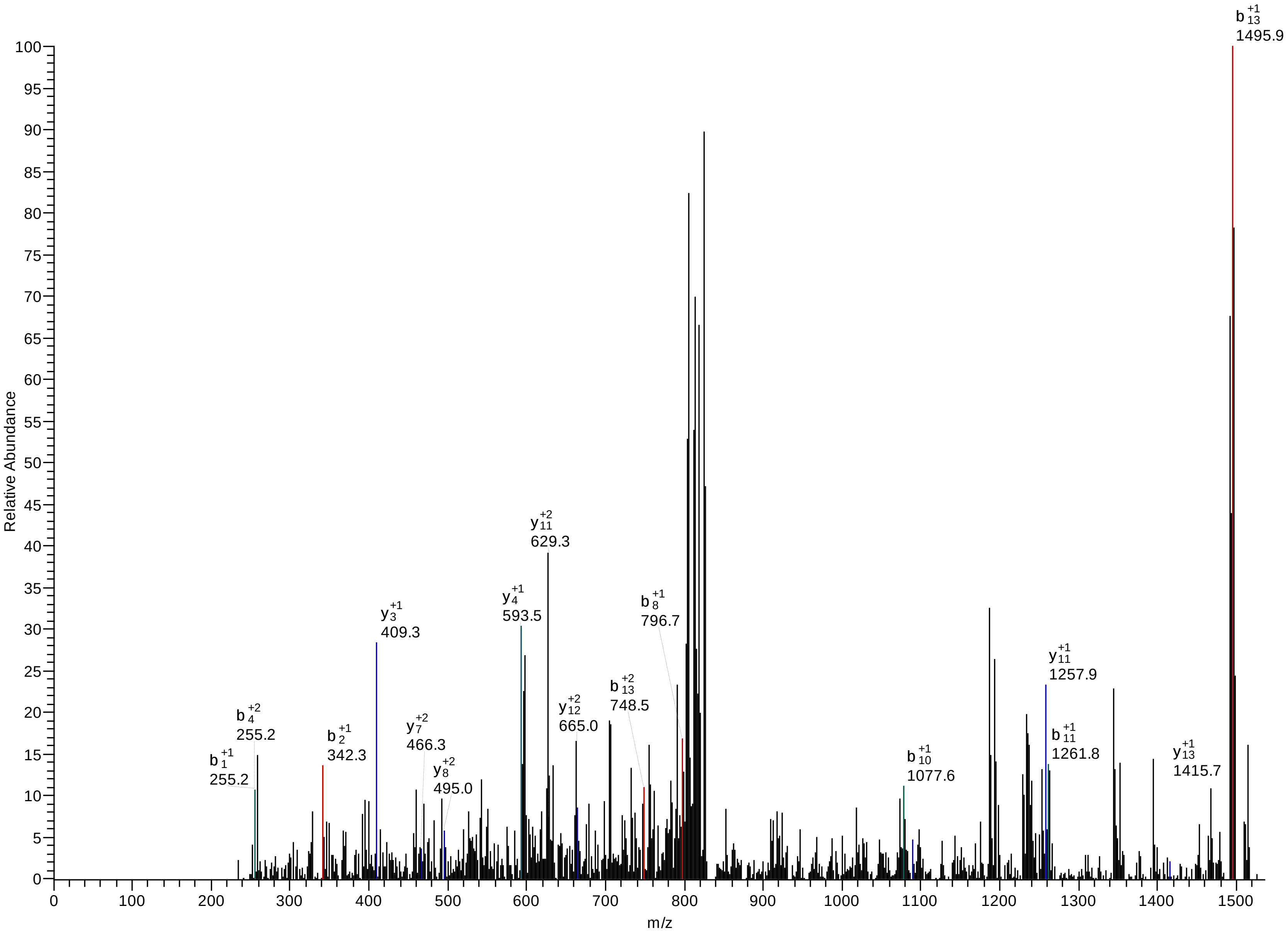

prKme3:3SAPATGGVKpr,me1:1KprPHR H3K27me3:3K36me1:1

#8392-8392 RT:59.27-59.27 NL: 1.93E3

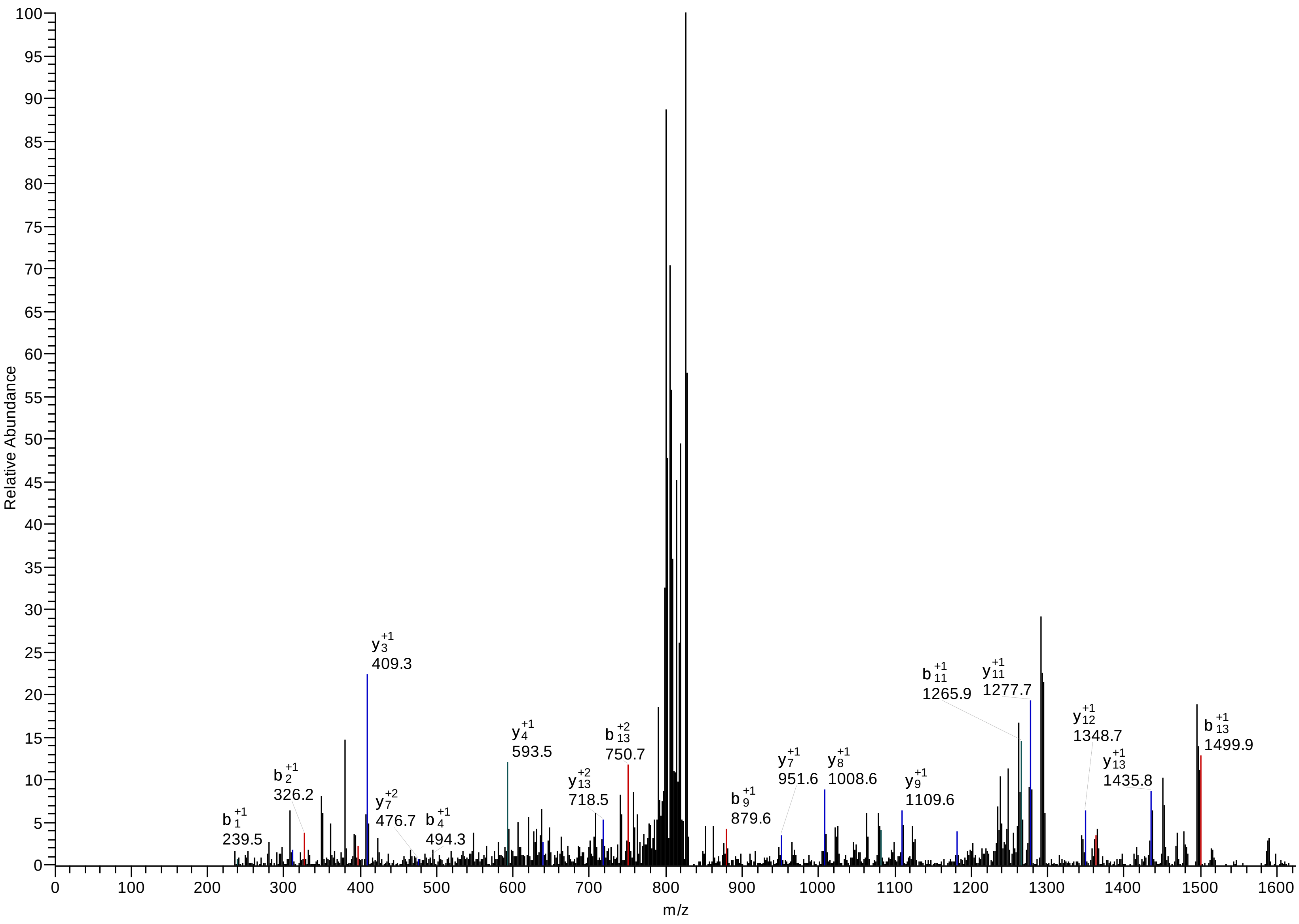

prKpr,me1:1SAPATGGVKme3:3KprPHR H3K27me1:1K36me3:3

#8766-8766 RT:59.85-59.85 NL: 2.84E5

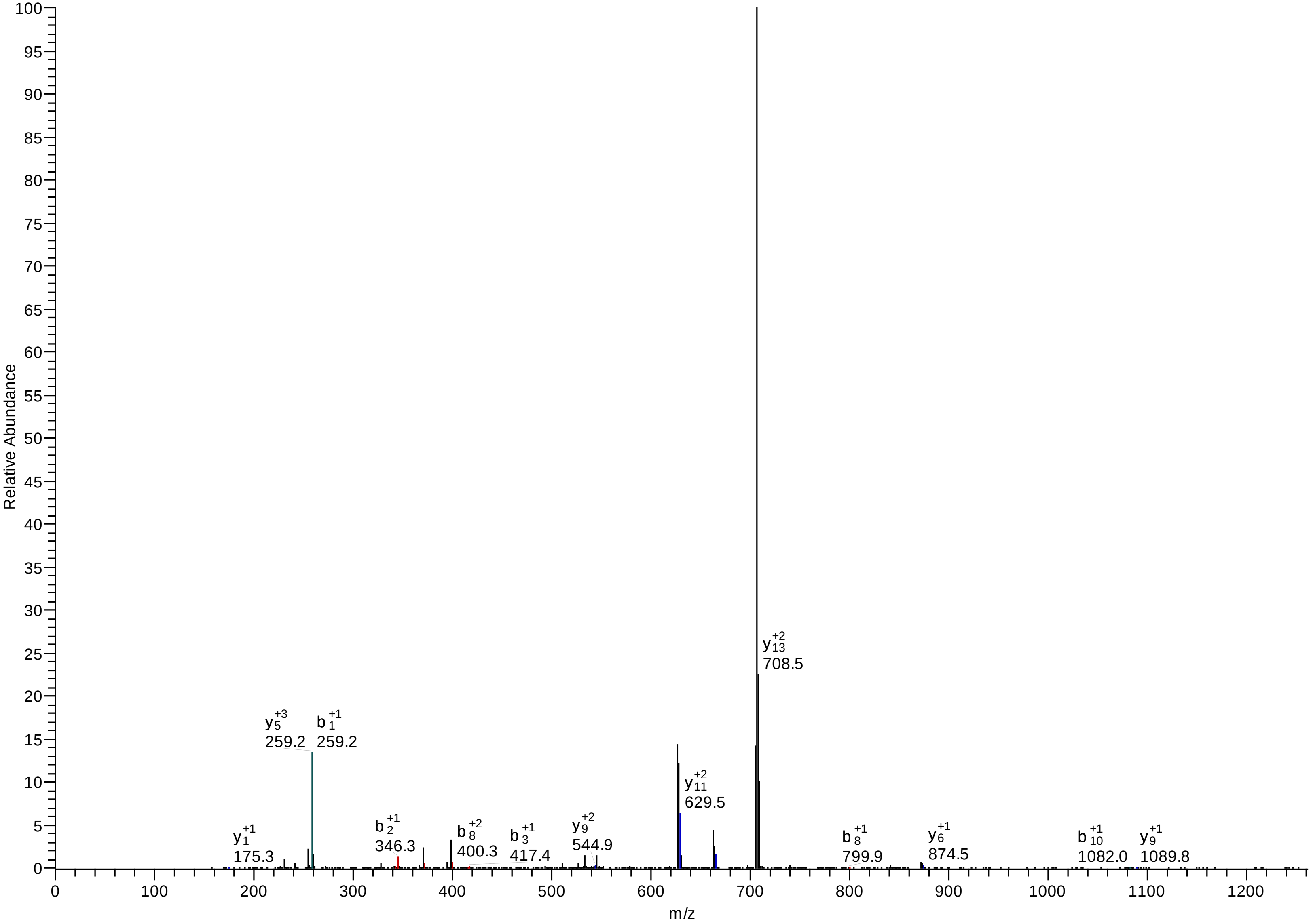

prKme2:0SAPATGGVKme2:1KprPHR H3K27me2:0K36me2:1

#6163-6163 RT:49.33-49.33 NL: 5.18E3

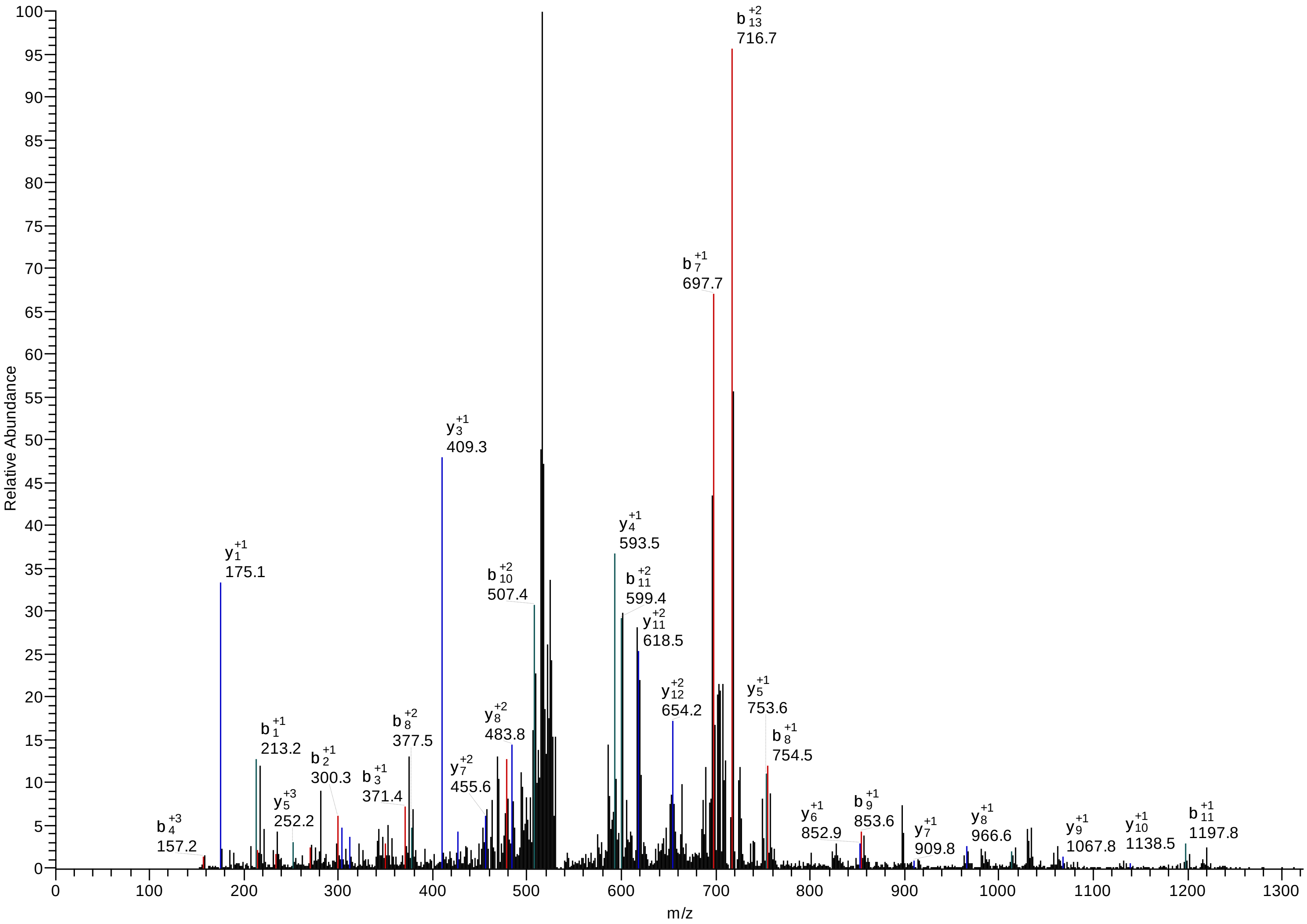

prKpr,me2:1SAPATGGVKpr,me2:1KprPHR   H3K27me2:1K36me2:1

#6696-6696 RT:47.70-47.70 NL: 1.20E4

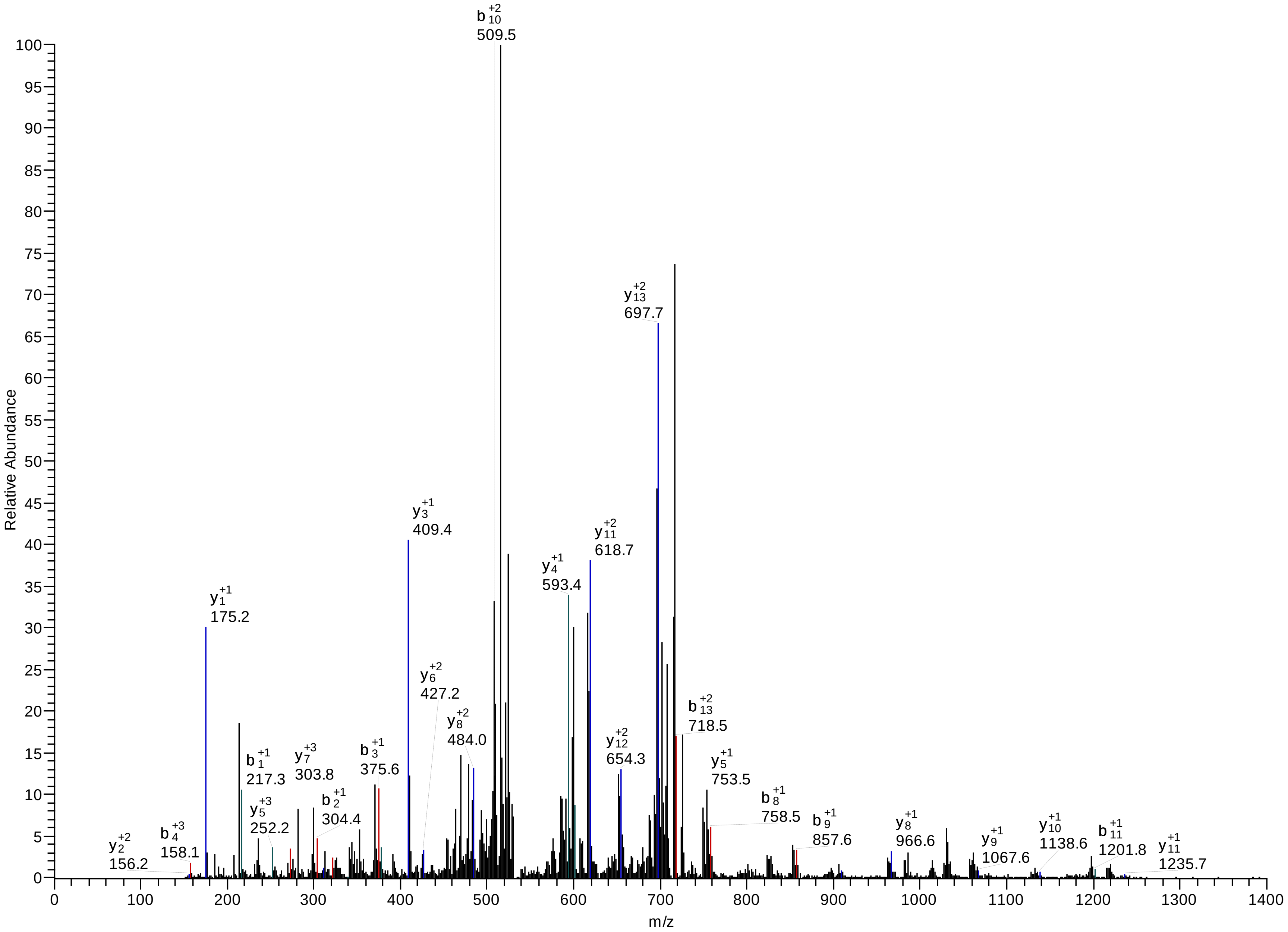

prKpr,me2:1SAPATGGVKpr,me2:2KprPHR   H3K27me2:1K36me2:2

#6700-6700 RT:47.72-47.72 NL: 5.32E3

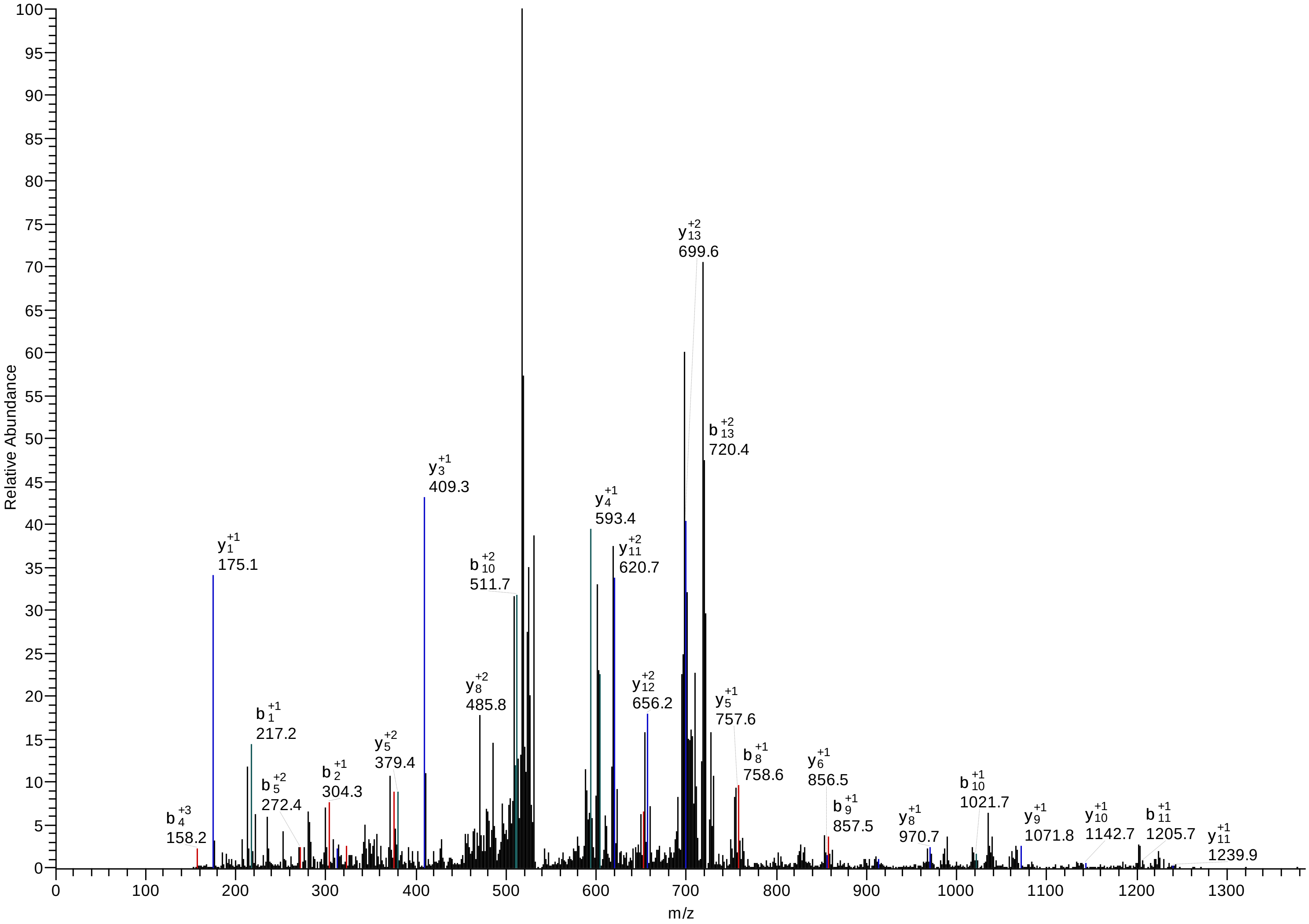

prKpr,me2:2SAPATGGVKpr,me2:2KprPHR   H3K27me2:2K36me2:2

#7226-7226 RT:50.74-50.74 NL: 1.36E3

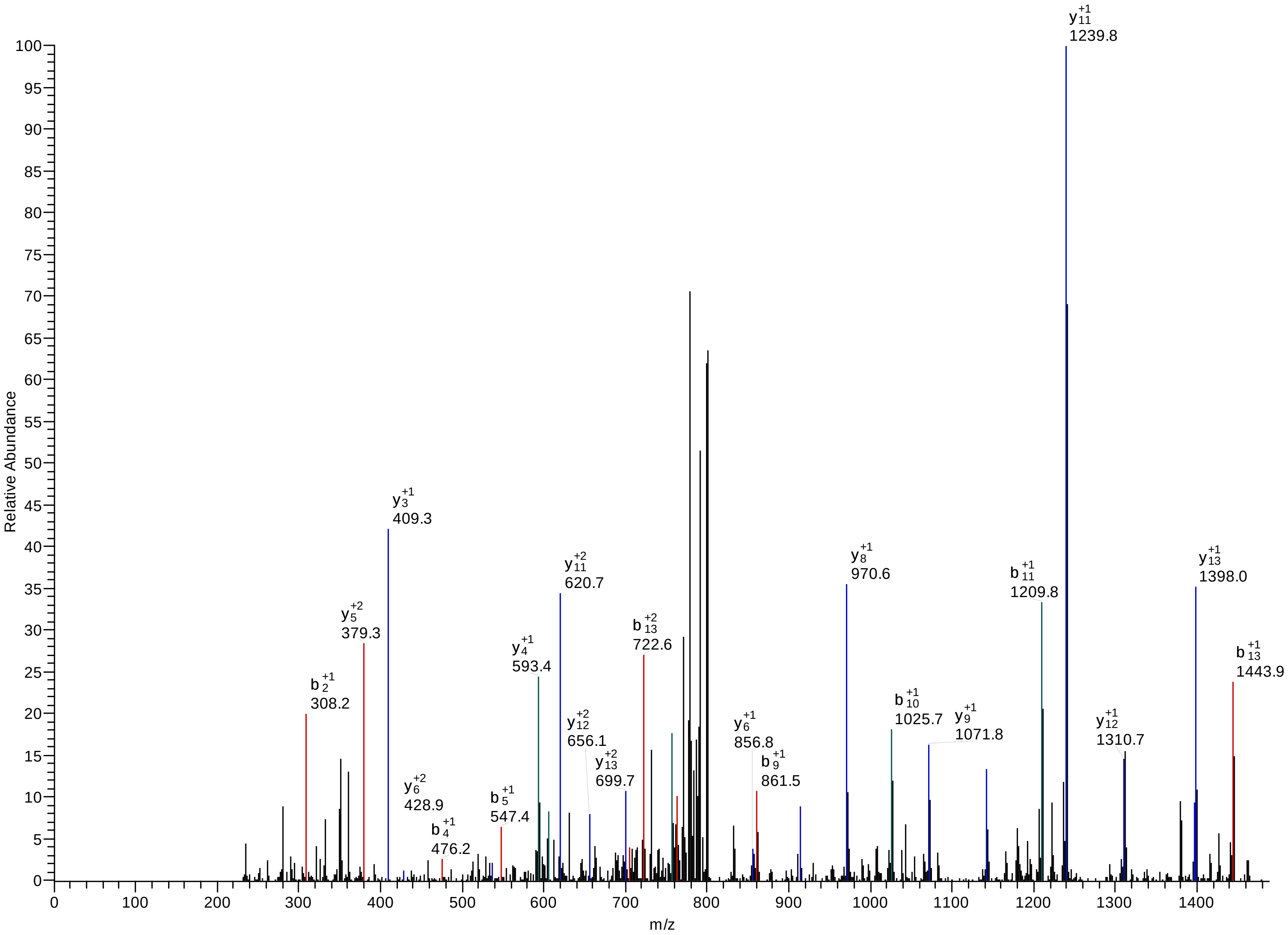

prKme3:0SAPATGGVKme2:1KprPHR H3K27me3:0K36me2:1

#5992-5992 RT:55.40-55.40 NL: 9.66E3

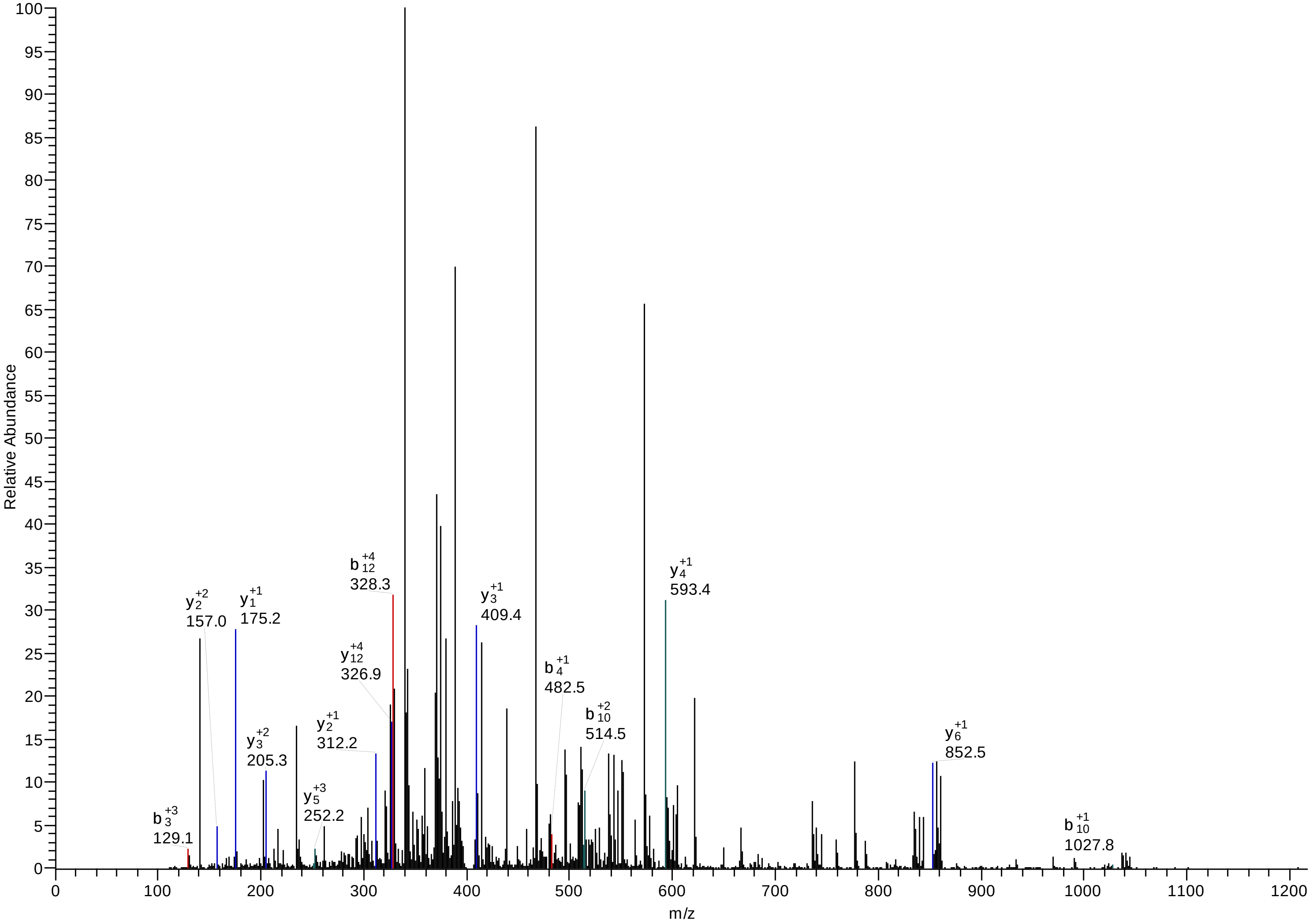

prKme3:0SAPATGGVKme2:2KprPHR H3K27me3:0K36me2:2

#6486-6486 RT:48.29-48.29 NL: 1.82E5

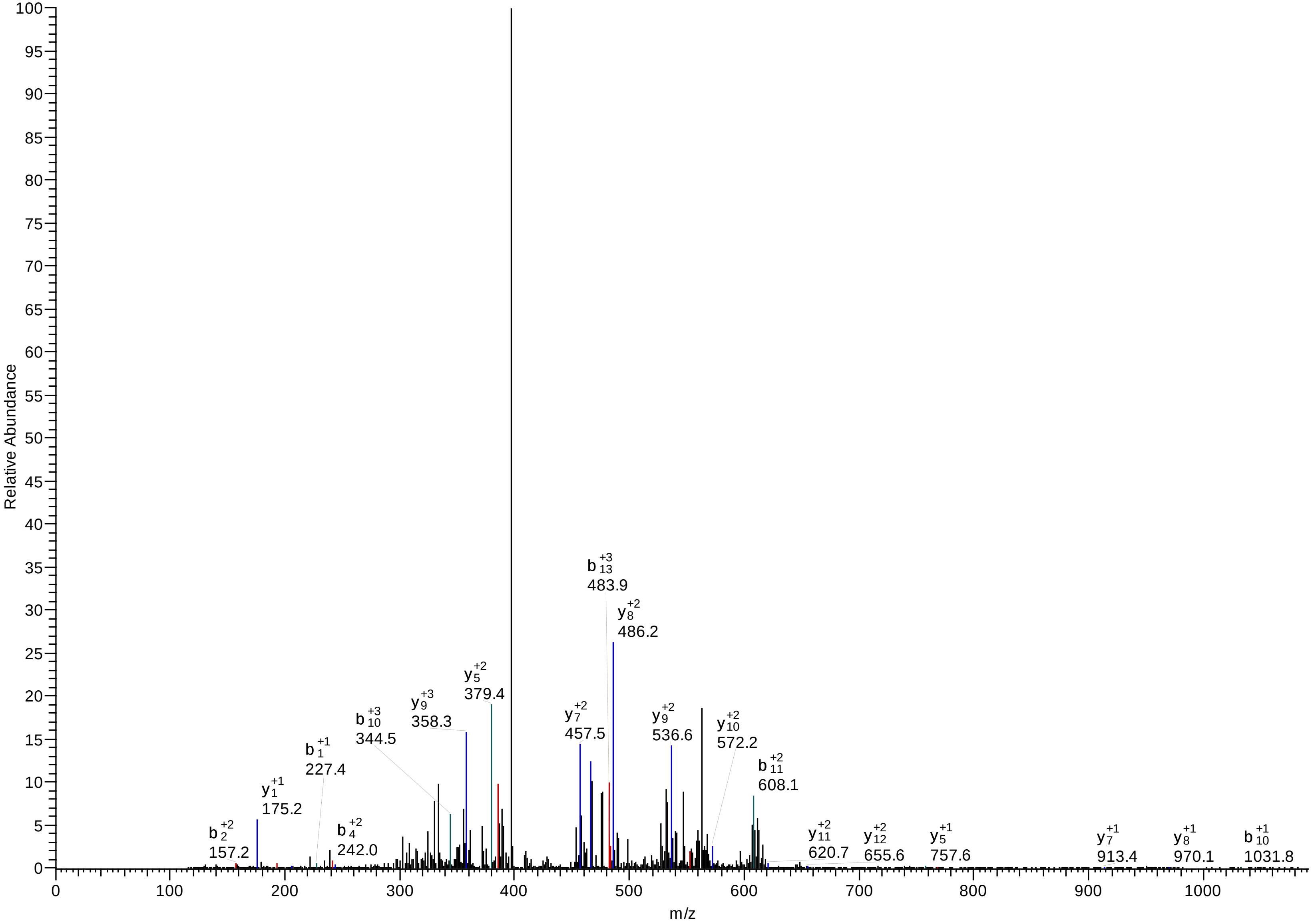

prKme3:1SAPATGGVKme2:2KprPHR H3K27me3:1K36me2:2

#6496-6496 RT:48.35-48.35 NL: 4.18E4

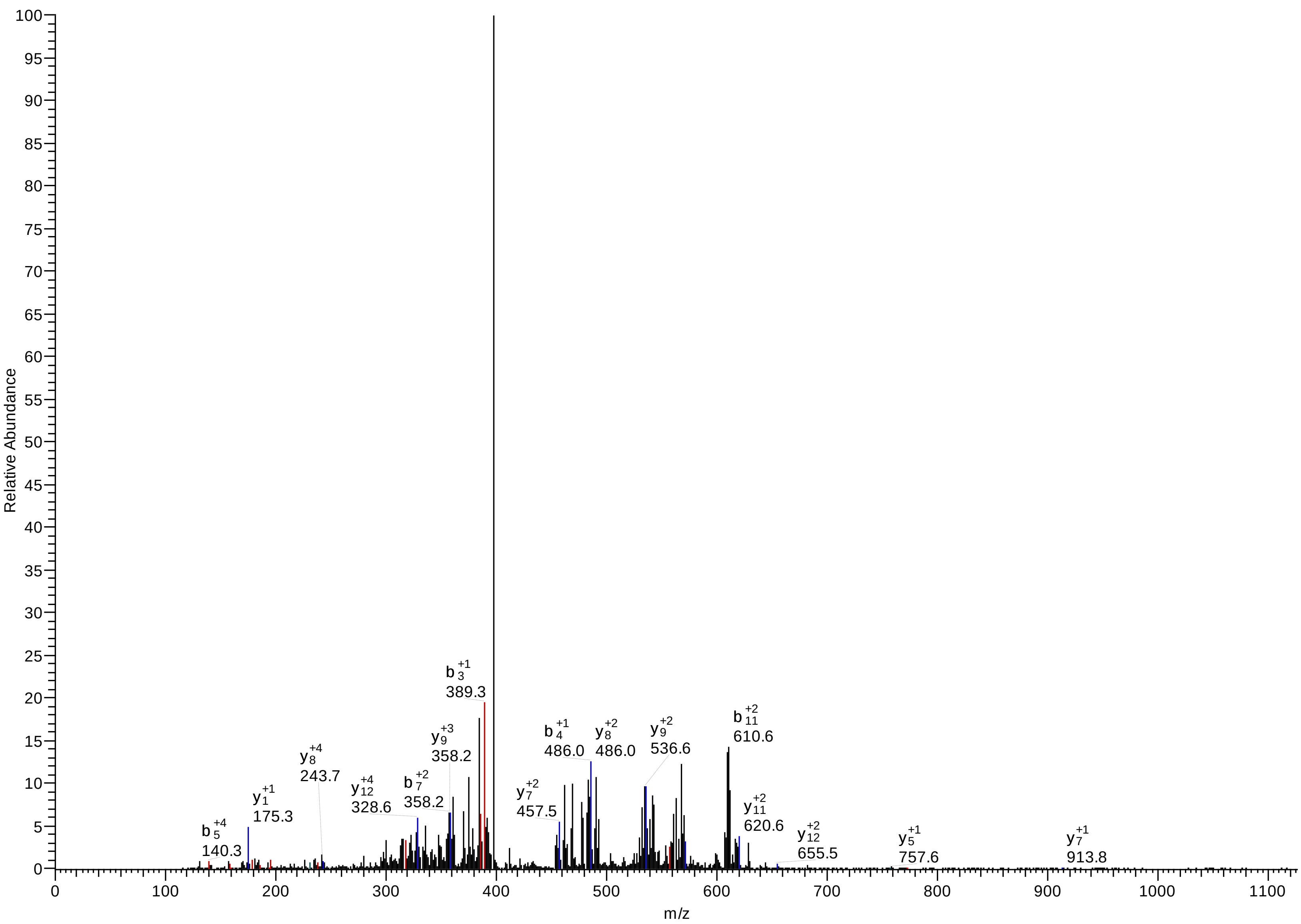

prKme3:2SAPATGGVKme2:2KprPHR H3K27me3:2K36me2:2

#6904-6904 RT:49.05-49.05 NL: 4.54E3

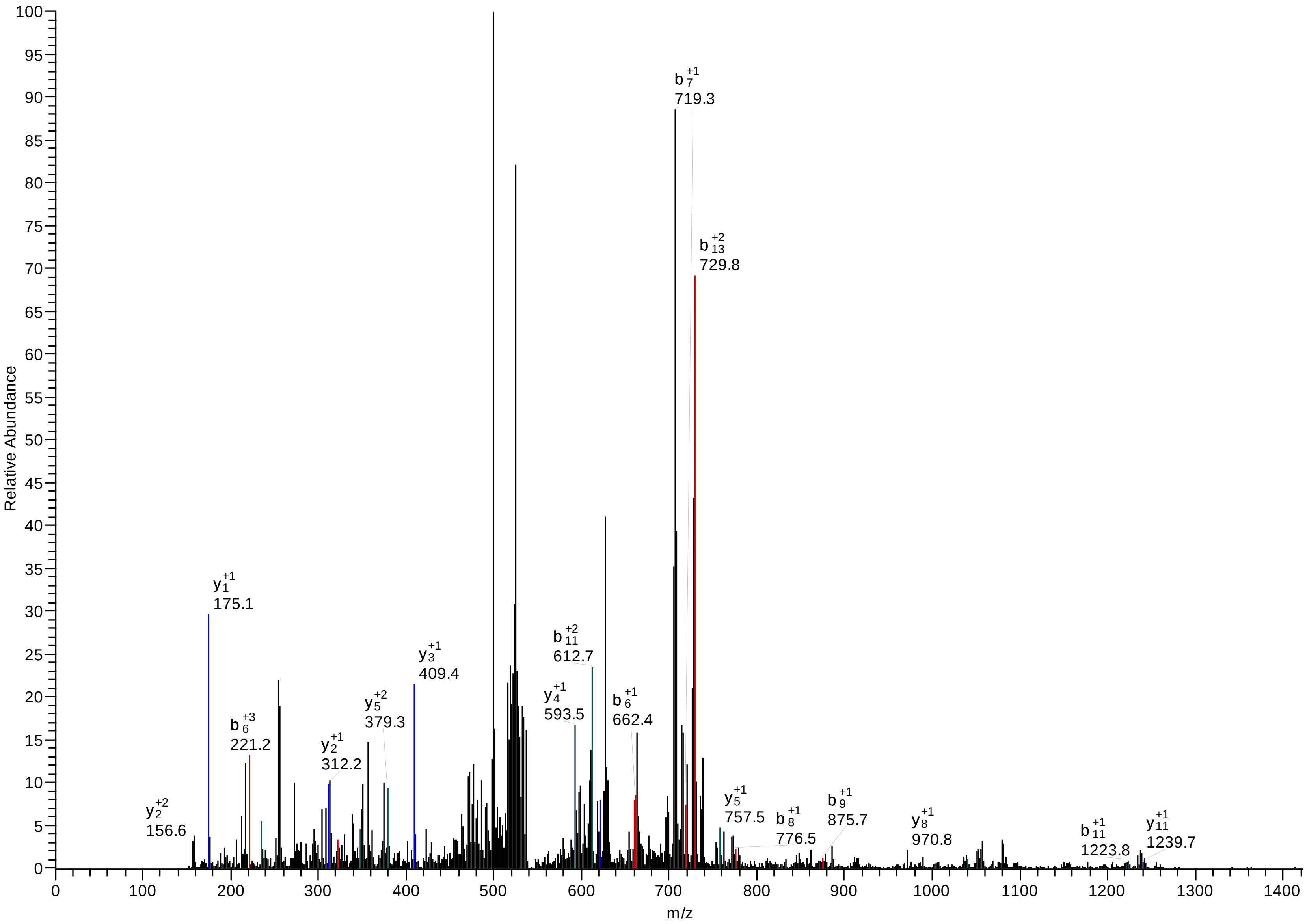

prKme3:3SAPATGGVKme2:2KprPHR H3K27me3:3K36me2:2

#6923-6923 RT:49.16-49.16 NL: 5.59E4

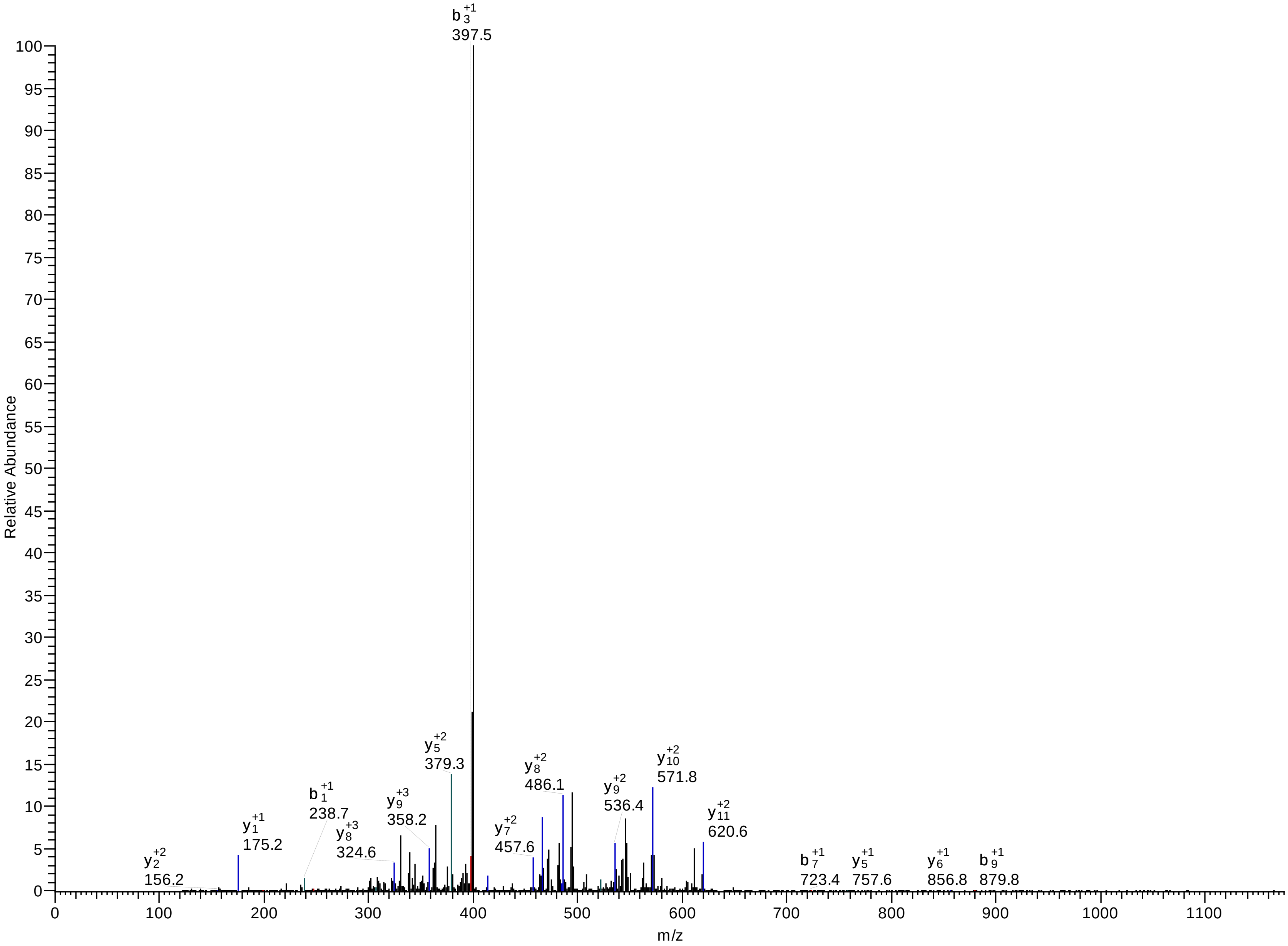

H3 73-83

prEIAQDFKpr,me1:1TDLR H3K79me1:1

#13451-13451 RT:99.78-99.78 NL: 6.41E3

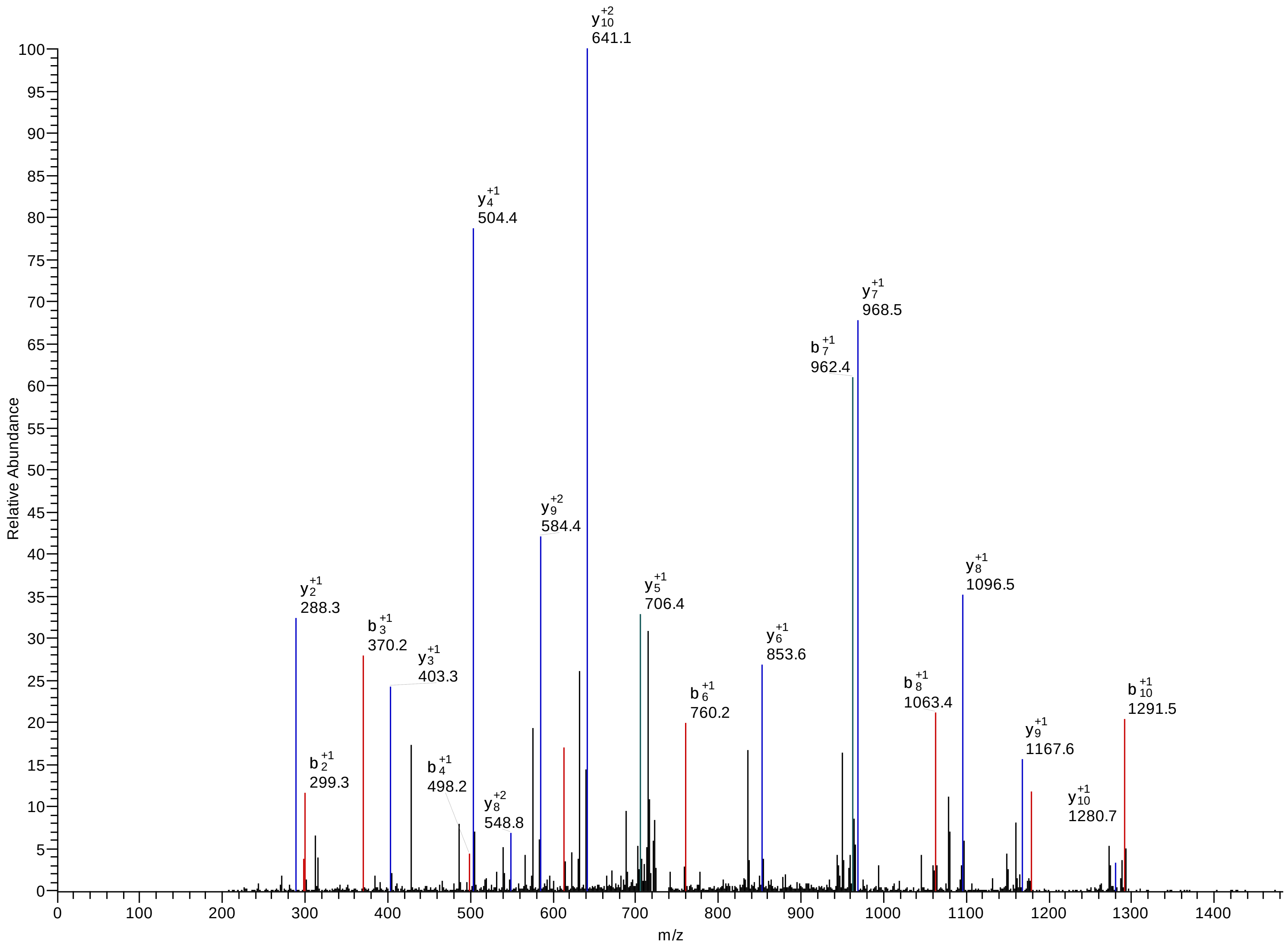

prEIAQDFKme2:1TDLR H3K79me2:1

#15179-15179 RT:98.21-98.21 NL: 5.65E4

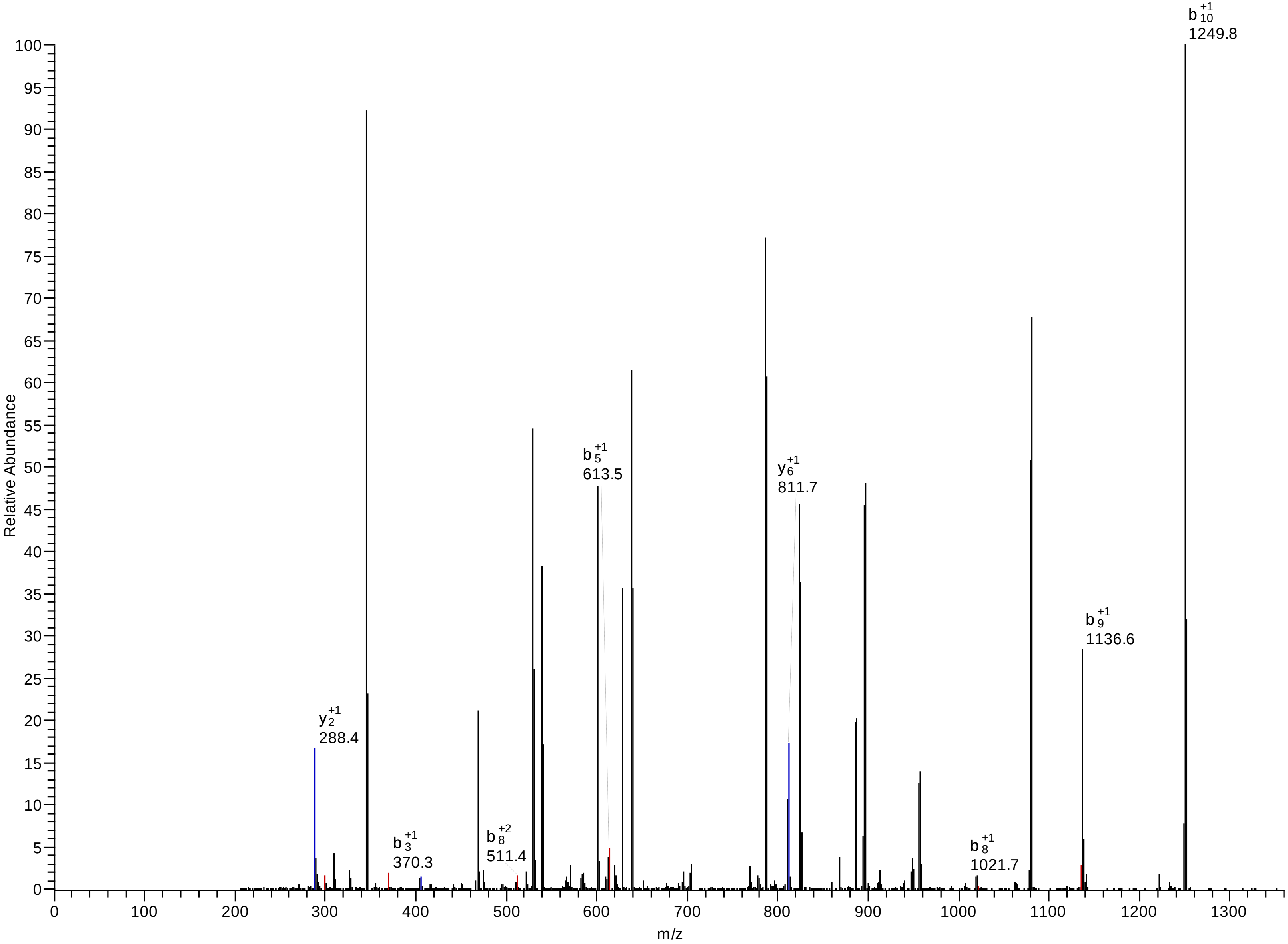

prEIAQDFKme2:2TDLR H3K79me2:2

#10569-10569 RT:82.10-82.10 NL: 5.28E4

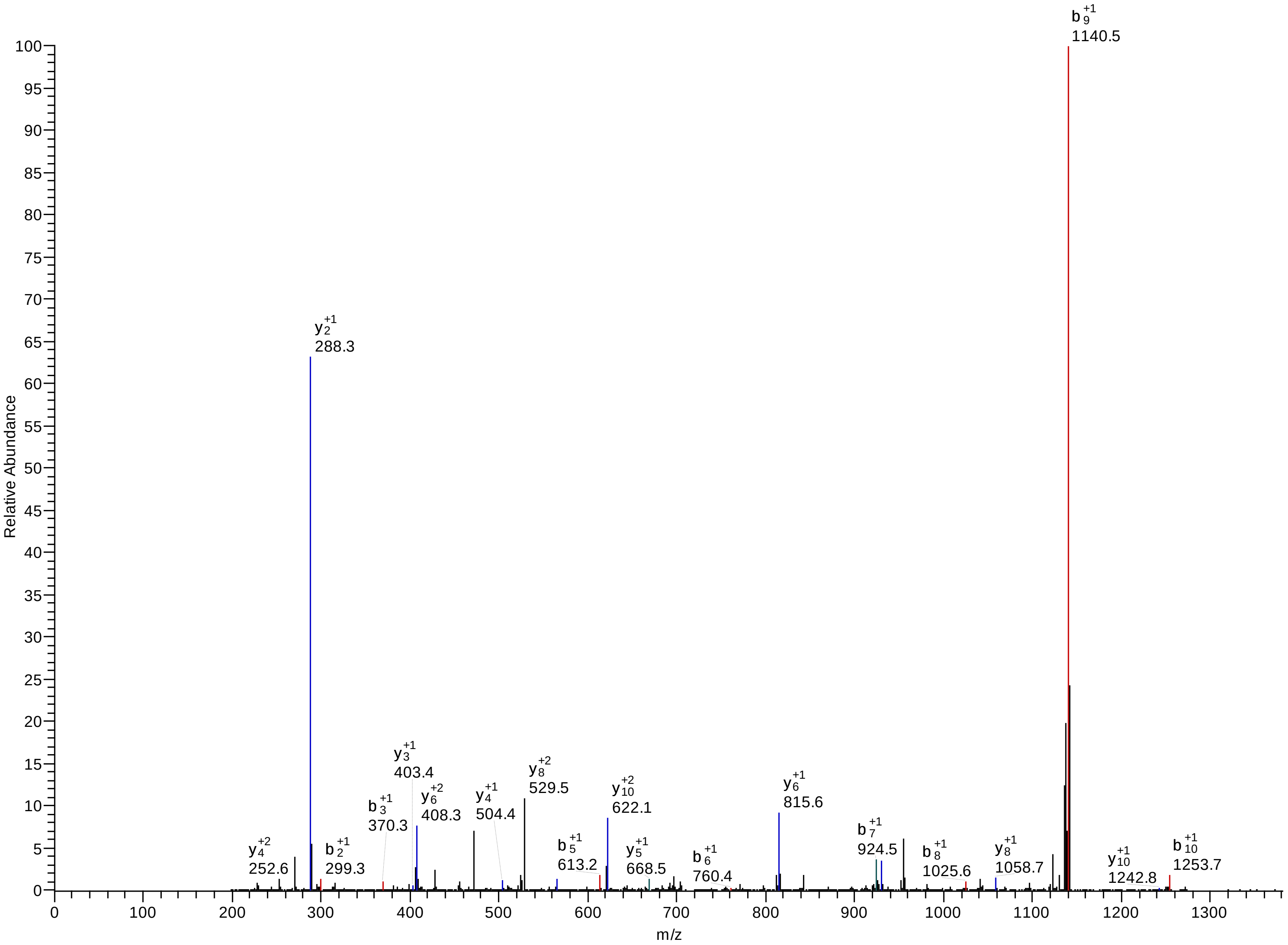

H4 1 -17

acSGRme1:1GKprGGKprGLGKprGGAKprR H4R3me1:1

#8181-8181 RT:67.92-67.92 NL: 1.09E3

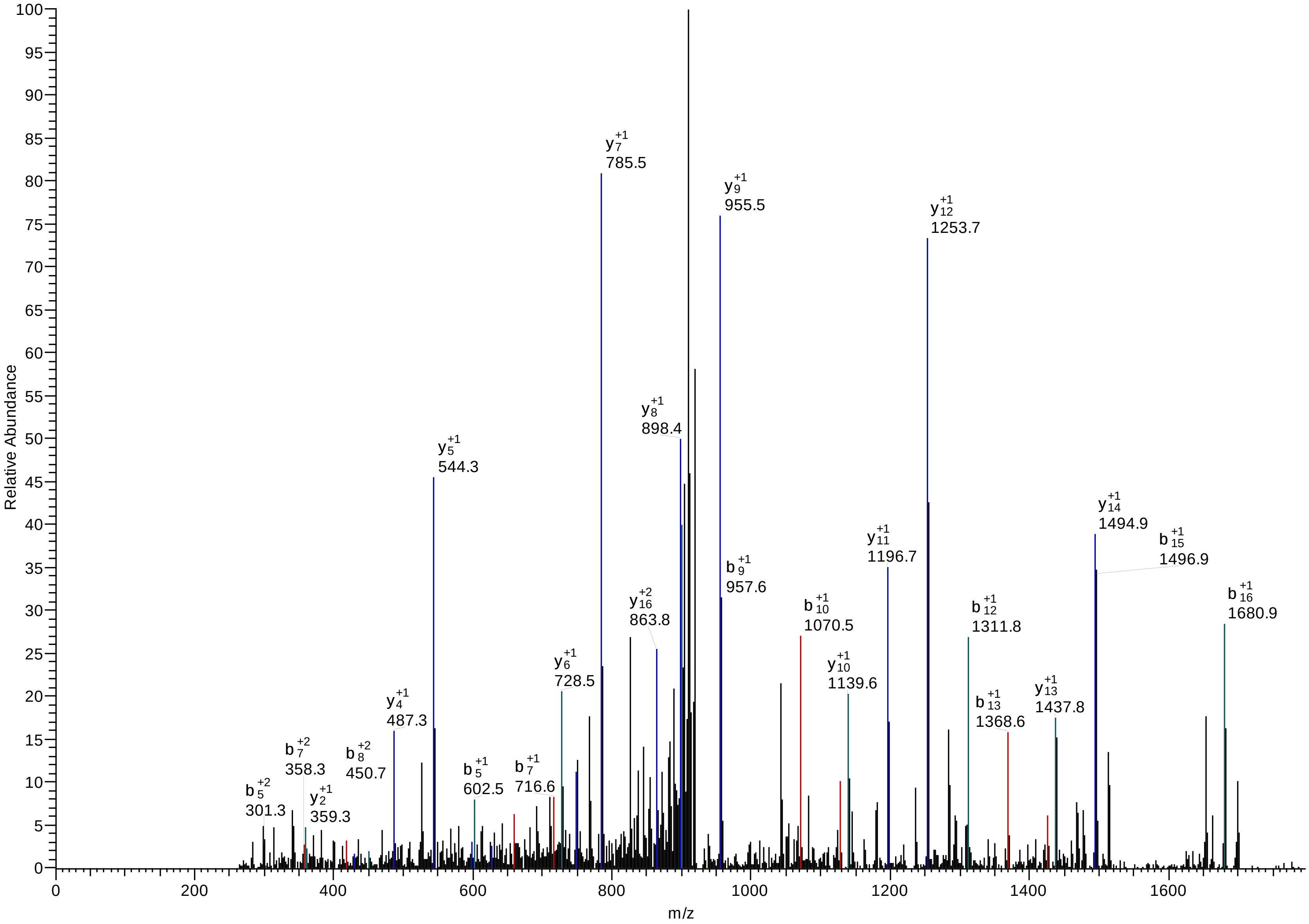

H4 20-23

prKpr,me1:1VLR H4K20me1:1

Me_kinetics_T1_030809_090309012523 #9953 RT: 66.88 AV: 1 NL: 2.39E5
F: ITMS + c NSI d Full ms2 323.23

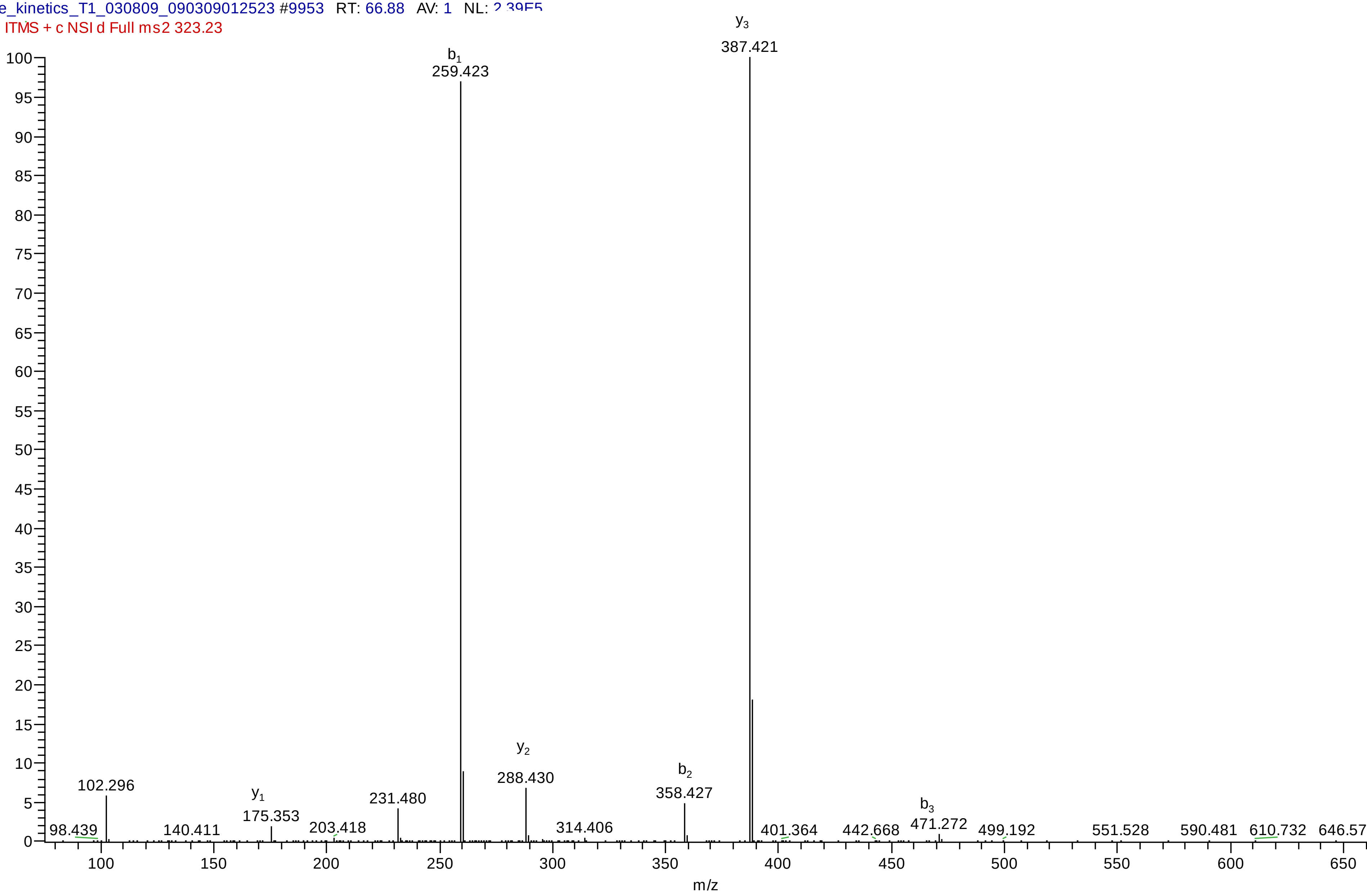

prKme2:1VLR H4K20me2:1

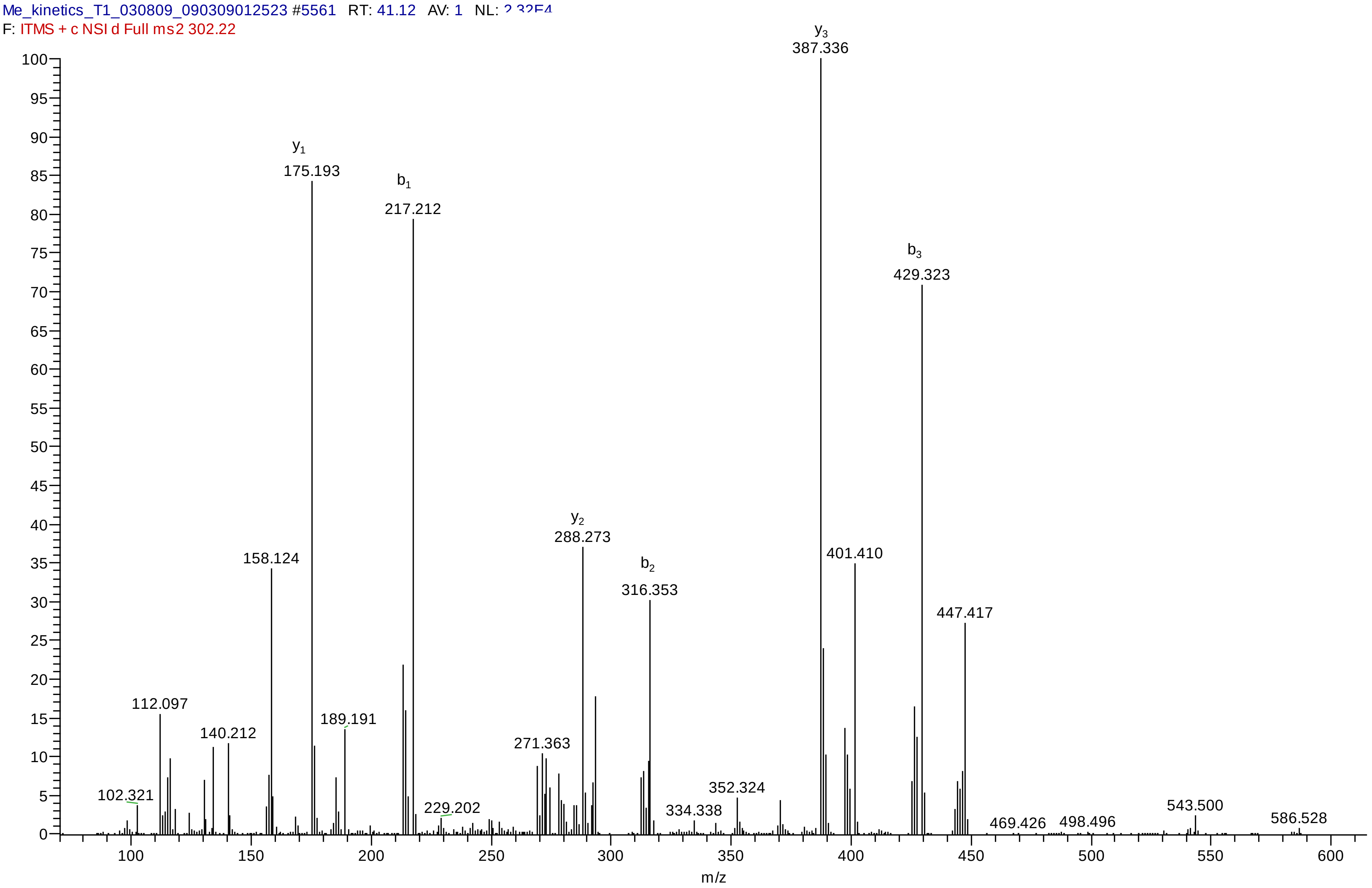

prKme2:2VLR H4K20me2:2

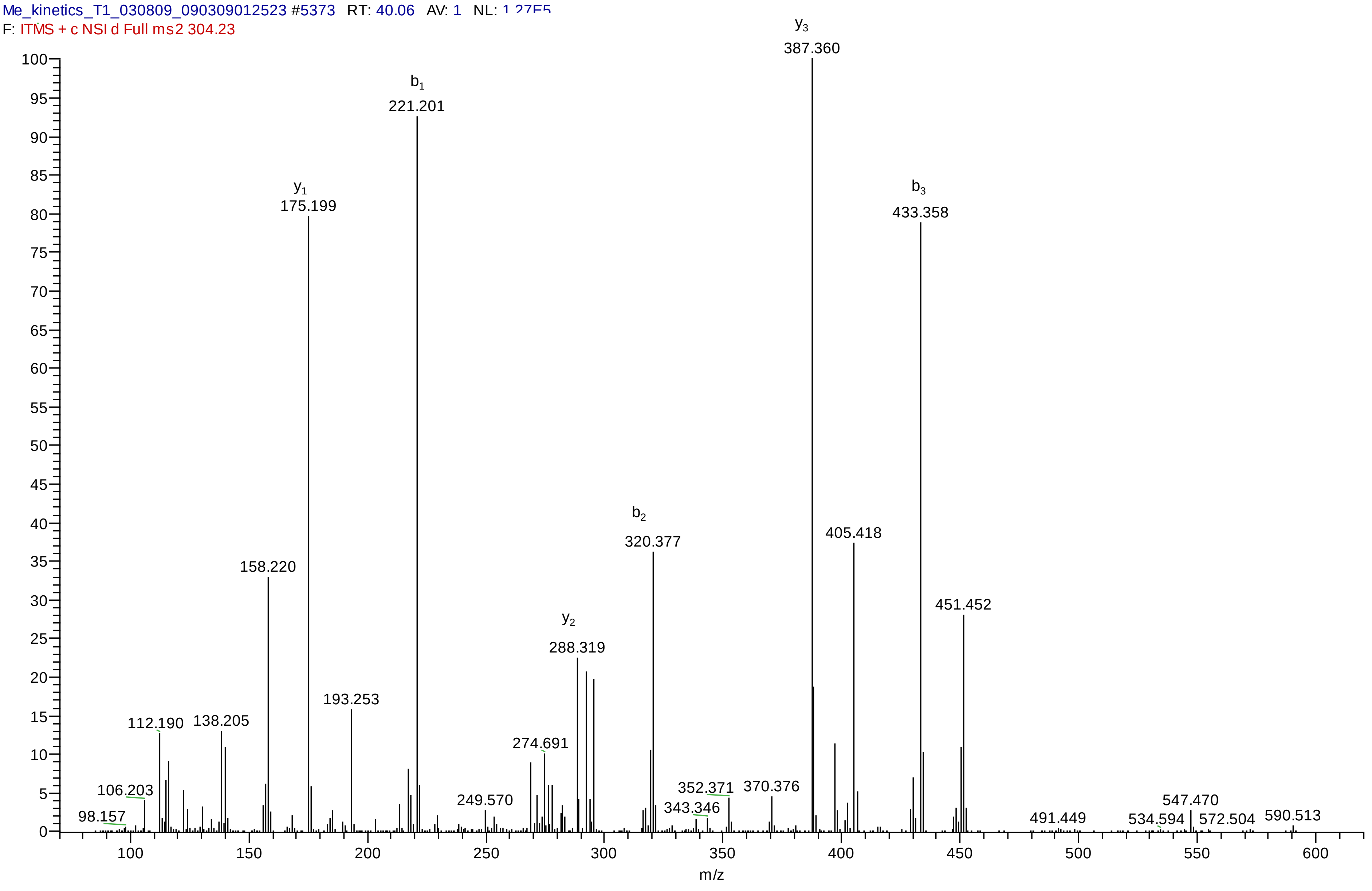

prKme3:1VLR H4K20me3:1

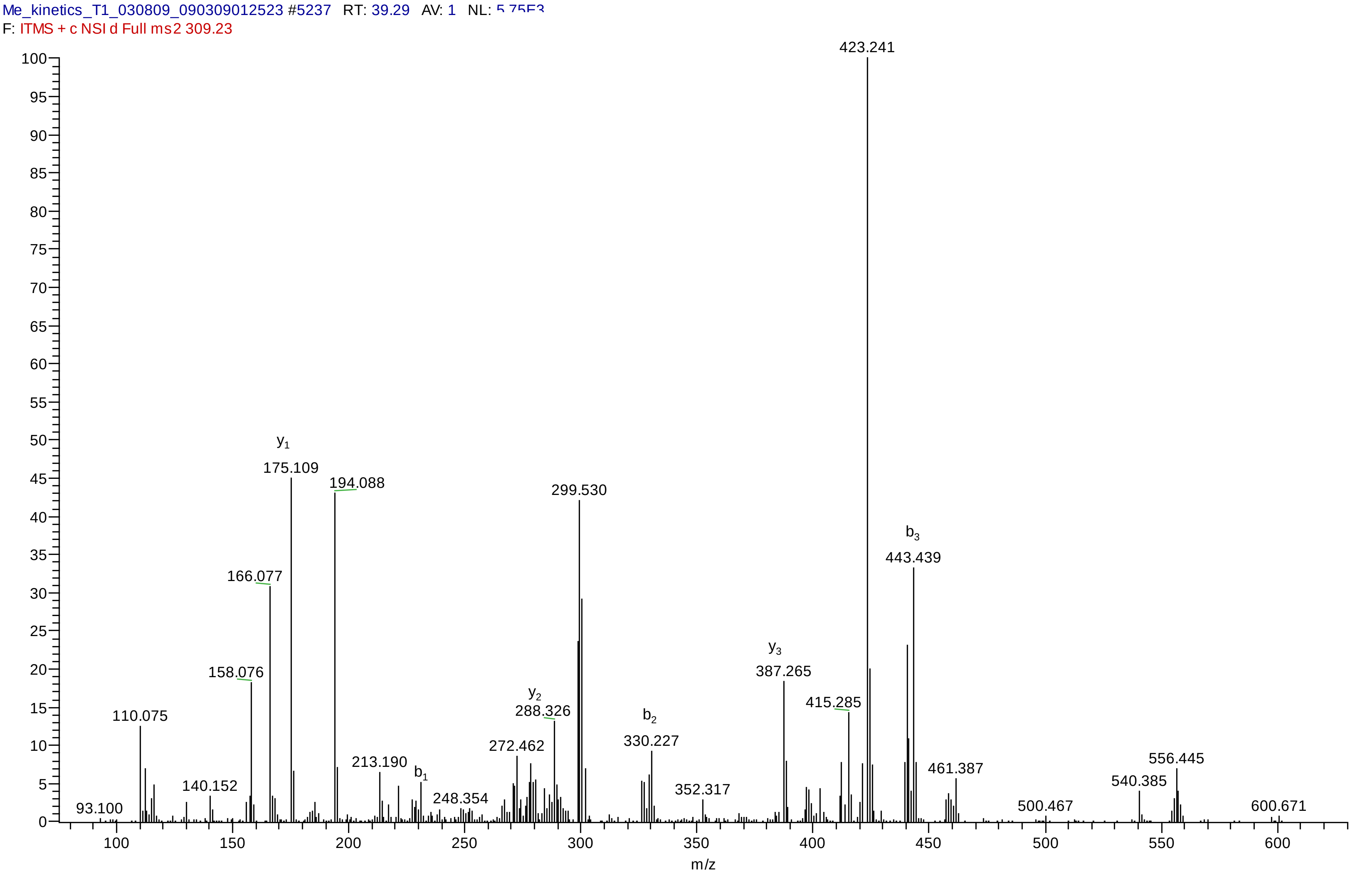

prKme3:2VLR H4K20me3:2

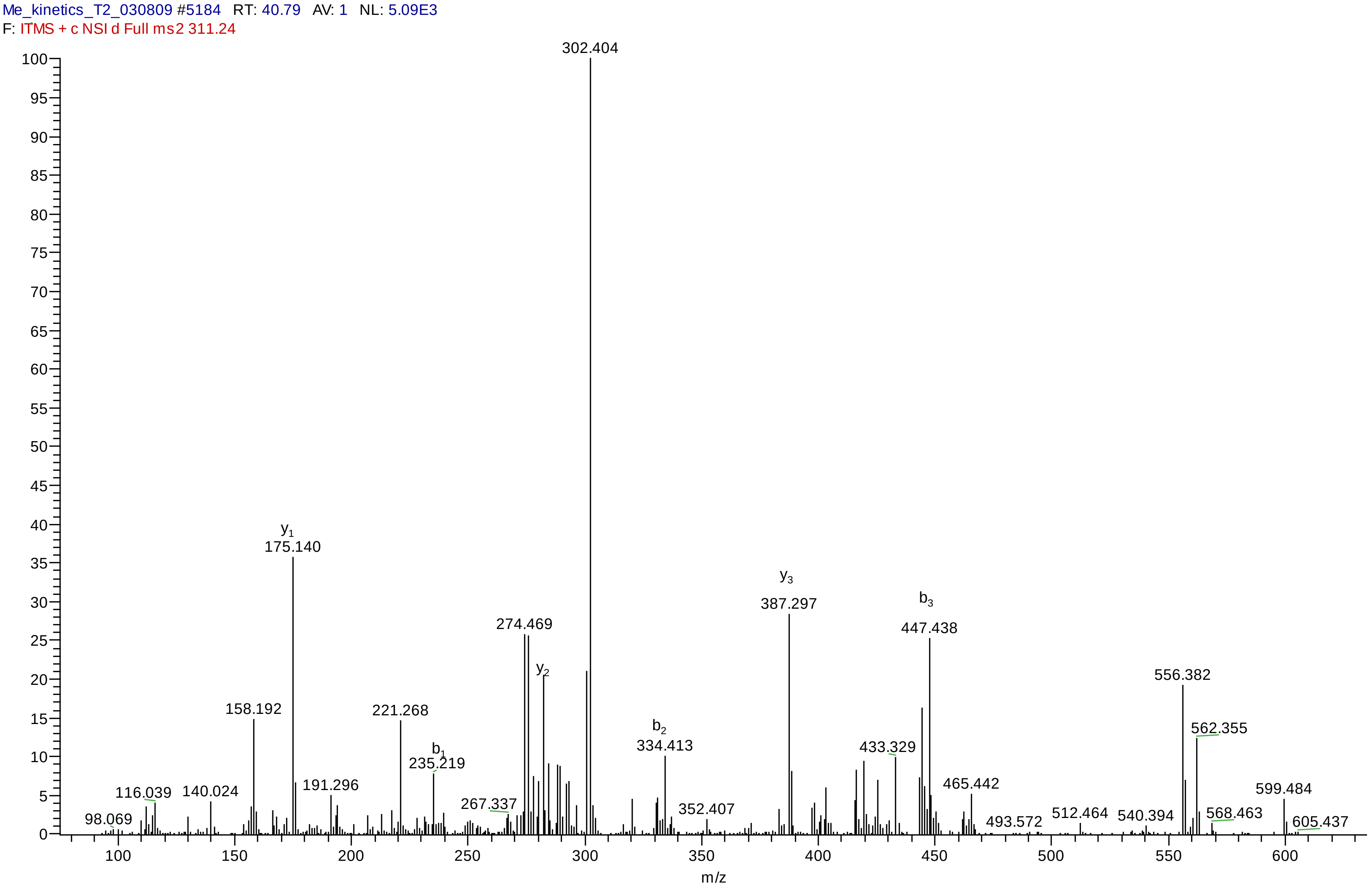

prKme3:3VLR H4K20me3:3

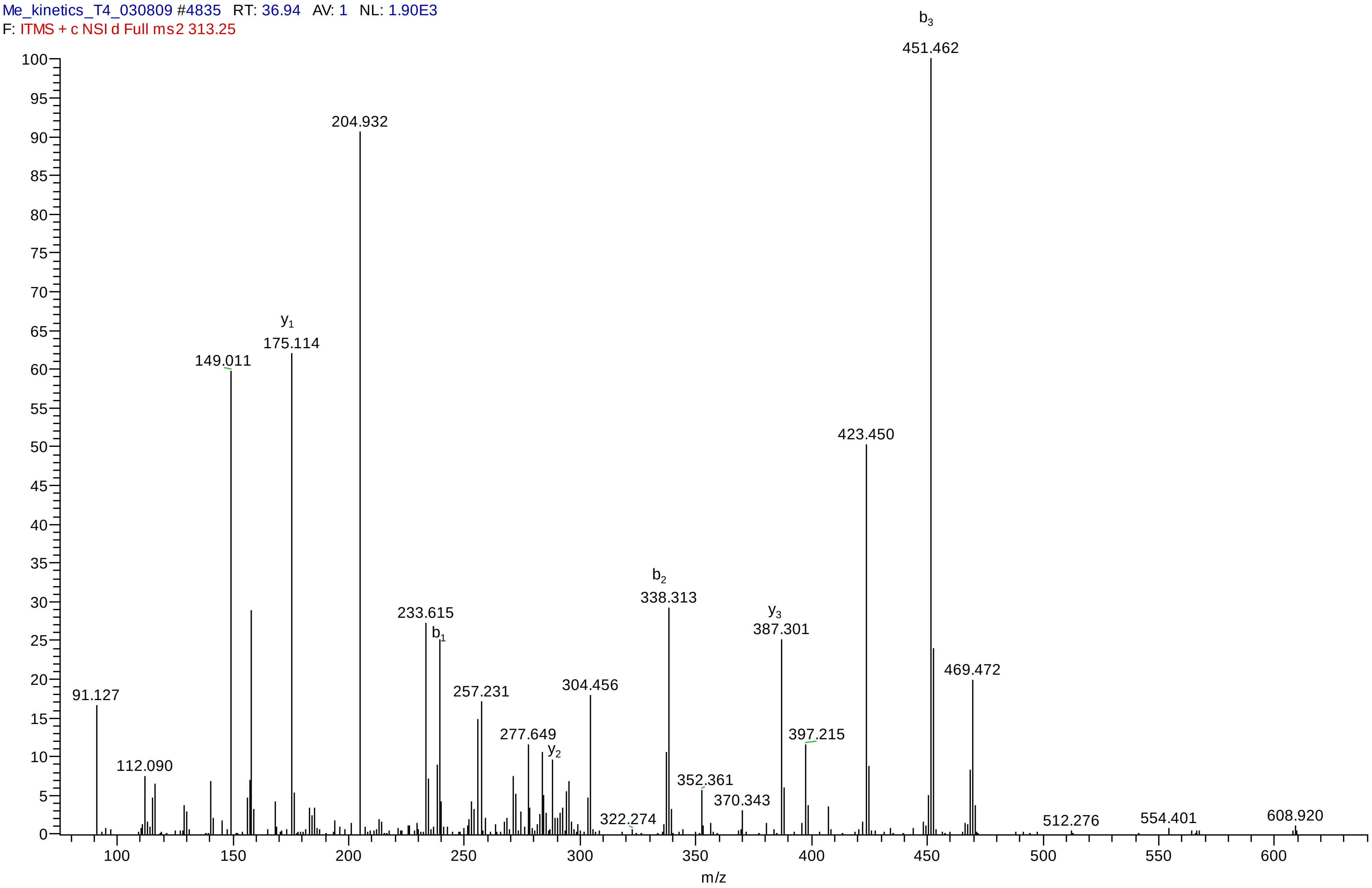

Supplement: Additional file 1 — Figure S1: Tandem mass spectrometry (MS/MS) spectra of labeled peptides analyzed. MS/MS spectra of partially and fully 13C615N2-lysine labeled peptides, where the red and blue peaks correspond to the b and y ions annotated by Bioworks Browser. [file 1756-8935-3-22-S1.PDF]
