# Supplementary material for: Global turnover of histone post-translational modifications and variants in human cells
Source: Epigenetics Chromatin. 2010 Dec 6;3:22. doi: 10.1186/1756-8935-3-22 (PMC3004898; doi:10.1186/1756-8935-3-22)
Supplement: Additional file 2 — Table S1: List of all histone peptides analyzed: All histone peptides quantified in our experiment are provided below, with their charge state, expected and observed mass to charge ratio (m/z). ac = Acetyl, me = methyl, ox = oxidation pr = propionyl, un = unmodified. *Charge state of the peptide is included; †unable to differentiate based on MS/MS. [file 1756-8935-3-22-S2.PDF]

Additional File 2 Table 1

**Peptide sequence**	**Simplified notation***	**Expected m/z**	**Observed m/z**
**H1.4**			
$_{pr}K_{pr}ASGPPVSELITK_{pr}AVAASK_{pr}ER$	H1.4 3:0 +3	788.445	788.445
	H1.4 3:2 +3	793.785	793.789
	H1.4 3:3 +3	796.455	796.459
**H2A**			
$_{pr}ATIAGGGVIPHIHK_{pr}$	H2A1 1:0 +2	741.926	741.927
	H2A1 1:1 +2	745.931	745.934
$_{pr}K_{pr}GNYAER$	H2A2 1:0 +2	475.241	475.241
	H2A2 1:1 +2	479.246	479.248
$_{pr}K_{pr}GNYSER$	H2A3 1:0 +1	965.469	965.469
	H2A3 1:0 +2	483.239	483.238
	H2A3 1:1 +1	973.480	973.483
	H2A3 1:1 +2	487.244	487.245
$_{pr}GK_{pr}QGGK_{pr}AR$ 2:0	H2A4 2:0 +2	485.278	485.277
	H2A4 2:1 +2	489.283	489.285
	H2A4 2:2 +2	493.289	493.291
$_{pr}NDEELNK_{pr}LLGR$	H2A5 1:0 +1	1412.738	1412.737
	H2A5 1:0 +2	706.873	706.873
	H2A5 1:1 +1	1420.749	1420.751
	H2A5 1:1 +2	710.878	710.880
**H2B**			
$_{pr}PEPAK_{pr}$	H2B1 1:0	653.351	653.351
	H2B1 1:1	661.364	661.364
$_{pr}LAHYNK_{pr}R$	H2B2 1:0 +2	507.281	507.281
	H2B2 1:1 +2	511.286	511.287
**H3**			
$_{pr}TK_{pr}QTAR$	H3K4un 1:0 +2	408.733	408.733
	H3K4un 1:1 +2	412.738	412.740
$_{pr}TK_{me1,pr}QTAR$	H3K4me1 1:0 +2	415.741	415.740
	H3K4me1 1:1 +2	419.746	419.747
$_{pr}K_{pr}STGGK_{pr}APR$	H3K9un 2:0 +2	535.304	535.304
	H3K9un 2:1 +2	539.310	539.311
	H3K9un 2:2 +2	543.315	543.317
$_{pr}K_{pr,me1}STGGK_{pr}APR$	H3K9me1 2:0 +2	542.312	542.312
	H3K9me1 2:1 +2	546.317	546.318
	H3K9me1 2:2 +2	550.323	550.326
$_{pr}K_{me2}STGGK_{pr}APR$	H3K9me2 2:0 +2	521.307	521.307
	H3K9me2 2:1 +2	525.312	525.313
	H3K9me2 2:2 +2	529.317	529.320
$_{pr}K_{me3}STGGK_{pr}APR$	H3K9me3 2:0 +2	528.315	528.314

	H3K9me3 2:1 +2	532.320	532.321
	H3K9me3 2:2 +2	536.325	536.328
$_{pr}K_{ac}STGGK_{pr}APR$† $_{pr}K_{pr}STGGK_{ac}APR$†	H3K9/K14ac1 2:0 +2	528.296	528.296
	H3K9/K14ac1 2:1 +2	532.301	532.303
	H3K9/K14ac1 2:2 +2	536.307	536.309
$_{pr}K_{pr,me1}STGGK_{ac}APR$	H3K9me1K14ac1 2:0 +2	535.304	535.304
	H3K9me1K14ac1 2:1 +2	539.309	539.311
	H3K9me1K14ac1 2:2 +2	543.315	543.317
$_{pr}K_{me2}STGGK_{ac}APR$	H3K9me2K14ac1 2:0 +2	514.299	514.298
	H3K9me2K14ac1 2:1 +2	518.304	518.305
	H3K9me2K14ac1 2:2 +2	522.309	522.312
$_{pr}K_{pr}QLATK_{pr}AAR$	H3K18K23un 2:0 +2	577.849	577.850
	H3K18K23un 2:1 +2	581.856	581.857
	H3K18K23un 2:2 +2	585.863	585.863
$_{pr}K_{ac}QLATK_{pr}AAR$† $_{pr}K_{pr}QLATK_{ac}AAR$†	H3K18/K23ac1 2:0 +2	570.841	570.842
	H3K18/K23ac1 2:1 +2	574.846	574.848
	H3K18/K23ac1 2:2 +2	578.851	578.855
$_{pr}K_{ac}QLATK_{ac}AAR$	H3K18ac1K23ac1 2:0 +2	563.833	563.832
	H3K18ac1K23ac1 2:1 +2	567.838	567.840
	H3K18ac1K23ac1 2:2 +2	571.843	571.845
$_{pr}K_{pr}SAPATGGVK_{pr}K_{pr}PHR$	H3K27K36un 3:0 +3	553.318	553.318
	H3K27K36un 3:1 +3	555.988	555.990
	H3K27K36un 3:2 +3	558.659	558.661
	H3K27K36un 3:3 +3	561.329	561.332
$_{pr}K_{pr,me1}SAPATGGVK_{pr}K_{pr}PHR$	H3K27me1 3:0 +3	557.990	557.989
	H3K27me1 3:1 +3	560.660	560.662
	H3K27me1 3:2 +3	563.331	563.332
	H3K27me1 3:3 +3	566.001	566.003
$_{pr}K_{pr}SAPATGGVK_{pr,me1}K_{pr}PHR$	H3K36me1 3:0 +3	557.990	557.990
	H3K36me1 3:1 +3	560.660	560.660
	H3K36me1 3:2 +3	563.331	563.331
	H3K36me1 3:3 +3	566.001	566.004
$_{pr}K_{me2}SAPATGGVK_{pr}K_{pr}PHR$	H3K27me2 3:0 +3	543.987	543.987
	H3K27me2 3:1 +3	546.657	546.657
	H3K27me2 3:2 +3	549.327	549.327
	H3K27me2 3:3 +3	551.997	552.000
$_{pr}K_{pr}SAPATGGVK_{me2}K_{pr}PHR$	H3K36me2 3:0 +3	543.987	543.987
	H3K36me2 3:1 +3	546.657	546.659
	H3K36me2 3:2 +3	549.327	549.327
	H3K36me2 3:3 +3	551.997	552.000
$_{pr}K_{me3}SAPATGGVK_{pr}K_{pr}PHR$	H3K27me3 3:0 +3	548.658	548.659
	H3K27me3 3:1 +3	551.329	551.330
	H3K27me3 3:2 +3	553.999	554.001

	H3K27me3 3:3 +3	556.669	556.671
$_{pr}K_{me2}SAPATGGVK_{pr,me1}K_{pr}PHR$	H3K27me2K36me1 3:0 +3	548.658	548.659
	H3K27me2K36me1 3:1 +3	551.329	551.330
	H3K27me2K36me1 3:2 +3	553.999	554.000
	H3K27me2K36me1 3:3 +3	556.669	556.672
$_{pr}K_{pr,me1}SAPATGGVK_{me2}K_{pr}PHR$	H3K27me1K36me2 3:0 +3	548.658	548.659
	H3K27me1K36me2 3:1 +3	551.329	551.328
	H3K27me1K36me2 3:2 +3	553.999	553.999
	H3K27me1K36me2 3:3 +3	556.669	556.672
$_{pr}K_{me2}SAPATGGVK_{me2}K_{pr}PHR$	H3K27me2K36me2 3:0 +3	534.655	534.654
	H3K27me2K36me2 3:2 +3	539.995	539.996
	H3K27me2K36me2 3:3 +3	542.666	542.668
$_{pr}K_{me3}SAPATGGVK_{pr,me1}K_{pr}PHR$	H3K27me3K36me1 3:0 +3	553.330	553.331
	H3K27me3K36me1 3:2 +3	558.671	558.673
	H3K27me3K36me1 3:3 +3	561.341	561.343
$_{pr}K_{pr,me1}SAPATGGVK_{me3}K_{pr}PHR$	H3K27me1K36me3 3:0 +3	553.330	553.330
	H3K27me1K36me3 3:1 +3	556.000	556.004
	H3K27me1K36me3 3:2 +3	558.671	558.672
	H3K27me1K36me3 3:3 +3	561.341	561.343
$_{pr}K_{pr}SAPSTGGVK_{pr}K_{pr}PHR$	H3.3K27K36un 3:0 +3	558.649	558.649
	H3.3K27K36un 3:2 +3	563.990	563.992
	H3.3K27K36un 3:3 +3	566.661	566.664
$_{pr}K_{me2}SAPSTGGVK_{pr}K_{pr}PHR$	H3.3K27me2 3:0 +3	549.318	549.318
	H3.3K27me2 3:2 +3	554.659	554.661
	H3.3K27me2 3:3 +3	557.329	557.332
$_{pr}K_{pr}SAPSTGGVK_{me2}K_{pr}PHR$	H3.3K36me2 3:0 +3	549.318	549.319
	H3.3K36me2 3:2 +3	554.659	554.661
	H3.3K36me2 3:3 +3	557.329	557.333
$_{pr}K_{me3}SAPSTGGVK_{pr}K_{pr}PHR$	H3.3K27me3 3:0 +3	553.989	553.989
	H3.3K27me3 3:2 +3	559.330	559.331
	H3.3K27me3 3:3 +3	562.001	562.004
$_{pr}K_{me2}SAPSTGGVK_{pr,me1}K_{pr}PHR$	H3.3K27me2K36me1 3:0 +3	553.989	553.990
	H3.3K27me2K36me1 3:2 +3	559.330	559.331
	H3.3K27me2K36me1 3:3 +3	562.001	562.004
$_{pr}K_{pr,me1}SAPSTGGVK_{me2}K_{pr}PHR$	H3.3K27me1K36me2 3:0 +3	553.989	553.990
	H3.3K27me1K36me2 3:2 +3	559.330	559.330
	H3.3K27me1K36me2 3:3 +3	562.001	562.004
$_{pr}K_{pr}LPFQR$	H3K65un 1:0 +1	900.531	900.531
	H3K65un 1:0 +2	450.769	450.769
	H3K65un 1:1 +1	908.541	908.544
	H3K65un 1:1 +2	454.775	454.776
$_{pr}EIAQDFK_{pr}TDLR$	H3K79un 1:0 +2	724.376	724.376
	H3K79un 1:1 +2	728.381	728.382
$_{pr}EIAQDFK_{pr,me1}TDLR$	H3K79me1 1:0 +2	731.383	731.383
	H3K79me1 1:1 +2	735.390	735.391

$_{pr}$EIAQDFK$_{me2}$TDLR	H3K79me2 1:0 +2	710.378	710.378
	H3K79me2 1:1 +2	714.385	714.386
$_{pr}$VTIMPK$_{pr}$DIQLAR	H3K122un 1:0 +2	748.929	748.930
	H3K122un 1:1 +2	752.937	752.937
$_{pr}$VTIM$_{ox}$PK$_{pr}$DIQLAR	H3K122ox 1:0 +2	756.927	756.928
	H3K122ox 1:1 +2	760.934	760.934
**H4**			
$_{pr}$GK$_{pr}$GGK$_{pr}$GLGK$_{pr}$GGAK$_{pr}$R	H4K5K8K12K16un 4:0 +2	775.955	775.956
	H4K5K8K12K16un 4:2 +2	783.966	783.968
	H4K5K8K12K16un 4:3 +2	787.971	787.975
	H4K5K8K12K16un 4:4 +2	791.976	791.983
$_{pr}$GK$_{ac}$GGK$_{pr}$GLGK$_{pr}$GGAK$_{pr}$R†<br>$_{pr}$GK$_{pr}$GGK$_{ac}$GLGK$_{pr}$GGAK$_{pr}$R†<br>$_{pr}$GK$_{pr}$GGK$_{pr}$GLGK$_{ac}$GGAK$_{pr}$R†<br>$_{pr}$GK$_{pr}$GGK$_{pr}$GLGK$_{pr}$GGAK$_{ac}$R†	H4K5/K8/K12/K16ac1 4:0 +2	768.947	768.947
	H4K5/K8/K12/K16ac1 4:2 +2	776.957	776.960
	H4K5/K8/K12/K16ac1 4:3 +2	780.963	780.967
	H4K5/K8/K12/K16ac1 4:4 +2	784.968	784.974
$_{pr}$GK$_{ac}$GGK$_{ac}$GLGK$_{pr}$GGAK$_{pr}$R†<br>$_{pr}$GK$_{ac}$GGK$_{pr}$GLGK$_{ac}$GGAK$_{pr}$R†<br>$_{pr}$GK$_{ac}$GGK$_{pr}$GLGK$_{pr}$GGAK$_{ac}$R†<br>$_{pr}$GK$_{pr}$GGK$_{ac}$GLGK$_{ac}$GGAK$_{pr}$R†<br>$_{pr}$GK$_{pr}$GGK$_{ac}$GLGK$_{pr}$GGAK$_{ac}$R†<br>$_{pr}$GK$_{pr}$GGK$_{pr}$GLGK$_{ac}$GGAK$_{ac}$R†	H4K5/K8/K12/K16ac2 4:0 +2	761.939	761.939
	H4K5/K8/K12/K16ac2 4:2 +2	769.949	769.950
	H4K5/K8/K12/K16ac2 4:3 +2	773.954	773.958
	H4K5/K8/K12/K16ac2 4:4 +2	777.960	777.966
$_{pr}$GK$_{ac}$GGK$_{ac}$GLGK$_{ac}$GGAK$_{pr}$R†<br>$_{pr}$GK$_{pr}$GGK$_{ac}$GLGK$_{ac}$GGAK$_{ac}$R†<br>$_{pr}$GK$_{ac}$GGK$_{pr}$GLGK$_{ac}$GGAK$_{ac}$R†<br>$_{pr}$GK$_{ac}$GGK$_{ac}$GLGK$_{pr}$GGAK$_{ac}$R†	H4K5/K8/K12/K16ac3 4:0 +2	754.931	754.930
	H4K5/K8/K12/K16ac3 4:3 +2	766.947	766.951
	H4K5/K8/K12/K16ac3 4:4 +2	770.952	770.958
$_{pr}$GK$_{ac}$GGK$_{ac}$GLGK$_{ac}$GGAK$_{ac}$R	H4K5K8K12K16ac4 4:0 +2	747.922	747.922
	H4K5K8K12K16ac4 4:3 +2	759.939	759.937
	H4K5K8K12K16ac4 4:4 +2	763.944	763.943
$_{pr}$K$_{pr}$VLR	H4K20un 1:0 +1	627.419	627.419
	H4K20un 1:0 +2	314.214	314.213
	H4K20un 1:1 +1	635.433	635.433
	H4K20un 1:1 +2	318.221	318.220
$_{pr}$K$_{pr,me1}$VLR	H4K20me1 1:0 +1	641.435	641.435
	H4K20me1 1:0 +2	321.221	321.221
	H4K20me1 1:1 +1	649.449	649.449
	H4K20me1 1:1 +2	325.228	325.228
$_{pr}$K$_{me2}$VLR	H4K20me2 1:0 +1	599.424	599.424

	H4K20me2 1:0 +2	300.216	300.216
	H4K20me2 1:1 +2	304.223	304.223
${}_{pr}K_{me3}VLR$	H4K20me3 1:0 +1	613.440	613.439
	H4K20me3 1:0 +2	307.224	307.223
	H4K20me3 1:1 +1	621.451	621.453
	H4K20me3 1:1 +2	311.231	311.230
${}_{pr}DNIQGITK_{pr}PAIR$	H4K31un 1:0 +2	719.407	719.408
	H4K31un 1:1 +2	723.414	723.415
