# Supplementary figures and images for: Global turnover of histone post-translational modifications and variants in human cells
Source: Epigenetics Chromatin. 2010 Dec 6;3:22. doi: 10.1186/1756-8935-3-22 (PMC3004898; doi:10.1186/1756-8935-3-22)

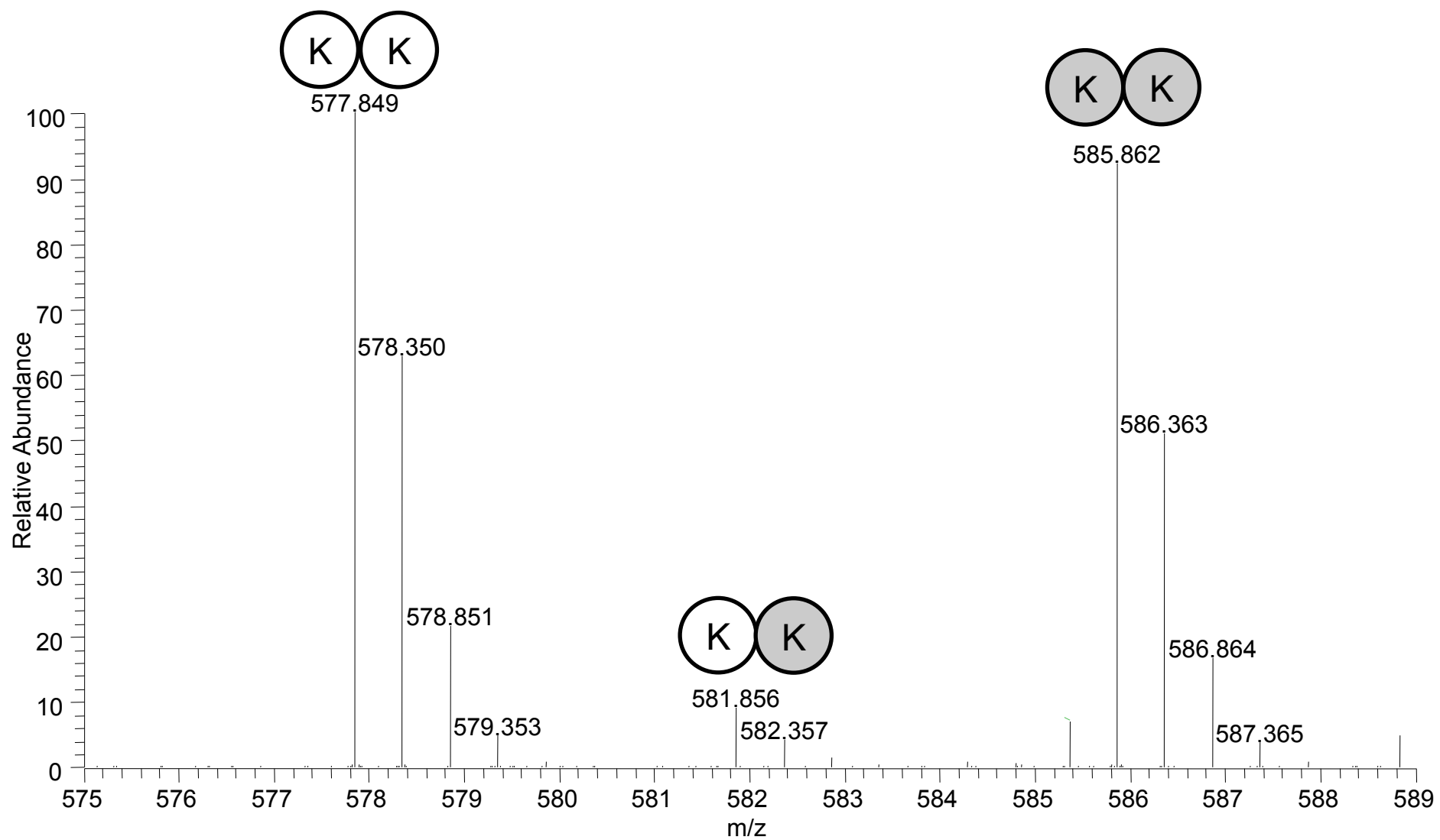

**Additional File 3**  
**Figure 2**

Supplement: Additional file 3 — Figure S2: Detection of labeled isotopes. Mass spectrometry spectrum of the unmodified H3 18-26 peptide after 1 day of 13C615N2-lysine labeling, where the isotopic distributions for the peptide containing two 12C614N2-lysines, one 12C614N2-lysine and one 13C615N2-lysine, and two 13C615N2-lysines are detected. [file 1756-8935-3-22-S3.PDF]

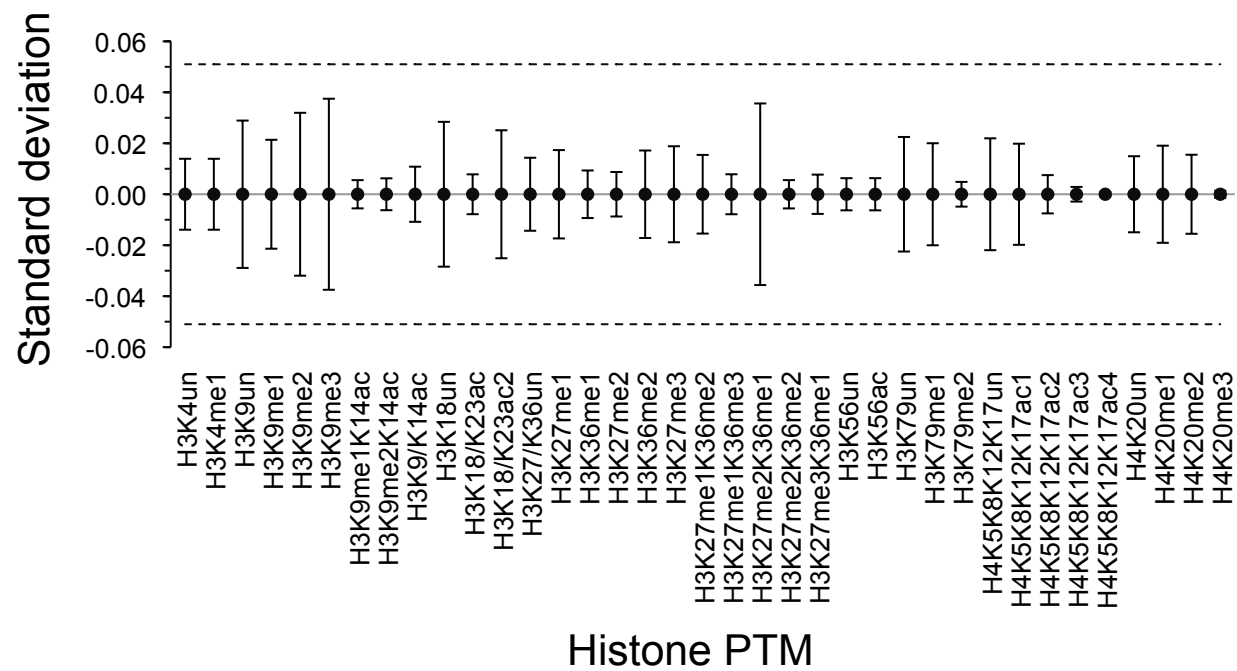

**Additional File 5**  
**Figure 4**

Supplement: Additional file 5 — Figure S4: Steady state assumption. Steady state levels for histone post-translational modifications. Standard deviations (vertical bars) of the relative abundances for the H3 and H4 peptides across the labeling time course are shown relative to their respective means (black circles). Horizontal dashed lines denote a standard deviation of 0.051, the threshold at which 95% of the observed variability cannot be accounted by 5-10% of the instrument measurement variability. ac = Acetyl, me = methyl, un = unmodified. [file 1756-8935-3-22-S5.PDF]

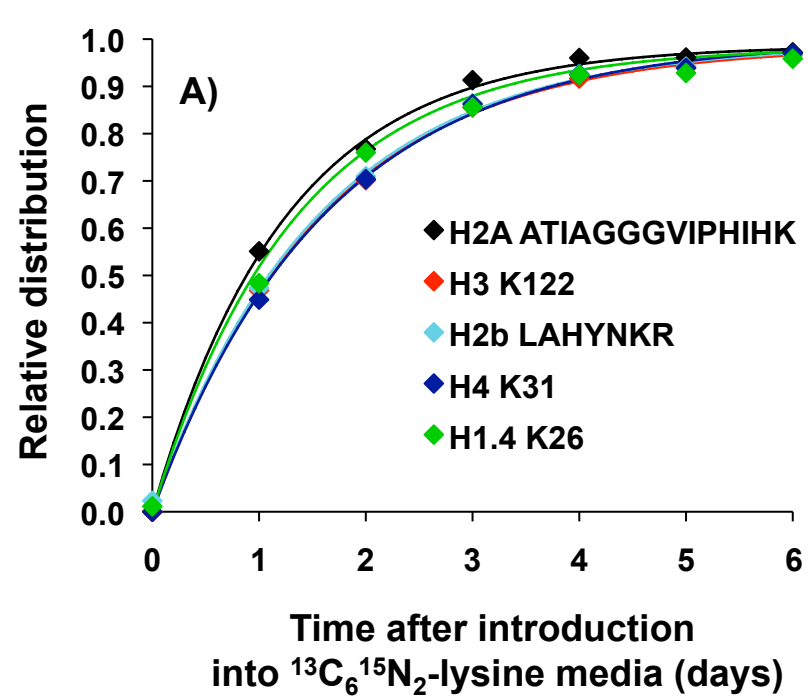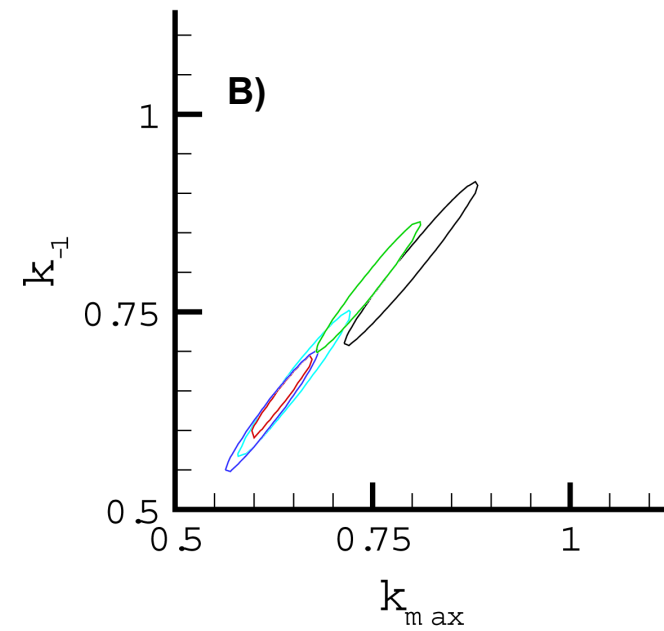

Additional File 6  
Figure 5

Supplement: Additional file 6 — Figure S5: Comparison of turnover between histones. (A) Relative distribution of the fully labeled H2A variant (black diamond), H3 (green diamond), H2b variant (pink diamond), H4 (dark blue diamond) and H1.4 (light blue diamond) core peptides during the time course. Lines represent fits based on the optimized kinetic parameters for the respective peptides. (B) Contour plots of the kinetic parameters for the respective core peptides (same color scheme as in (A)), where kmax = k1 for H2A, H2B, H3 and H4, and k2 for H1.4. The limits of the contour plots are defined by z(k) × (1+2/5 × F0.05(2,5)) for all the histone peptides except the H1.4 peptide, where the limit is defined by z(k) × (1+3/11 × F0.05(3,11)) because of the additional lysine on the H1.4 peptide. [file 1756-8935-3-22-S6.PDF]

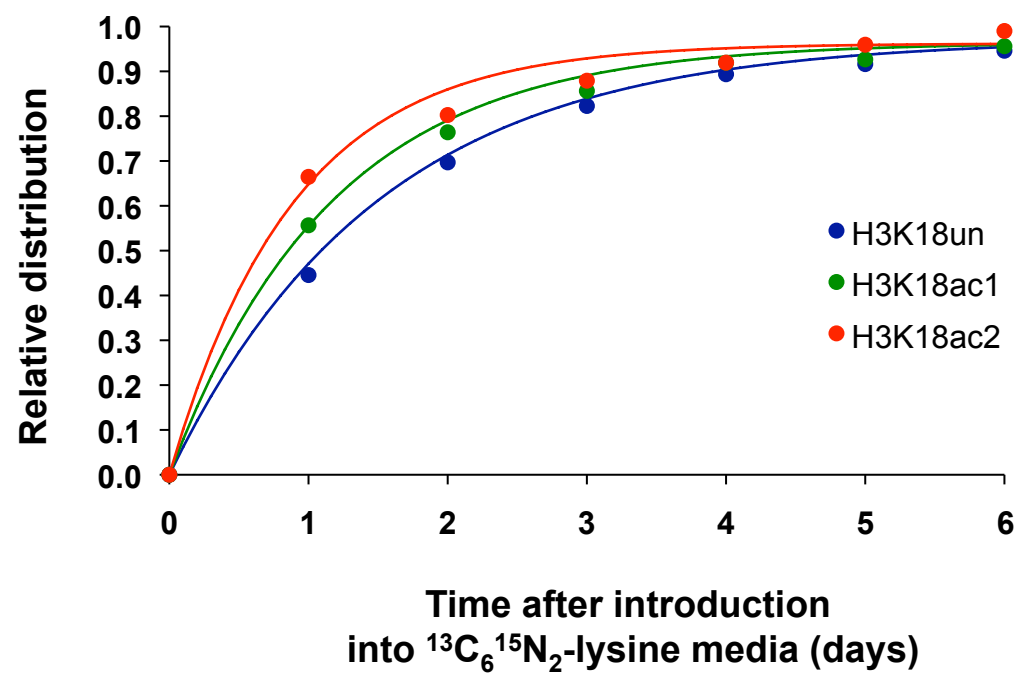

Additional File 7  
Figure 6

Supplement: Additional file 7 — Figure S6: Progressive modifications and turnover. Turnover modeling (colored lines) of the relative distribution of the fully labeled unmodified (H3K18un, blue circle), monoacetylated (H3K18/K23ac1, green circle) and diacetylated (H3K18ac1K23ac1, red circle) H3 18-26 peptides across the labeling time course. Note the increasingly faster accumulation of the fully labeled peptides (increasingly leftward shift) as acetylation increases. [file 1756-8935-3-22-S7.PDF]
