# Supplementary material for: Global turnover of histone post-translational modifications and variants in human cells
Source: Epigenetics Chromatin. 2010 Dec 6;3:22. doi: 10.1186/1756-8935-3-22 (PMC3004898; doi:10.1186/1756-8935-3-22)
Supplement: Additional file 4 — Figure S3: Model for histone turnover. Set of differential equations that extrapolate histone turnover for a peptide containing one, two, three and four lysines. We found that at steady state, the rate of histone removal for a particular labeled state should equal the sum of all the rates of histone addition. This provides a constraint in the optimization procedure. Furthermore, because the rate of addition = rate of removal, k-1 becomes a measure of overall turnover for that peptide. For a peptide containing two lysines, we needed to include an additional factor of 2 for k1 because we were unable to differentiate which of the two lysines are isotopically labeled. For similar reasons, we modifid the differential equations for peptides containing more than one lysine. P# = relative abundance of peptide, where # indicates the number of labeled lysines. k-1 = Rate of histone removal. k# = Rate of histone addition, where # indicates the number of labeled lysines. [file 1756-8935-3-22-S4.PDF]

Additional File 4 Figure 3.

For a peptide with one lysine:
$dP_0/dt = k_0 + k_{-1}P_0 = k_0 + (k_0+k_1)P_0$
$dP_1/dt = k_1 + k_{-1}P_1 = k_1 + (k_0+k_1)P_1$

For a peptide with two lysines:
$dP_0/dt = k_0 + k_{-1}P_0 = k_0 + (k_0+2k_1+k_2)P_0$
$dP_1/dt = 2k_1 + k_{-1}P_1 = k_1 + (k_0+2k_1+k_2)P_1$
$dP_2/dt = k_2 + k_{-1}P_2 = k_2 + (k_0+2k_1+k_2)P_2$

For a peptide with three lysines:
$dP_0/dt = k_0 + k_{-1}P_0 = k_0 + (k_0+3k_1+3k_2+k_3)P_0$
$dP_1/dt = 3k_1 + k_{-1}P_1 = k_1 + (k_0+3k_1+3k_2+k_3)P_1$
$dP_2/dt = 3k_2 + k_{-1}P_2 = k_2 + (k_0+3k_1+3k_2+k_3)P_2$
$dP_3/dt = k_3 + k_{-1}P_3 = k_3 + (k_0+3k_1+3k_2+k_3)P_3$

For a peptide with four lysines:
$dP_0/dt = k_0 + k_{-1}P_0 = k_0 + (k_0+4k_1+6k_2+4k_3+k_4)P_0$
$dP_1/dt = 4k_1 + k_{-1}P_1 = k_1 + (k_0+4k_1+6k_2+4k_3+k_4)P_1$
$dP_2/dt = 6k_2 + k_{-1}P_2 = k_2 + (k_0+4k_1+6k_2+4k_3+k_4)P_2$
$dP_3/dt = 4k_3 + k_{-1}P_3 = k_3 + (k_0+4k_1+6k_2+4k_3+k_4)P_3$
$dP_4/dt = k_4 + k_{-1}P_4 = k_4 + (k_0+4k_1+6k_2+4k_3+k_4)P_4$
