# Supplementary material for: Global turnover of histone post-translational modifications and variants in human cells
Source: Epigenetics Chromatin. 2010 Dec 6;3:22. doi: 10.1186/1756-8935-3-22 (PMC3004898; doi:10.1186/1756-8935-3-22)
Supplement: Additional file 8 — Figure S7: Confidence regions for parameter estimates. For each pair of estimated rate constants, we compared confidence regions using the F-test, which is presented below, where k is the vector containing the rate constants (that is, k0, k1,..., kN), k^is the vector of estimates for these rate constants as determined by solving the regression problem, z(k) is the value of the objective function for the regression problem (that is, the sum of the squared differences between the predicted and actual relative abundances), and Fp,n−pα is the upper α critical value for the F distribution for p parameters and n data points. Thus, the corresponding confidence region for a given estimate k^ is the union of all k values that satisfy equation 1. [file 1756-8935-3-22-S8.PDF]

Additional File 8 Figure 7

$$\left\{k : z(k) \leq z\left(\hat{k}\right) + z\left(\hat{k}\right) \cdot \frac{p}{n-p} F^{\alpha}_{p,n-p}\right\} \quad (1)$$
